# HIF-1α Alleviates High-Glucose-Induced Renal Tubular Cell Injury by Promoting Parkin/PINK1-Mediated Mitophagy

**DOI:** 10.3389/fmed.2021.803874

**Published:** 2022-02-03

**Authors:** Lu Yu, Yulin Wang, Yan Hong Guo, Liuwei Wang, Zijun Yang, Zi Han Zhai, Lin Tang

**Affiliations:** ^1^First Affiliated Hospital of Zhengzhou University, Zhengzhou, China; ^2^College of Public Health, Zhengzhou University, Zhengzhou, China

**Keywords:** HIF-1α, mitophagy, diabetic nephropathy, inflammation, ROS, apoptosis

## Abstract

It is well-established that mitophagy leads to Diabetic Nephropathy (DN) and renal failure. Mitophagy mediated by a Hypoxia-inducible factor-1α (HIF-1α) plays a beneficial role in many diseases. Nevertheless, the mechanisms underlying HIF-1α-mediated mitophagy in DN remain unclear. This study defines the role of HIF-1α mediated mitophagy in DN. The expression of HIF-1α was upregulated in HK-2 cells in an High-Glucose (HG) environment, and the YC-1 (a specific inhibitor of HIF-1α) further exacerbated the hypoxia-induced mitochondrial dysfunction. Conversely, the HIF-1α-mediated protective effect was strengthened by scavenger N-acetylcysteine (NAC), a type of reactive oxygen species. Moreover, HIF-1α-Parkin/PINK1-mediated mitophagy prevented apoptosis and ROS production in HK-2 cells subjected to HG exposure. In summary, HIF-1α mediated mitophagy on HK-2 cells under HG conditions could alleviate DN, suggesting that it has huge prospects for DN treatment.

## Introduction

The development of the global economy has been paralleled by lifestyle changes, resulting in an elevated incidence of type 2 diabetes mellitus, which has gradually became a serious public health and safety concern worldwide (1). One major complication of diabetes mellitus is the Diabetic nephropathy (DN) (2), which is the primary cause of an end-stage renal disease (3), not only seriously affecting the quality of life of the patient but also imposes a heavy financial burden on society (4). Regrettably, the etiopathogenesis of DN remains to be elucidated, and therapy is limited by the paucity of effective drugs. Accordingly, it is of great significance to thoroughly explore the pathogenesis of DN and to further search for effective therapeutic targets.

Diabetic tubulopathy has an extensive-expression in DN development which contributes to initial renal injury in the pathogenesis of DN (5, 6). Renal tubular hypoxia is reportedly a pathological change found in the early and advanced stages of DN (7), eing an important pathogenic factor causing renal fibrosis. Hypoxia-inducible factor (HIF)-1 is a pivotal molecule that plays an important part in a hypoxic environment. The HIF-1 is widely acknowledged as a heterodimeric transcriptional factor consisting of an oxygen-sensitive α subunit and constitutively expressed β subunit (8–10). An increasing body of evidence suggests that high glucose levels upregulate HIF-1α expression in animal models and human renal proximal tubular cells of type 2 diabetes mellitus nephropathy (11–13). Nevertheless, the precise role of HIF-1α of diabetic nephropathy in the etiopathogenesis remains unclear.

Mitophagy is a special type of autophagy that was first proposed by LeMasters in 2005 (14). Mitophagy selectively clears damaged or unwanted mitochondria and fuses with lysosomes to degrade the redundant mitochondria through different signaling pathways, such as PINK1/parkin and Nix/BNIP3 (6, 15), to maintain the reactive oxygen species (ROS) balance (16). Mitochondrial dysfunction has been documented as an important mechanism in the pathogenesis of renal tubular injury in DN (17, 18), leading to a dysfunctional mitochondrial accumulation, excessive ROS production, renal tubular cell injury, and apoptosis (19). Treatment with mitochondria-targeted antioxidants, such as mitoQ, has been reported to alleviate mitophagy and tubular injury (20). Moreover, studies have corroborated that HIF1α-mediated mitophagy plays a beneficial role in acute kidney injury by inhibiting tubular cell apoptosis and ROS production (21, 22). Nevertheless, whether HIF1α alleviates renal tubular cell injury by promoting mitophagy in an HG environment remains unknown.

In the present study, the renal tubular epithelial cells were subjected to HG exposure. This study explores the effects of HIF1α-mediated mitophagy and ascertain whether the Parkin/PINK1 signaling pathway was involved in this process.

## Materials and Methods

### Cell Culture and Treatment

Human proximal tubular epithelial cells HK-2 were cultured in DMEM/F-12 medium supplemented with 10% fetal bovine serum (FBS, Gibco), 1,000 U/L penicillin, and 100 μg/ml streptomycin at 37°C in 5% CO2 air. The cells were treated with normal glucose (NG, 5.5 mM D-glucose), high-glucose (HG, 30 mM D-glucose) for 24 h with or without 10 μM HIF1A inhibitor YC-1, and ROS scavenger NAC (Sigma-Aldrich, A7250, Germany) at a final concentration of 5 mM.

### Detection of Mitochondrial ROS Production

To assess mitochondrial superoxide production, HK-2 cells were incubated with MitoSOX (0.5 μM mol/L, Thermo Fisher, United States) for 15 min. The MitoSOX fluorescence images were obtained by a fluorescence microscope (Olympus CKX53, Japan). The maximum excitation wavelength of MitoSOX was set at 510 nm, and the discharge wavelength was 552–620 nm. Image J software was used to analyze the average fluorescent intensity.

### Immunofluorescence

The HK-2 cells were treated with adenovirus expressing GFP-LC3. Two days later, the samples were fixed with 4% paraformaldehyde, permeated with methanol/acetone, and infected with TOM20 antibody (1:200, Abcam, ab186734, United States). The samples were then observed under a fluorescence confocal microscope (LSM510 META, Karl Zeiss, Germany).

### Western Blot Analysis

Cells were dissolved and lysed in a radioimmunoprecipitation (RIPA) buffer with protease after centrifuging at 13,000 g for 10 min. Mitochondrial fragments were collected using an isolation kit (Nanjing Jiancheng Bioengineering Inc., China). The antibodies used in this study are as follows: Anti-VDAC (1:4000, Abcam, ab14734), Anti-LC3II (1:1500, CST, #3868), Anti-Parkin (1:1000, Abcam, ab77924), Anti-PINK1 (1:1500, Abcam, ab216144), Anti-SQSTM1/P62 (1:5000, CST, #5114), Anti-HIF2a (1:1000, CST, #57921), Anti-HIF1a (1:2000, CST, #3716), Anti-E-Cadherin (1:2000, CST, #14472), Anti-Fibronectin/FN (1:2000, CST, #26836), Anti-a-SMA (1:2000, CST, # 19245), and Anti-SGLT1 (1:2000, Abcam, ab14686). The experiments were repeated more than three times, and the images were captured after incubation in an enhanced chemiluminescence (ECL) reagent. The density of the labeled protein bands which were normalized to β-actin was quantified with a Quantity One software.

### Apoptosis Assay

Cells were seeded in 6-well plates for 24 h and then transfected with the indicated plasmids. After 24–36 h, the cells were collected and washed with ice-cold PBS 3 times and gently resuspended in a 500 μl binding buffer. Then, the cells were stained with Annexin V/FITC. Subsequently, Propidium iodide (PI) was added into the buffer and incubated for another 10 min in the dark. Finally, the stained cells were analyzed using CytoFLEX (BECKMAN COULTER).

### Enzyme-Linked Immunosorbent Assay

IL-1β and IL-18 levels were quantitatively measured using ELISA test kits (Dakewe, Shenzhen, China; ABclonal, Wuhan, China) in accordance with the instructions of the manufacturer. The absorbance was measured with a Thermo Scientific microplate reader (Model 680, Bio-Rad, Hercules, CA, USA) at 450 nm.

### Statistical Analysis

The data were expressed as mean ± SEM. The unpaired two-tailed *t*-test was used for comparison between two groups. Analysis of variance or repeated analysis of variance was used for multiple comparisons. Graphpad prism^®^ 6.0 software was used for statistical analysis.

## Results

### Effect of HIF-1α on Mitophagy-Related Proteins in HK-2 Cells Subjected to HG Exposure

**Figure 1:**
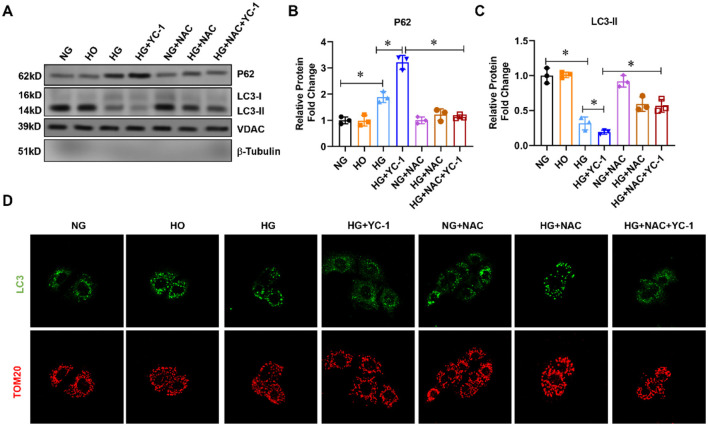
Effect of HIF-1α on mitophagy-related proteins in HK-2cells subjected to HG exposure. **(A–C)**. The expression of p62 and LC3-II was assessed by Western blot in HK-2 cells treated with normal glucose, high-glucose for 24 h with or without 10 μM HIF-1α inhibitor YC-1, and reactive oxygen species (ROS) scavenger N-Acetyl-L-Cysteine (NAC) at a final concentration of 5 mm.*p < 0.05. vs. indicated group. **(D)** Mitophagy was assessed using a fluorescence confocal microscope. HG, High Glucose; LC3-II, Microtubule-Associated-Proteinlight-Chain-3 II; HIF-1α, Hypoxia-Inducible Factor-1; YC-1, Lificiguat; ROS, Reactive Oxygen Species; NAC, N-Acetyl-L-Cysteine.

To observe the effect of HIF-1α on mitochondrial autophagy under high glucose conditions, we first extracted the mitochondrial component protein from the cell homogenate and then carried out the western blot experiments. The LC3-II was downregulated while the autophagy substrate p62 was upregulated in the high-glucose group compared to the normal glucose group (Figure 1A), suggesting that high glucose could potently inhibit mitophagy. After specific inhibition of HIF-1α with YC-1, mitophagy activation levels were decreased in the HG + YC-1 group compared with the HG group, with reduced LC3-II levels and increased p62 levels (Figure 1A). Given the role of ROS in the regulation of mitophagy, the reactive oxygen species scavenger NAC was added to explore whether HIF-1α regulates mitophagy in HK-2 cells under high-glucose conditions. Importantly, the inhibitory effect of high glucose and YC-1 on mitochondrial autophagy was significantly reversed by NAC treatment (Figure 1A). Confocal microscopy showed that the binding strength between LC3-II and mitochondria was decreased in HK-2 cells under high-glucose conditions and was enhanced by YC-1 treatment but reversed by NAC (Figure 1D). These results indicate that HIF-1α may play a role in mitochondrial autophagy in HK-2 cells exposed to the HG environment.

### HIF-1α Activated the Parkin/PINK1 Signaling Pathway in HK-2 Cells Subjected to HG Exposure

**Figure 2:**
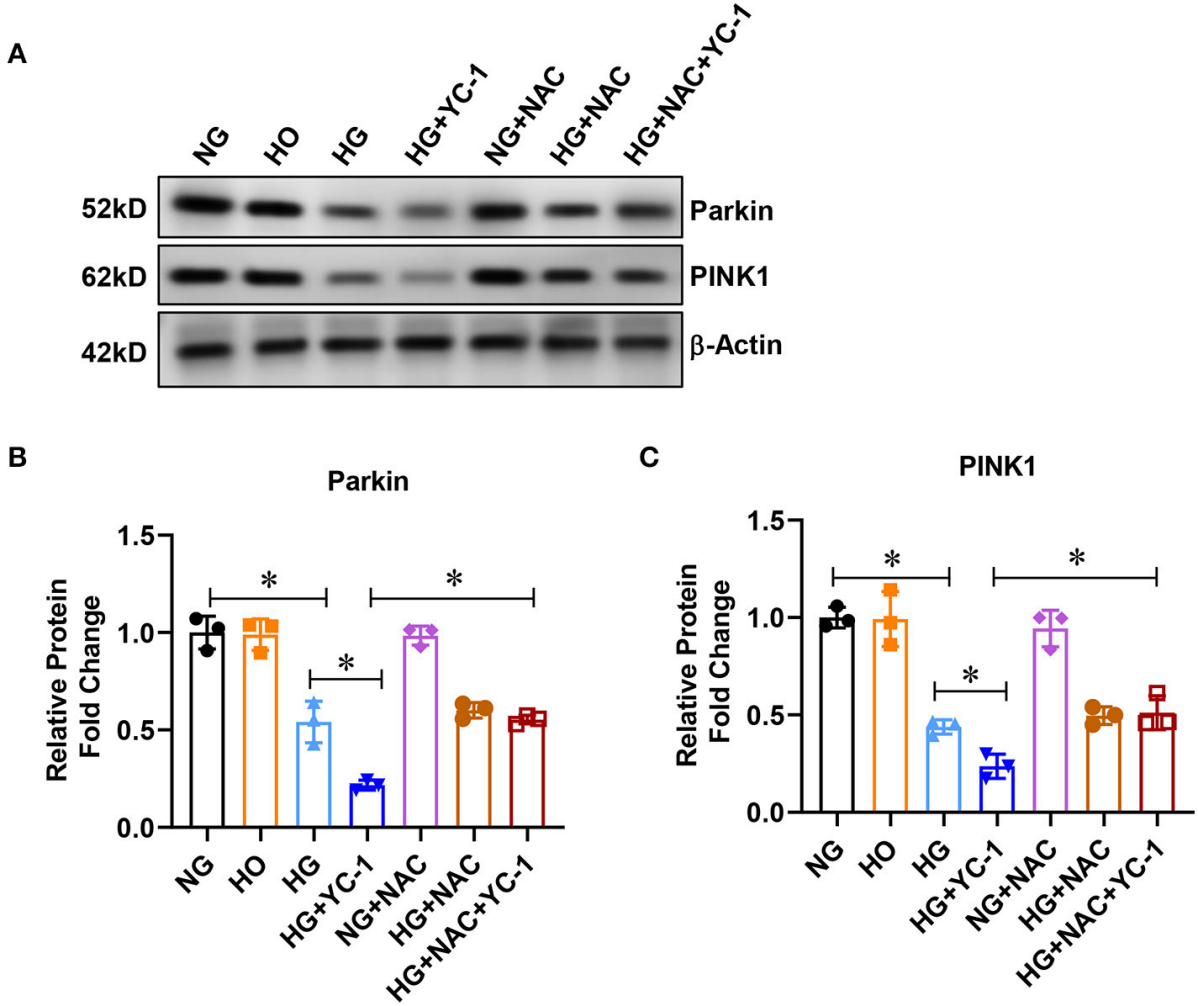
HIF-1α activated parkin/PINK1 pathway in HK-2cells subjected to HG exposure. **(A–C)** Expression of parkin and PINK1 was measured by Western blot in HK-2 cells treated with normal glucose (NG, 5.5 mM D-glucose), high-glucose (HG, 30mM D-glucose) for 24 h with or without 10 μM HIF-1α inhibitor YC- 1, and ROS scavenger NAC at a final concentration of 5 mm.*p < 0.05. vs. indicated group. HIF-1α, Hypoxia-Inducible Factor-1; Parkin PINK1, Parkin/PTEN Induced Putative Kinase1; YC-1, Lificiguat; ROS, Reactive Oxygen Species; NAC, N-Acetyl-L-Cysteine; HG, High Glucose.

Then, we examined whether parkin/PINK1 is a downstream regulatory factor in HIF1α-related mitophagy. The expression of parkin and PINK1 was significantly decreased in HK-2 cells exposed to the HG environment, and the decrease was further exacerbated by YC-1 (Figure 2A). The parkin and PINK1 expression were upregulated after adding the ROS scavenger NAC, indicating that high glucose levels induced inhibition of the parkin/PINK1 signaling pathway, which was reversed by the HIF-1α inhibitor (Figure 2A). These results showed that parkin/PINK1 is a downstream regulatory factor in an HIF-1α- related mitophagy.

### HIF-1α-Parkin/PINK1-Mediated Mitophagy Prevented Apoptosis and ROS Production in HK-2 Cells Subjected to HG Exposure

**Figure 3:**
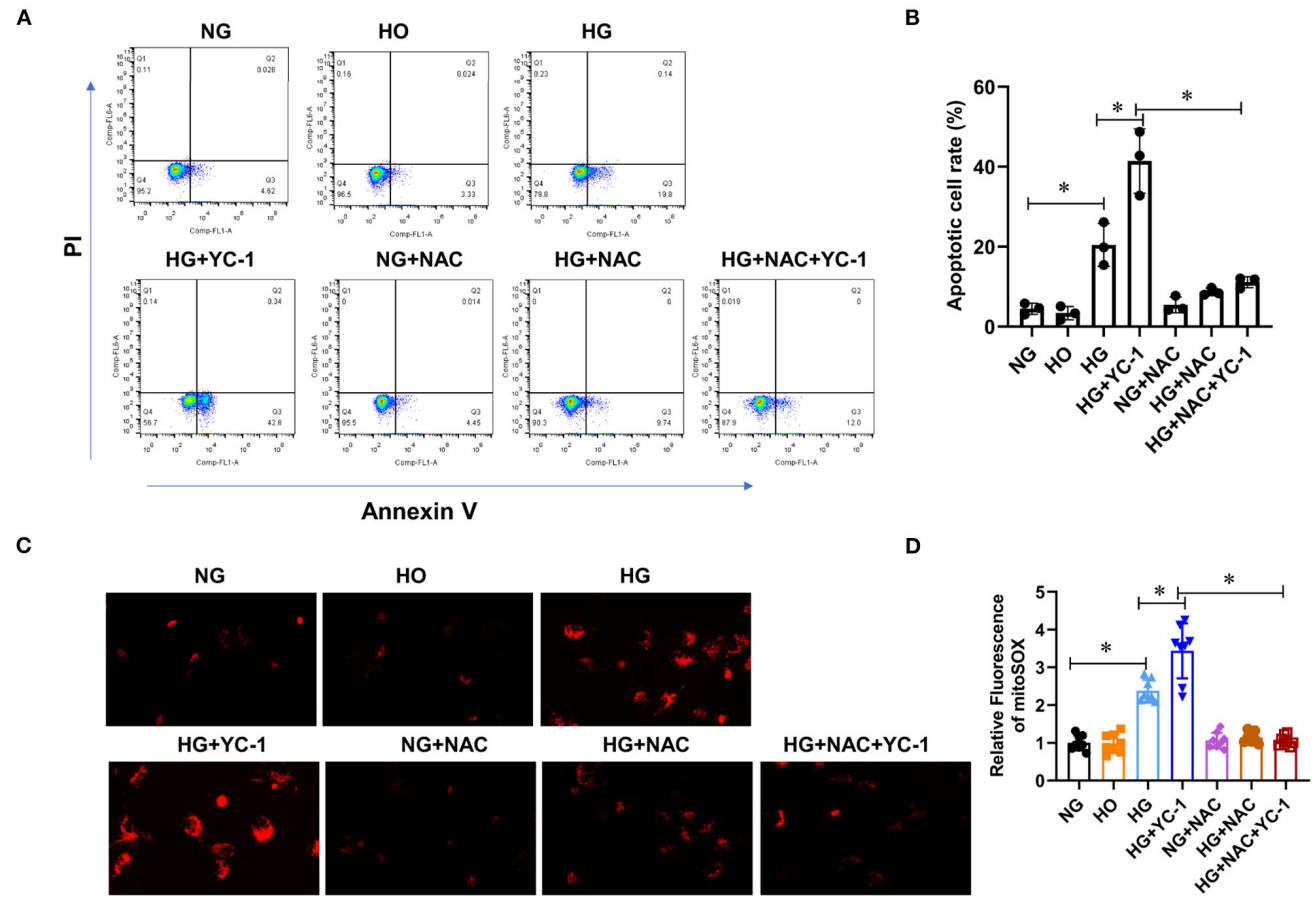
HIF-1α-Parkin/PINK1-mediated mitophagy prevented apoptosis and ROS production in HK-2 cells subjected to HG exposure. **(A,B)**. The level of apoptosis was quantified using CytoFLEX after annexin V and PI labeling. Apoptosis levels were significantly increased under high glucose conditions and further increased with YC- 1. This effect was reversed with NAC. *p < 0.05. vs. indicated group. **(C,D)**. Mitochondrial ROS production was measured using Image J software after MitoSOX staining. High glucose and inhibition of HIF-1α significantly increased intracellular ROS levels, while NAC reversed this trend. *p < 0.05. vs. indicated group. HIF-1α, Hypoxia-Inducible Factor-1; Parkin/PINK1, Parkin/PTEN Induced Putative Kinase1; YC-1, Lificiguat; ROS, Reactive Oxygen Species; NAC, N-Acetyl-L-Cysteine; HG, High Glucose.

Subsequently, we investigated whether a HIF1α-Parkin/PINK1-mediated mitophagy exerted a protective effect by decreasing apoptosis and ROS production in HK-2 cells exposed to the HG environment. We examined the effect of HIF- 1α on apoptosis under high glucose conditions by flow cytometry after labeling HK-2 cells with annexin V and PI, respectively. Annexin V-positive cells were significantly increased in HK-2 cells exposed to the HG environment, which was enhanced by YC-1 (Figures 3A,B). Similarly, ROS production is upregulated under HG conditions, and YC-1 further enhanced this phenomenon (Figure 3C). Interestingly, the stimulatory effect of YC-1 on apoptosis and ROS production was substantially inhibited by NAC (Figure 3C). This finding suggests that HIF-1α-Parkin/PINK1-mediated mitophagy might exert a protective effect by suppressing apoptosis and ROS production. Importantly, these results demonstrate that HIF-1α-Parkin/PINK1-mediated mitophagy protects renal tubular cells from apoptosis and ROS production when exposed to HG conditions.

### Inhibition of HIF-1α Significantly Promoted the Degree of HG-Induced Inflammation

**Figure 4:**
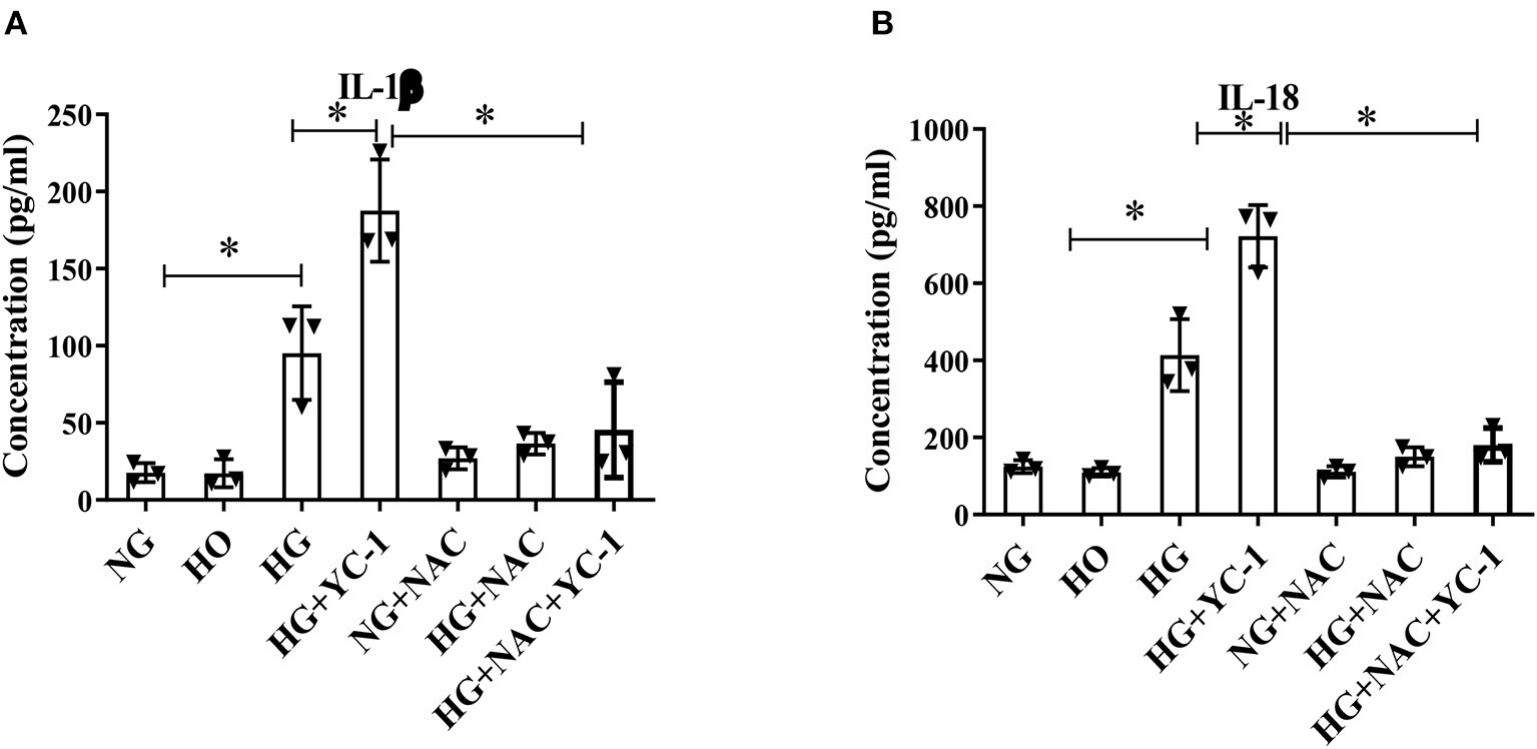
Inhibition of HIF-1α significantly promoted the degree of HG-induced inflammation. **(A,B)**. Inflammatory cytokines IL-1β and IL-18 in HK-2 cells subjected to HG exposure were quantitatively measured by ELISA. The cytokine levels were further increased with YC- 1 and reversed with NAC. *p < 0.05. vs. indicated group. HIF-1α, Hypoxia-Inducible Factor-1; HG, High Glucose; IL-β, Interleukin-1β; IL-18, Interleukin-18; YC-1, Lificiguat; NAC, N-Acetyl-L-Cysteine.

IL-1b is one of the main components of IL-1b and the main regulator of tissue inflammation. IL-18 is also a member of the proinflammatory cytokine family. ELISA was performed to quantify the levels of these two cytokines. The results showed that the inflammatory factors were significantly increased in the high glucose group, and inhibition of HIF-1α could substantially promote the release of these inflammatory factors (Figures 4A,B). We subsequently observed that NAC reversed the pro-inflammatory effect associated with the HIF-1α inhibitor. These results demonstrate that HIF-1α may reduce HG-mediated inflammation in HK-2 cells.

### HIF-1α Inhibited Epithelial-Mesenchymal Transition in HK-2 Cells Subjected to HG Exposure

**Figure 5:**
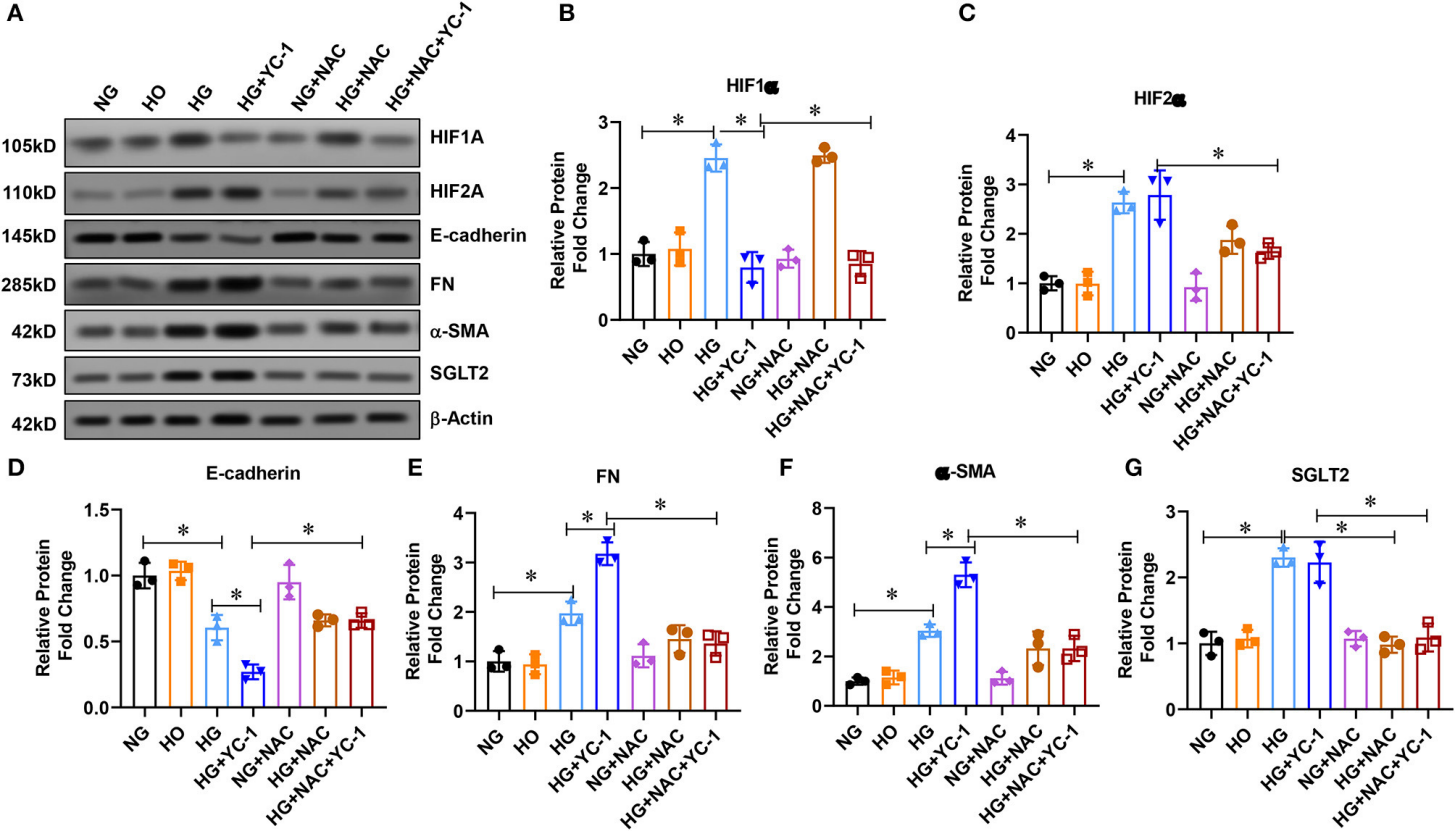
HIF-1 α reduced epithelial-mesenchymal transition (EMT) in HK-2 cells subjected to HG exposure. **(A–G)**. Western blot analysis revealed that FN and a-SMA were significantly increased, while E-cadherin expression was significantly decreased in HK-2 cells subjected to HG conditions. EMT, Epithelial-Mesenchymal Transition; HG, High Glucose; FN, Fibronectin; A-SMA, Alpha-Smooth Muscle Actin.

To investigate the role of HIF-1α on the epithelial-mesenchymal transition process in HK-2 cells exposed to HG environment, we extracted the total protein of HK-2 cells after different treatments and performed Western blot experiments. The results pointed out that the expression of FN and a-SMA were significantly up-regulated, and E-cadherin expression was decreased under high glucose treatment (Figure 5A), suggesting that HK-2 cells underwent epithelial-mesenchymal transition EMT. The expression of FN and α-SMA was further increased, and E-cadherin expression was further decreased after the addition of YC-1 (a specific inhibitor of HIF-1α) (Figure 5A), indicating further enhancement of EMT in HK-2 cells. We further found that NAC (an ROS scavenger) significantly reversed the pro-EMT effect of the HIF-1α inhibitor in a high glucose environment. These results suggest that HIF-1α inhibited epithelial-mesenchymal transition (EMT) in HK-2 cells exposed to HG environment.

## Discussion

Diabetic renal tubular lesions are widely involved in the development of diabetic kidney disease (DKD). It has been established that during the pathogenesis of DKD, renal tubular injury leads to primary renal injury (23, 24), which is mirrored by an increase in renal tubular injury markers before microalbuminuria. However, the mechanism underlying this phenomenon remains largely unclear.

As one of the important cell organelles, mitochondria provide most of the energy required by the body and are an important source of ROS. Mitochondria are highly dynamic organelles. Its function maintenance is one of the foundations to ensure the normal operation of cells. Mitochondrial homeostasis depends on the balance between mitophagy and mitochondrial biogenesis (25). This balance is crucial to repair damaged mitochondria and ensure normal mitochondrial function (26–28). Mitophagy has been shown to play an indispensable protective role in different disease models of DN and ischemia-reperfusion injury (29). In a mouse model of acute renal injury (AKI) induced by cisplatin, changes in mitochondrial autophagy significantly exacerbated AKI (30). Moreover, inhibiting autophagy in proximal convoluted tubules with chloroquine or knocking out autophagy gene-related 7 (Atg7) molecules led to tissue damage, renal insufficiency, and apoptosis (31). In contrast, rapamycin has been found to activate the mitochondrial autophagy pathway and to reduce cisplatin-induced AKI renal tubular injury. Interestingly, one study showed that an HIF-1α- BNIP 3 mediated mitochondrial autophagy exerts a protective effect against AKI by decreasing renal tubular cell apoptosis, suggesting that HIF-1α plays an important role in hypoxia-induced mitophagy (13, 32). Consistent with the literature, our study indicated that mitophagy plays an important role in DN. Interestingly, recent studies have found that mitophagy is correlated to renal tubular disease in DN. The present study found that HIF-1α alleviates HG-mediated renal tubular cell injury by promoting Parkin/PINK1-mediated mitophagy.

Although HIF-1α plays different roles in many cellular processes [for instance, autophagic degradation, cellular survival, energy homeostasis, and angiogenesis (16)], little is known on the relationship between HIF-1α and autophagy, especially mitophagy. The present study indicated that HIF-1α alleviated high-glucose-induced renal tubular cell injury (33), a phenomenon that was accompanied by a decreased cell apoptosis. The HIF-1α exerted a protective effect in regulating mitophagy *via* promoting the Parkin/PINK1 signaling pathway (13). Moreover, a decreased ROS synthesis mediated by HIF-1α may account for the enhanced mitophagic activity (34). Taken together, these results exhibit a new mechanism by which HIF-1α protects against hyperglycemia-induced oxidative injury and enhances mitophagic activity, at least partially *via* Parkin/PINK1 signaling pathway in tubular cells (35).

The PINK1/parkin is the most studied mitophagic pathway (15, 36). The PINK1 leads to the degradation of mitochondrial protein *via* contact with the surface of the depolarized mitochondrion and inducing Parkin translocation. Zhan et al. observed that a high-glucose environment induced a decreased PINK1/Parkin expression, LC3, and mitophagy in renal tubular epithelial cells in Streptozotocin (STZ)-induced DN mice, which caused mitochondrial ROS (mROS) overproduction, mitochondrial fission, and apoptosis (37). Consistently, Xiao et al. found that db/db mice exhibited reduced PINK1/Parkin and LC3 expression. In the meantime, p62 accumulation in renal tubular epithelial cells results in mitophagy disorders, mitochondrial dysfunction, and apoptosis (38). Nevertheless, the upstream molecular mechanisms of mitophagy in DN have been understudied, warranting further studies.

In 1971, Loschek first demonstrated that the mitochondrial respiratory chain could produce oxygen free radicals, leading to ROS synthesis. The hyperglycemic state in diabetes can lead to an enlarged mitochondrial inner membrane potential difference and formation of an inner membrane hyperpolarossification. Electrons transported from coenzyme Q to cytochromes can reduce oxygen molecules to water and, thus, producing large amounts of ROS. In addition, the inner membrane of mitochondria is highly concentrated in unsaturated fatty acids and hence vulnerable to ROS. Accordingly, the mitochondria are the primary site of ROS synthesis *in vivo* and are conversely the most sensitive to oxidative damage by ROS (39). We observed that the NAC increased mitophagy and reduced ROS levels under HG conditions, and this phenomenon was exacerbated by inhibition of HIF-1α. The results of this study provide compelling evidence that HG exposure leads to an increased mitophagic activity and the release of large quantities of ROS.

Overall, this study demonstrates that the HIF-1α alleviates high-glucose-mediated renal tubular cell injury by promoting Parkin/PINK1-mediated mitophagy. These findings provide the basis for future studies on the pathogenesis of DN and provide important information for disease prevention and treatment.

## Data Availability Statement

The original contributions presented in the study are included in the article/Supplementary Material, further inquiries can be directed to the corresponding author/s.

## Author Contributions

LY, LT, and YW designed, performed, and analyzed the experiments. LY wrote and revised the manuscript. LY, YW, ZY, and YG carried out the data collection, data analysis, and revised the manuscript. LT revised the manuscript and applied for funding support. LY and LT confirm the authenticity of the raw data. All authors contributed to the article and approved the submitted version.

## Funding

The present study was supported by the National Natural Science Foundation of China (grant no. U1904134) and Henan Province Young and Middle-aged Health Science and Technology Innovative Talents (Leader) Project (grant no. YXKC2020014).

## Conflict of Interest

The authors declare that the research was conducted in the absence of any commercial or financial relationships that could be construed as a potential conflict of interest.

## Publisher's Note

All claims expressed in this article are solely those of the authors and do not necessarily represent those of their affiliated organizations, or those of the publisher, the editors and the reviewers. Any product that may be evaluated in this article, or claim that may be made by its manufacturer, is not guaranteed or endorsed by the publisher.
