# Corrigendum: HIF-1α alleviates high-glucose-induced renal tubular cell injury by promoting Parkin/PINK1-mediated mitophagy

**DOI:** 10.3389/fmed.2025.1571785

**Published:** 2025-03-10

**Authors:** Lu Yu, Yulin Wang, Yan Hong Guo, Liuwei Wang, Zijun Yang, Zi Han Zhai, Lin Tang

**Affiliations:** ^1^First Affiliated Hospital of Zhengzhou University, Zhengzhou, China; ^2^College of Public Health, Zhengzhou University, Zhengzhou, China

**Keywords:** HIF-1α, mitophagy, diabetic nephropathy, inflammation, ROS, apoptosis

In the published article, there was an error in Figure 1D as published. Upon reviewing our submission, we realized that the image for the HO treatment group in Figure 1D is not correct. The corrected Figure 1D and its caption appear below.

**Figure 1:**
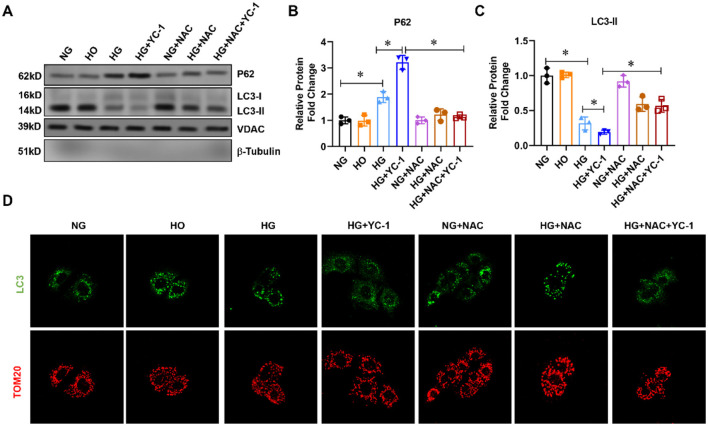
Effect of HIF-1α on mitophagy-related proteins in HK-2cells subjected to HG exposure. **(A–C)** The expression of p62 and LC3-II was assessed by Western blot in HK-2 cells treated with normal glucose, high-glucose for 24 h with or without 10 μM HIF-1α inhibitor YC-1, and reactive oxygen species (ROS) scavenger N-Acetyl-L-Cysteine (NAC) at a final concentration of 5 mm. ^*^p < 0.05. vs. indicated group. **(D)** Mitophagy was assessed using a fluorescence confocal microscope. HG, High Glucose; LC3-II, Microtubule-Associated-Proteinlight-Chain-3 II; HIF-1α, Hypoxia-Inducible Factor-1; YC-1, Lificiguat; ROS, Reactive Oxygen Species; NAC, N-Acetyl-L-Cysteine.

The authors apologize for this error and state that this does not change the scientific conclusions of the article in any way. The original article has been updated.

