# Supplementary figures and images for: HIF-1α Alleviates High-Glucose-Induced Renal Tubular Cell Injury by Promoting Parkin/PINK1-Mediated Mitophagy
Source: Front Med (Lausanne). 2022 Feb 3;8:803874. doi: 10.3389/fmed.2021.803874 (PMC8850720; doi:10.3389/fmed.2021.803874)

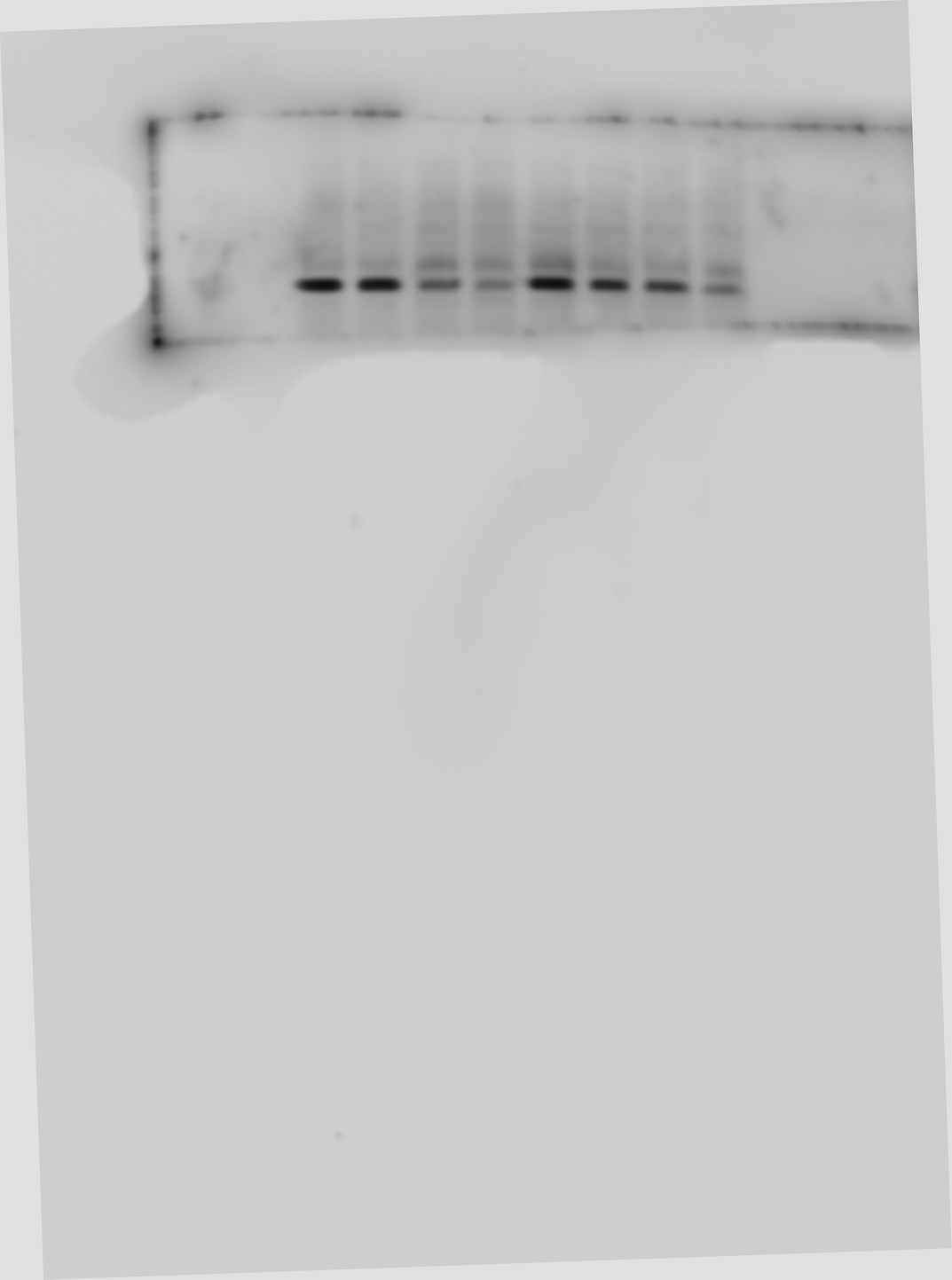

Supplement: Supplementary file 1 [file Data_Sheet_1.ZIP › 1-wb/Ch-LC3/Ch-LC3.tif]

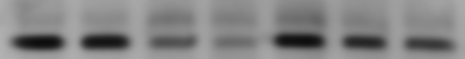

Supplement: Supplementary file 1 [file Data_Sheet_1.ZIP › 1-wb/Ch-LC3/LC3-II.tif]

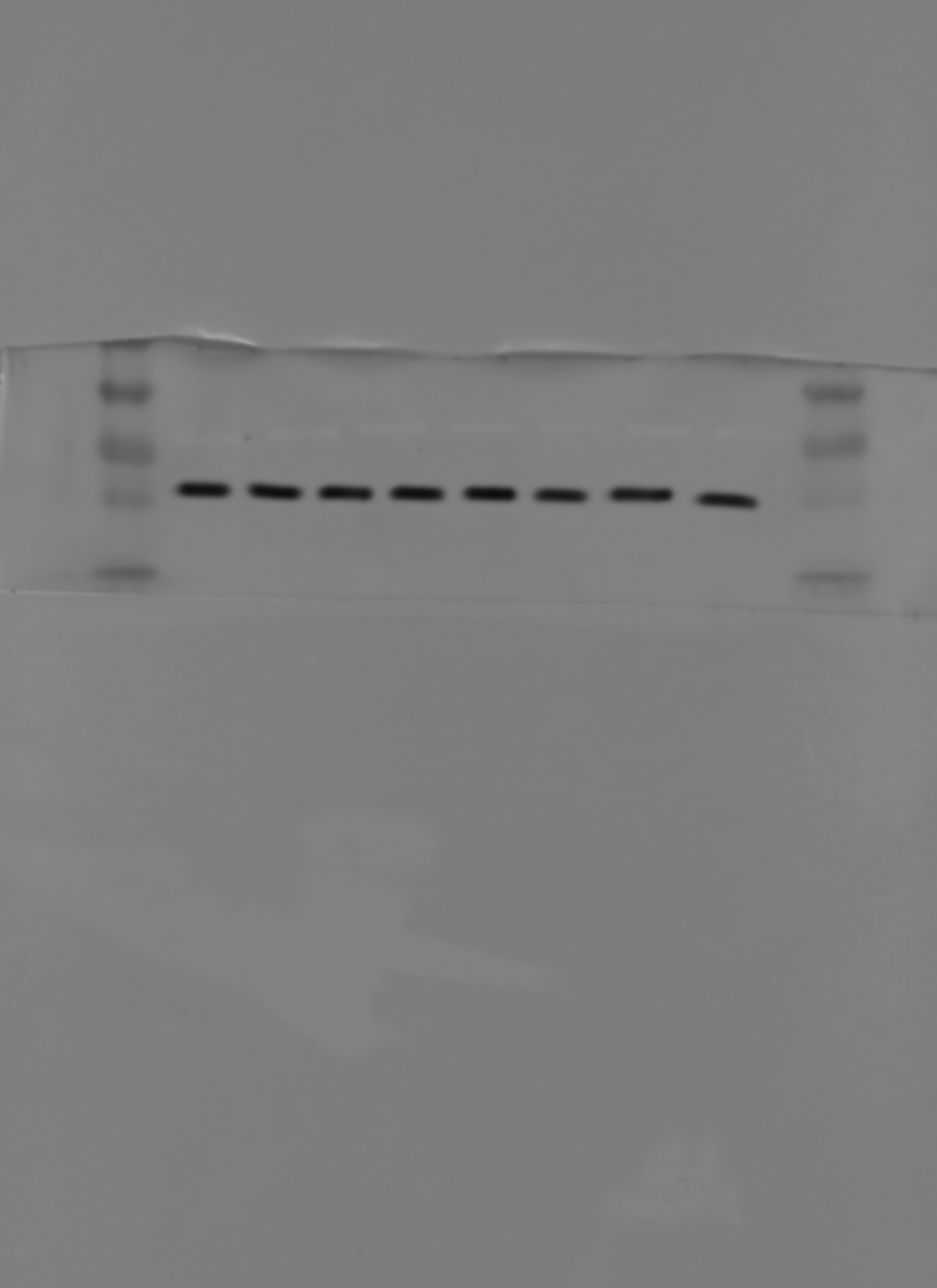

Supplement: Supplementary file 1 [file Data_Sheet_1.ZIP › 1-wb/Ch-Marker-VDAC-2.tif]

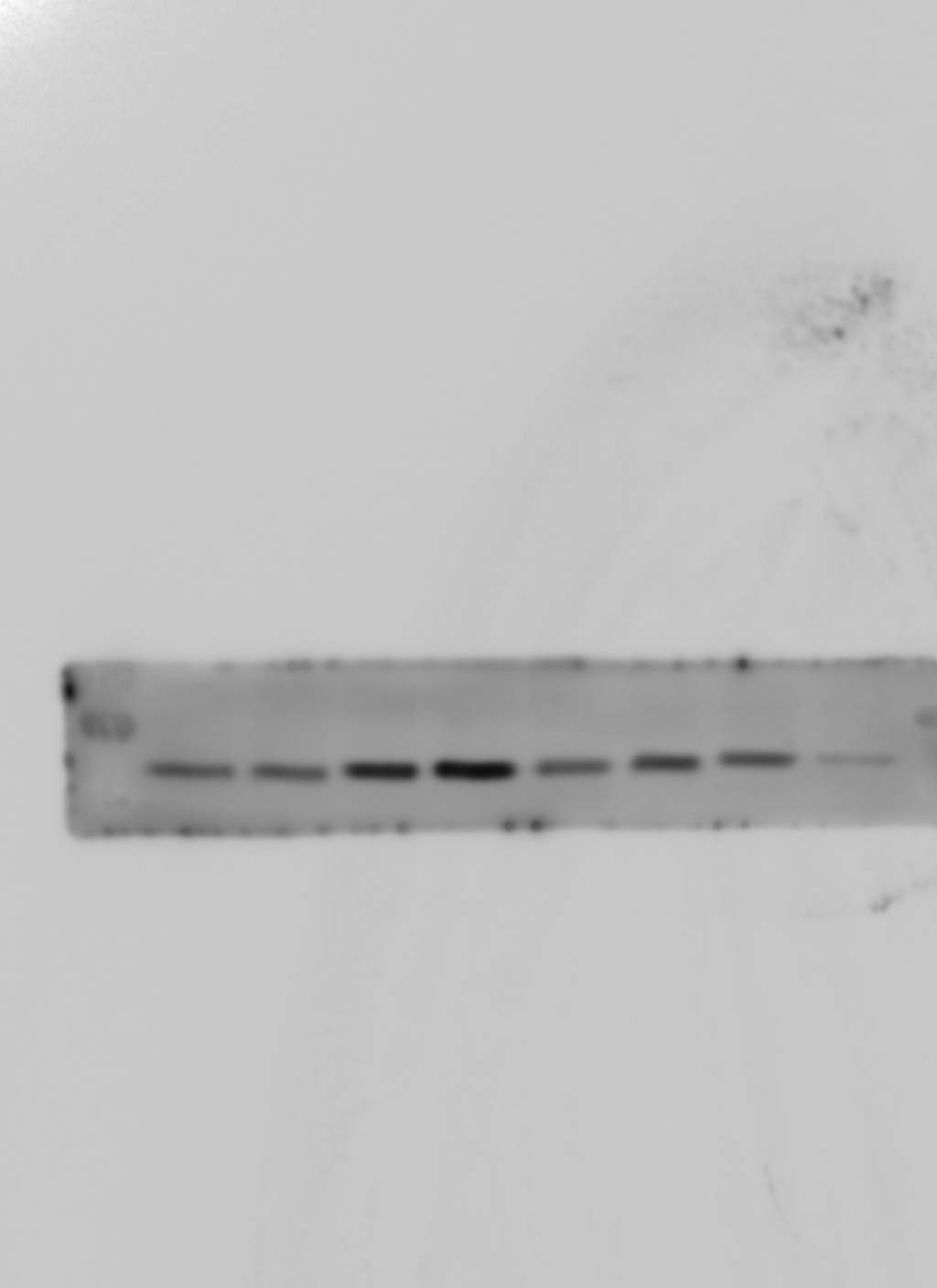

Supplement: Supplementary file 1 [file Data_Sheet_1.ZIP › 1-wb/Ch-P62/Ch-P62.tif]

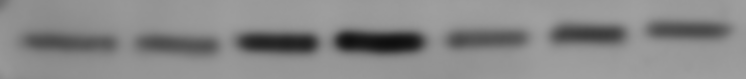

Supplement: Supplementary file 1 [file Data_Sheet_1.ZIP › 1-wb/Ch-P62/P62.tif]

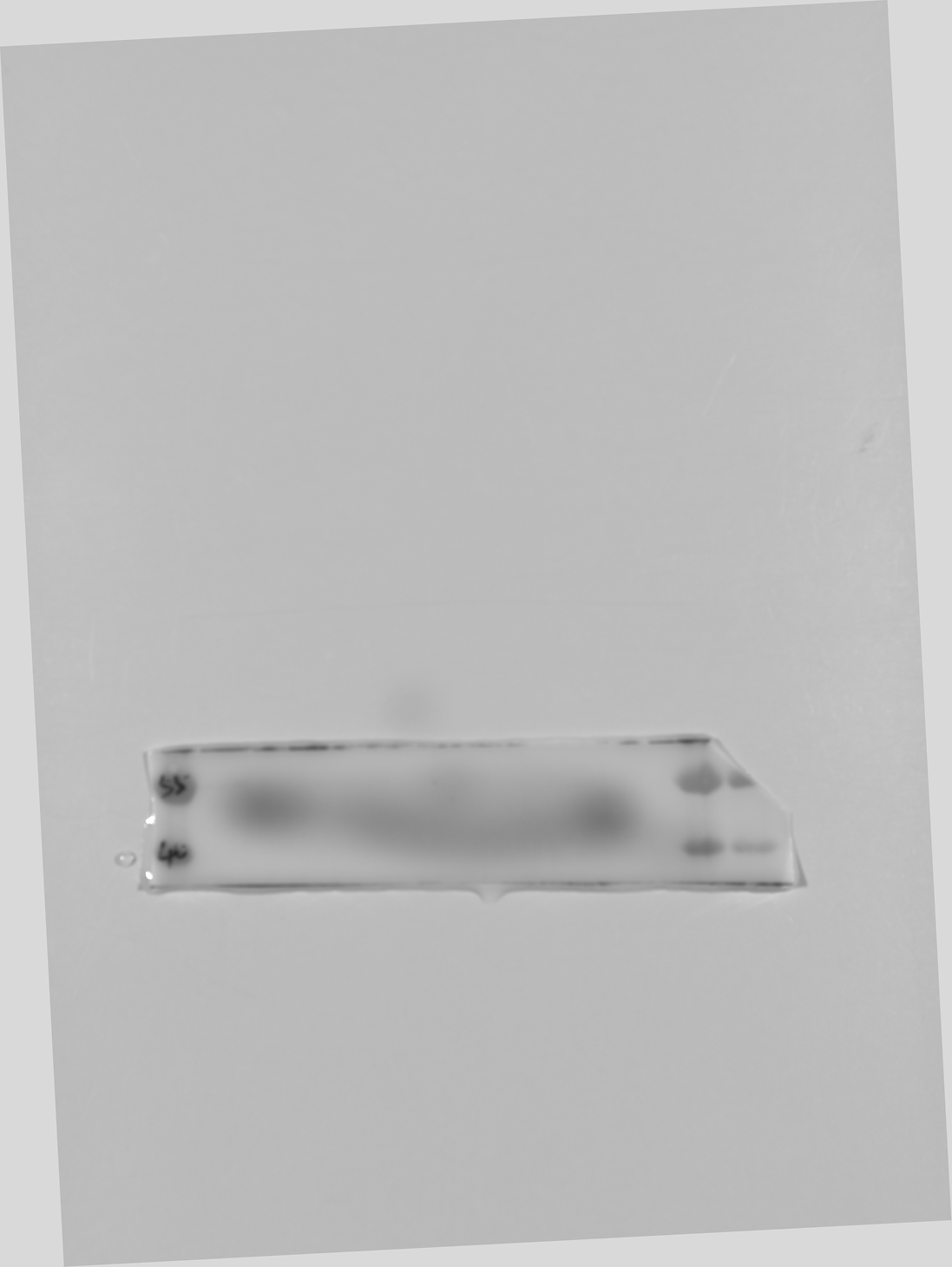

Supplement: Supplementary file 1 [file Data_Sheet_1.ZIP › 1-wb/Ch-Tubulin/Ch-Marker-b-Tubulin.tif]

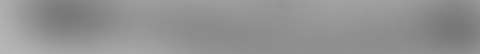

Supplement: Supplementary file 1 [file Data_Sheet_1.ZIP › 1-wb/Ch-Tubulin/Tubulin.tif]

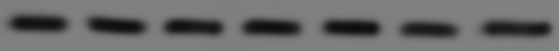

Supplement: Supplementary file 1 [file Data_Sheet_1.ZIP › 1-wb/VDAC.tif]

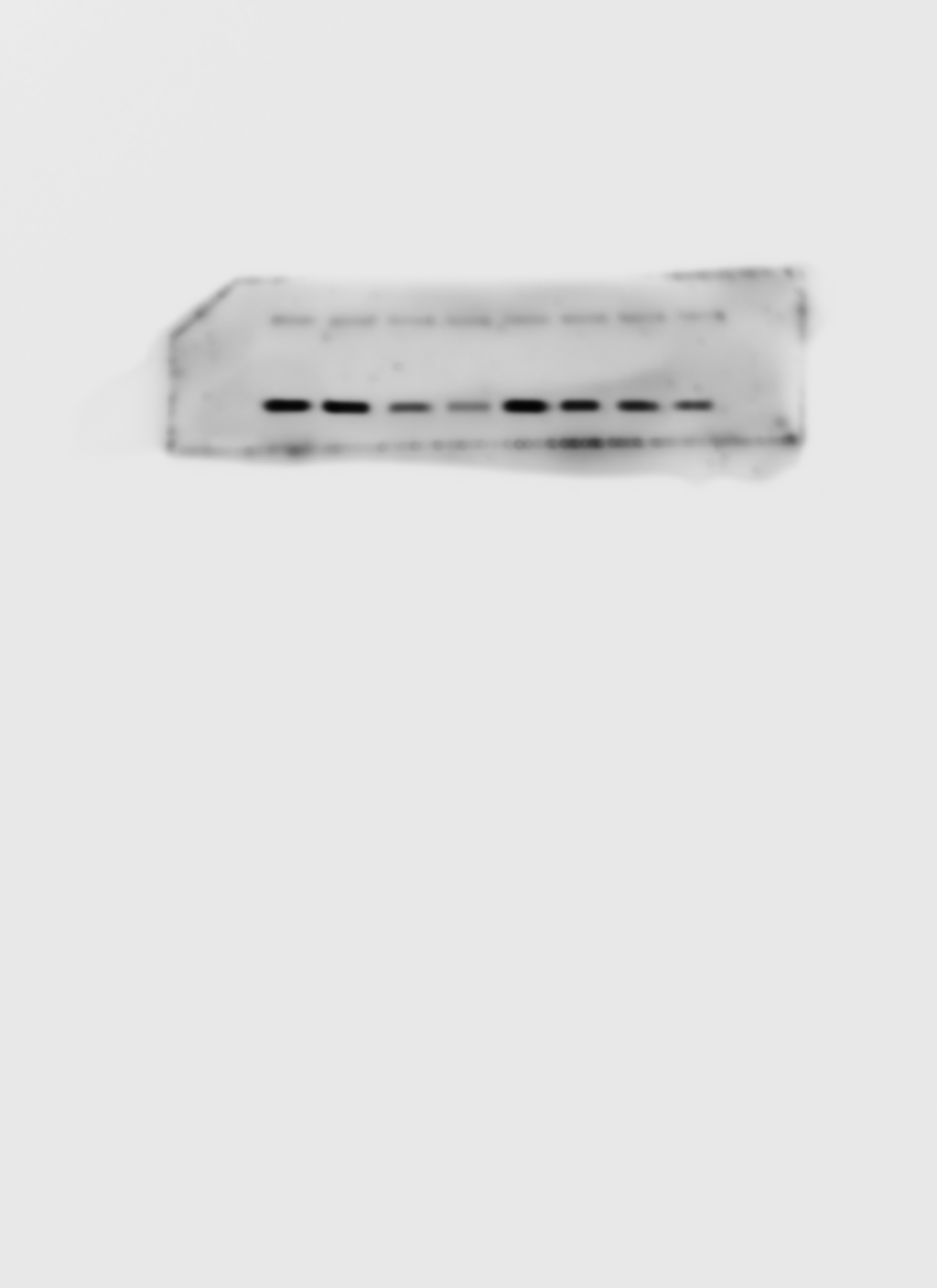

Supplement: Supplementary file 1 [file Data_Sheet_1.ZIP › 1-wb/parkin-pink1 pathway/Ch-PINK1/Ch-PINK1.tif]

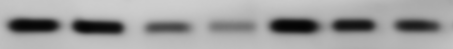

Supplement: Supplementary file 1 [file Data_Sheet_1.ZIP › 1-wb/parkin-pink1 pathway/Ch-PINK1/PINK1.tif]

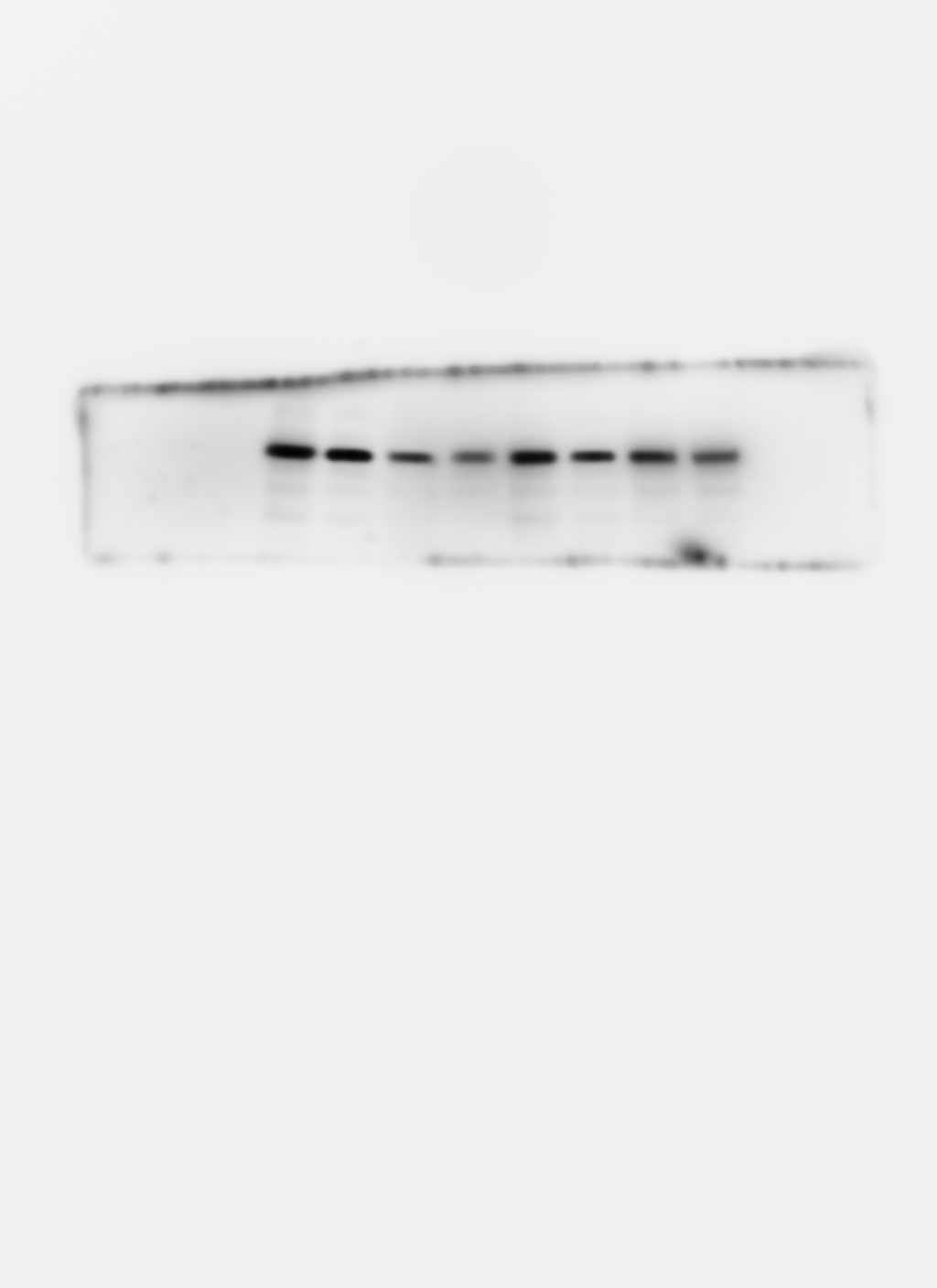

Supplement: Supplementary file 1 [file Data_Sheet_1.ZIP › 1-wb/parkin-pink1 pathway/Ch-Parkin/Ch-Parkin.tif]

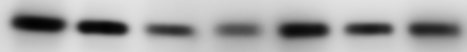

Supplement: Supplementary file 1 [file Data_Sheet_1.ZIP › 1-wb/parkin-pink1 pathway/Ch-Parkin/parkin.tif]

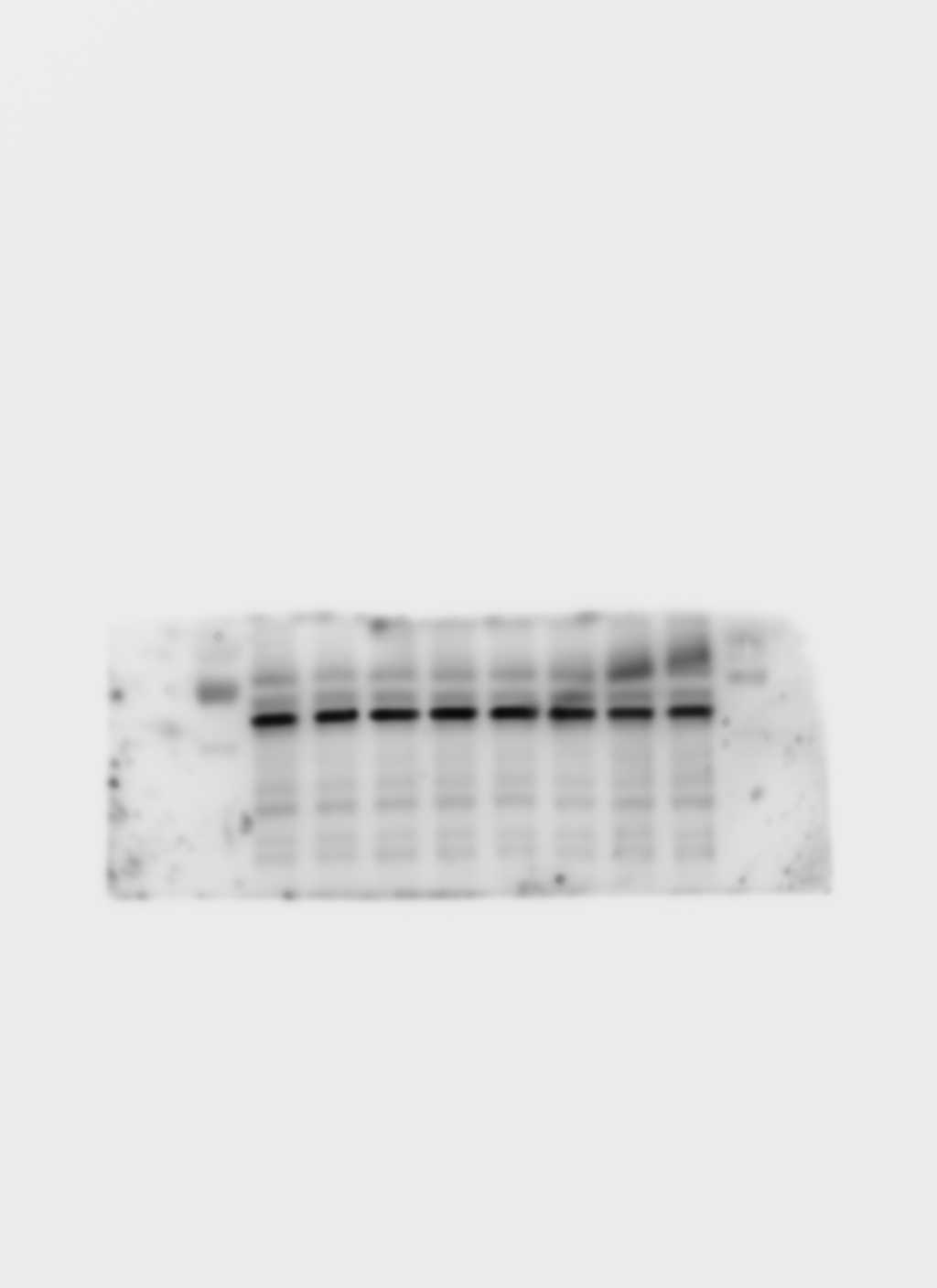

Supplement: Supplementary file 1 [file Data_Sheet_1.ZIP › 1-wb/parkin-pink1 pathway/Ch-b-Actin/Ch-b-Actin.tif]

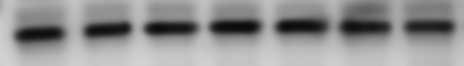

Supplement: Supplementary file 1 [file Data_Sheet_1.ZIP › 1-wb/parkin-pink1 pathway/Ch-b-Actin/b-Actin.tif]

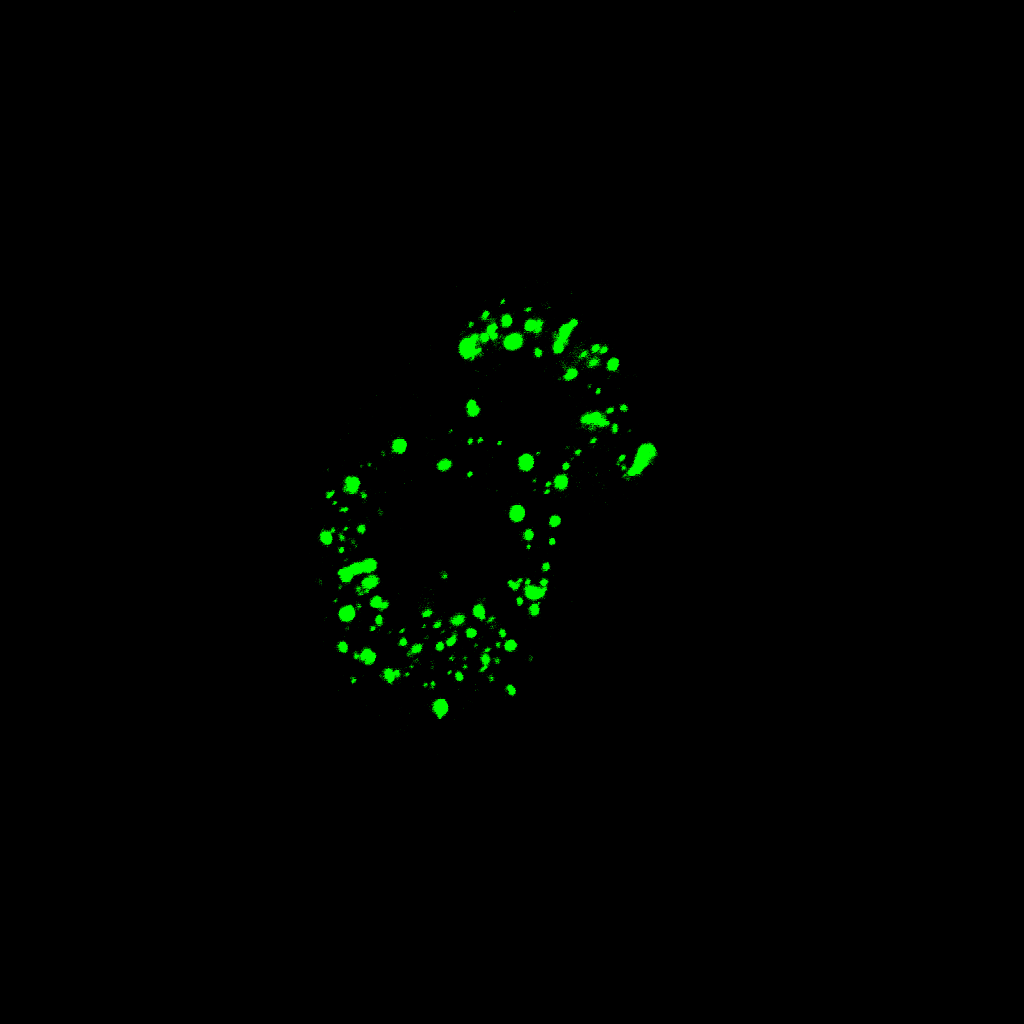

Supplement: Supplementary file 2 [file Data_Sheet_2.ZIP › 2-Mitophagy/Image35-G.tif]

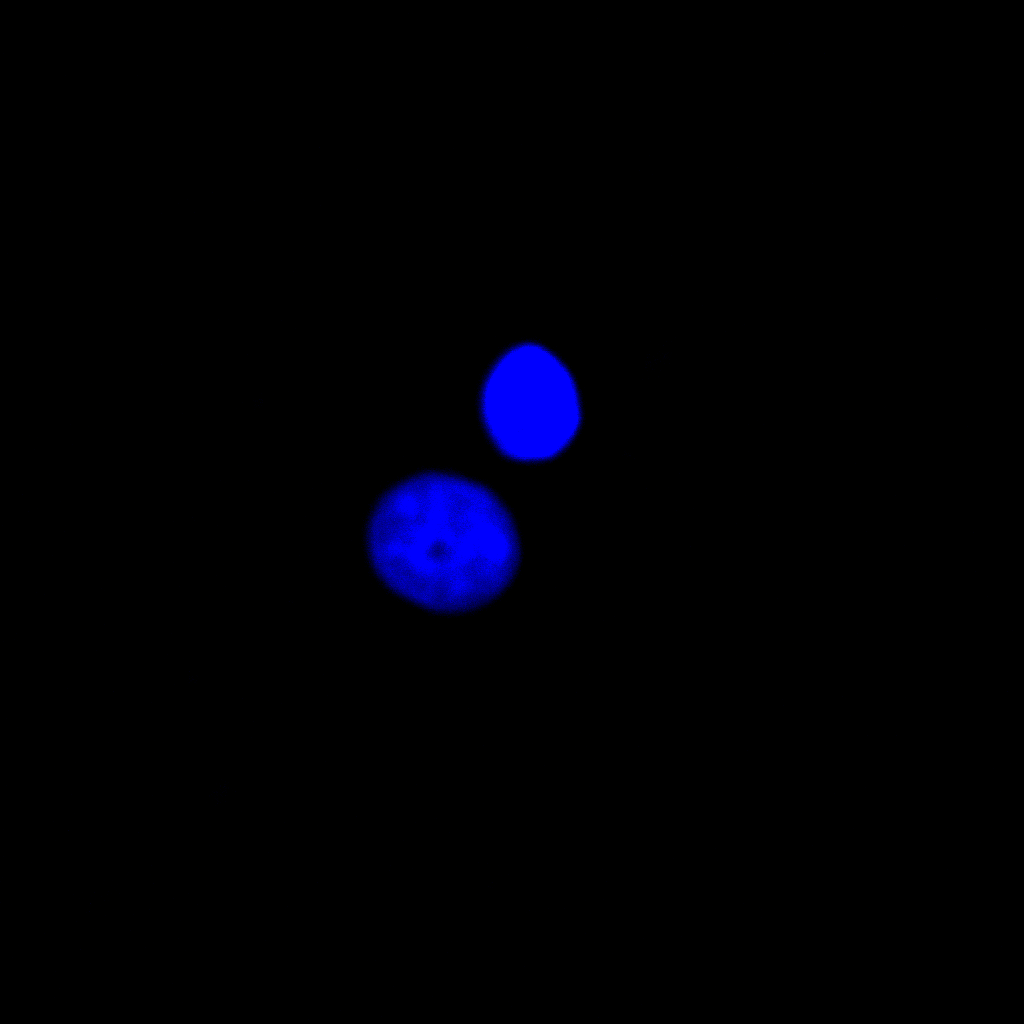

Supplement: Supplementary file 2 [file Data_Sheet_2.ZIP › 2-Mitophagy/Image35-H.tif]

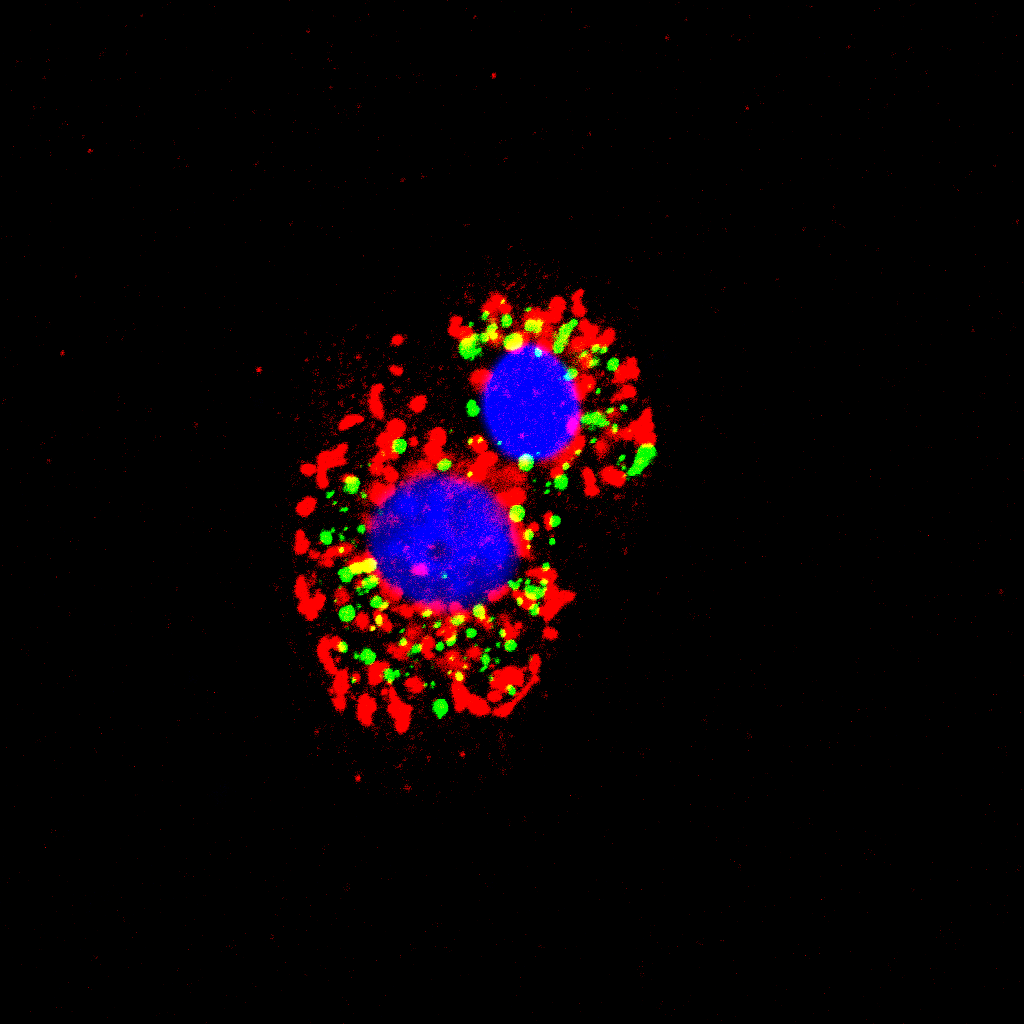

Supplement: Supplementary file 2 [file Data_Sheet_2.ZIP › 2-Mitophagy/Image35-HG+NAC.tif]

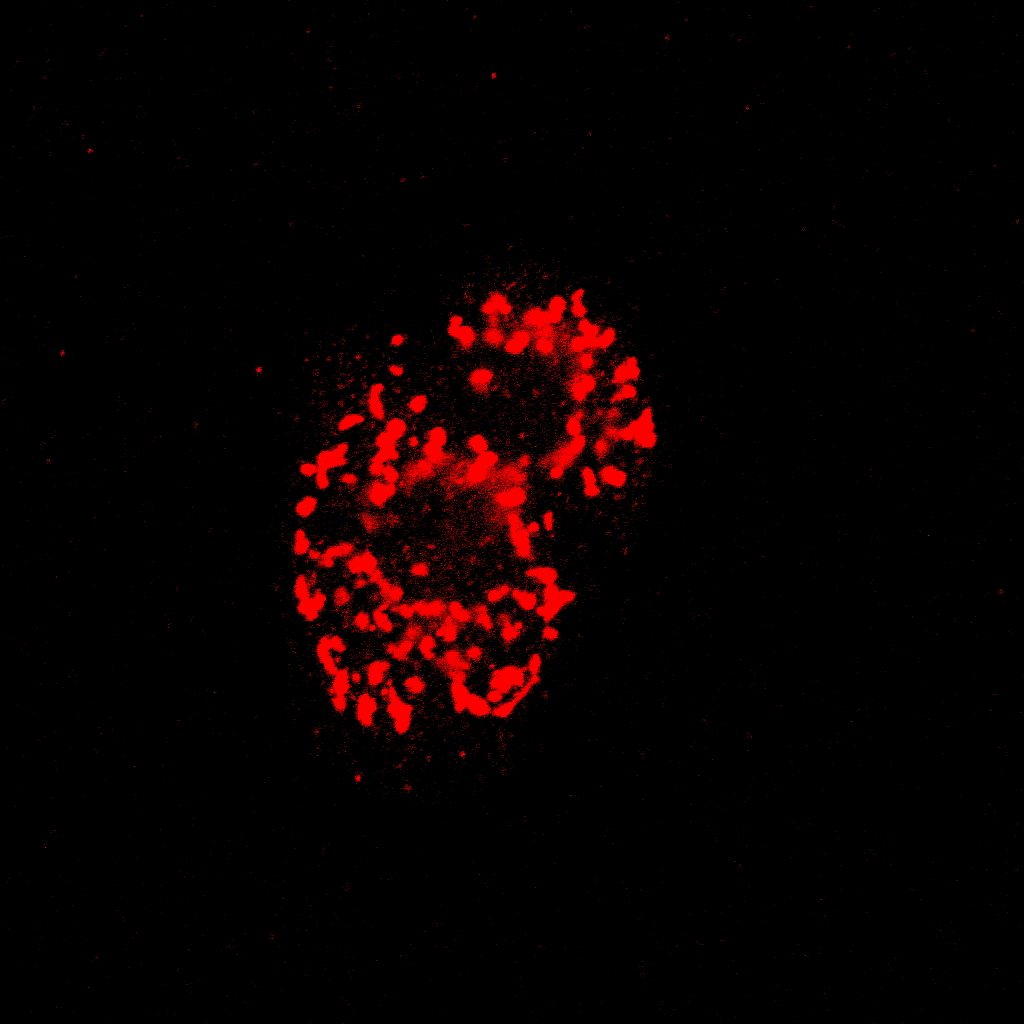

Supplement: Supplementary file 2 [file Data_Sheet_2.ZIP › 2-Mitophagy/Image35-R.tif]

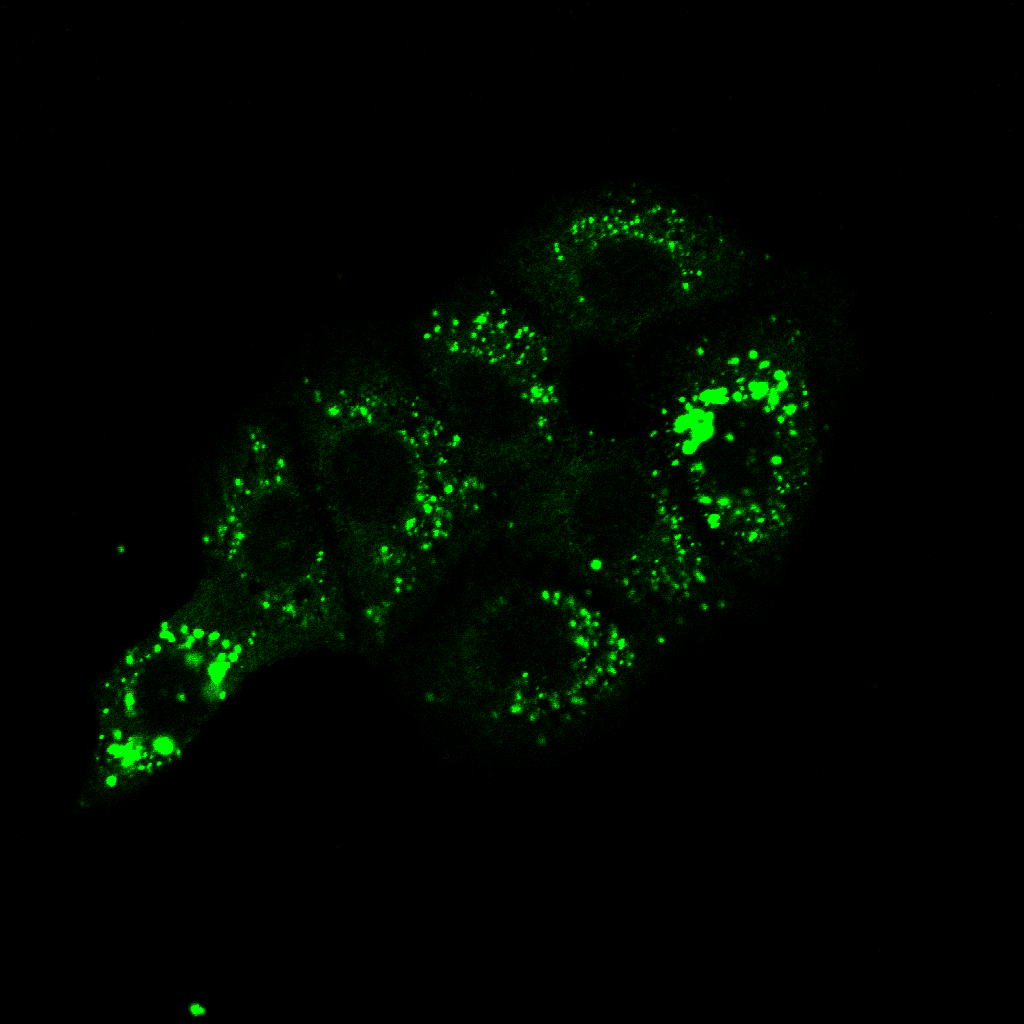

Supplement: Supplementary file 2 [file Data_Sheet_2.ZIP › 2-Mitophagy/Image36-G.tif]

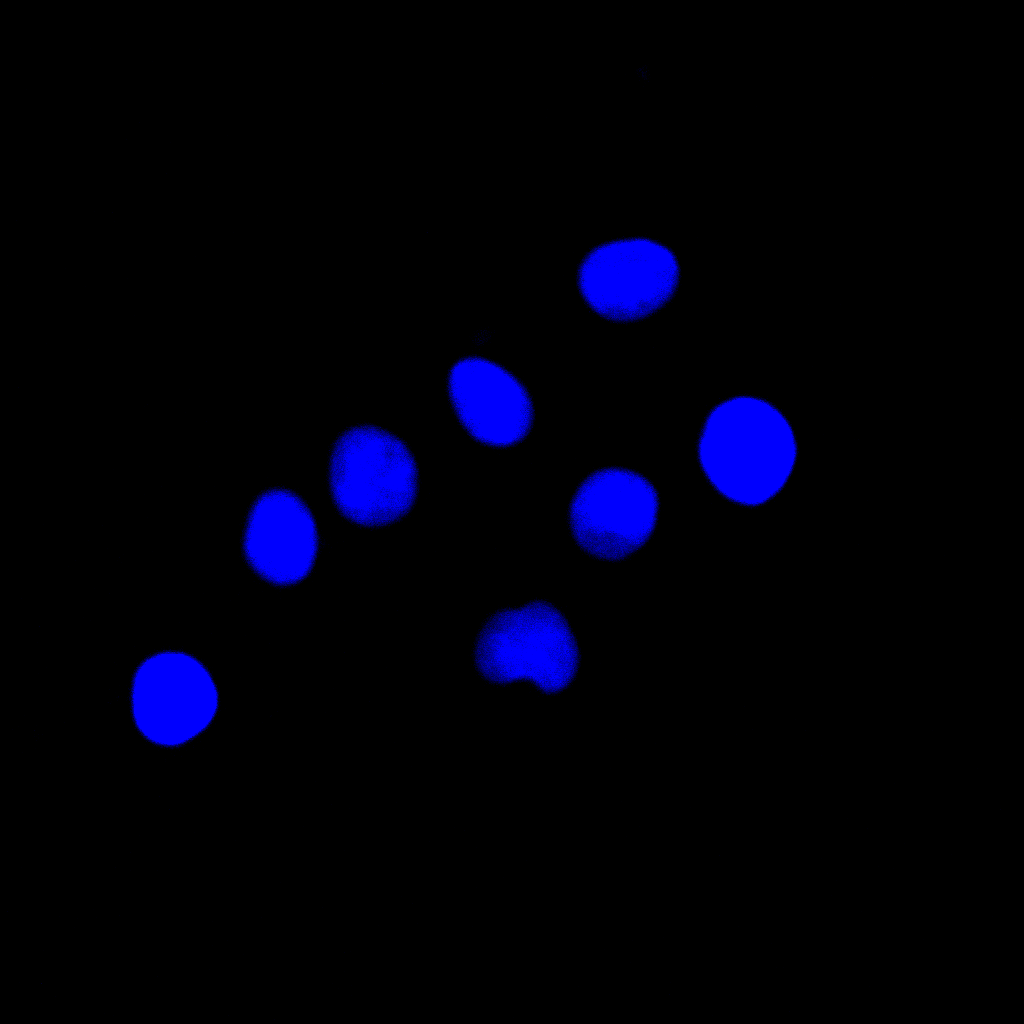

Supplement: Supplementary file 2 [file Data_Sheet_2.ZIP › 2-Mitophagy/Image36-H.tif]

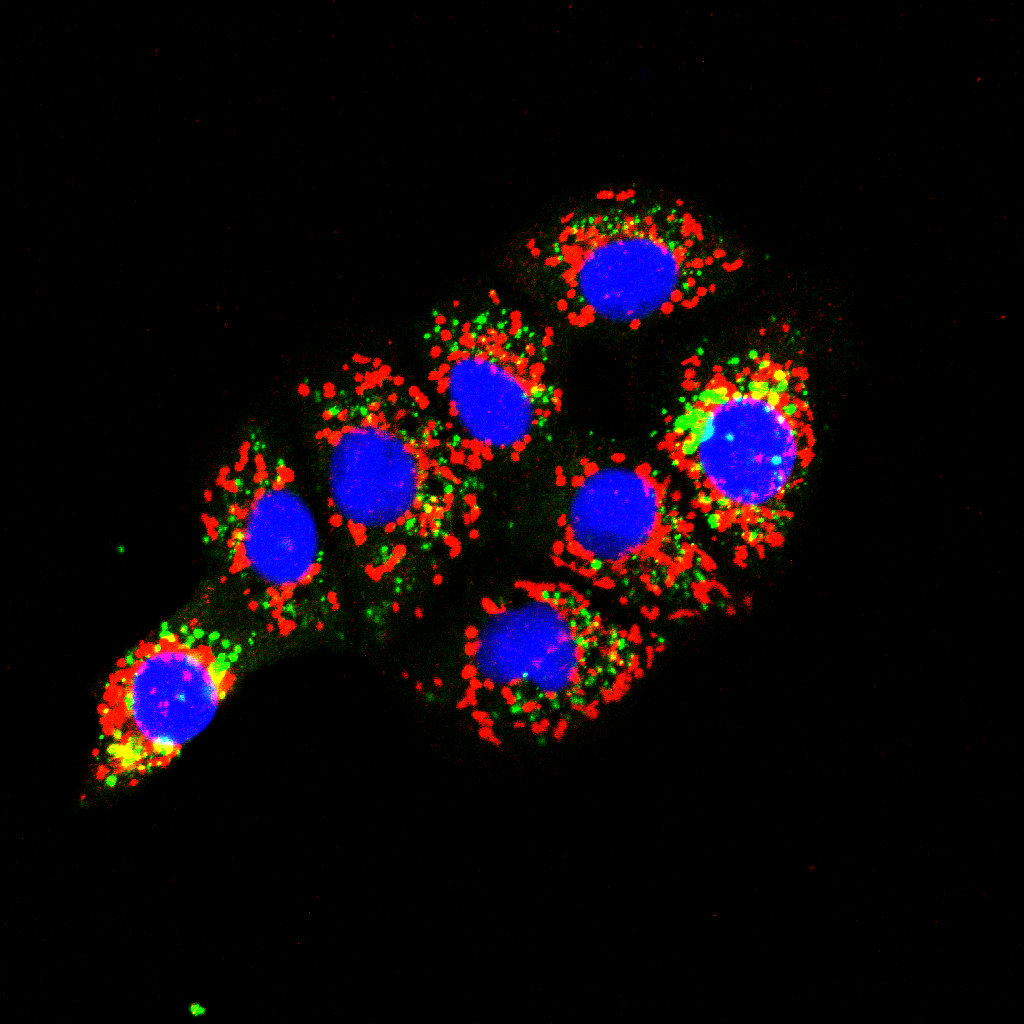

Supplement: Supplementary file 2 [file Data_Sheet_2.ZIP › 2-Mitophagy/Image36-NG+NAC.tif]

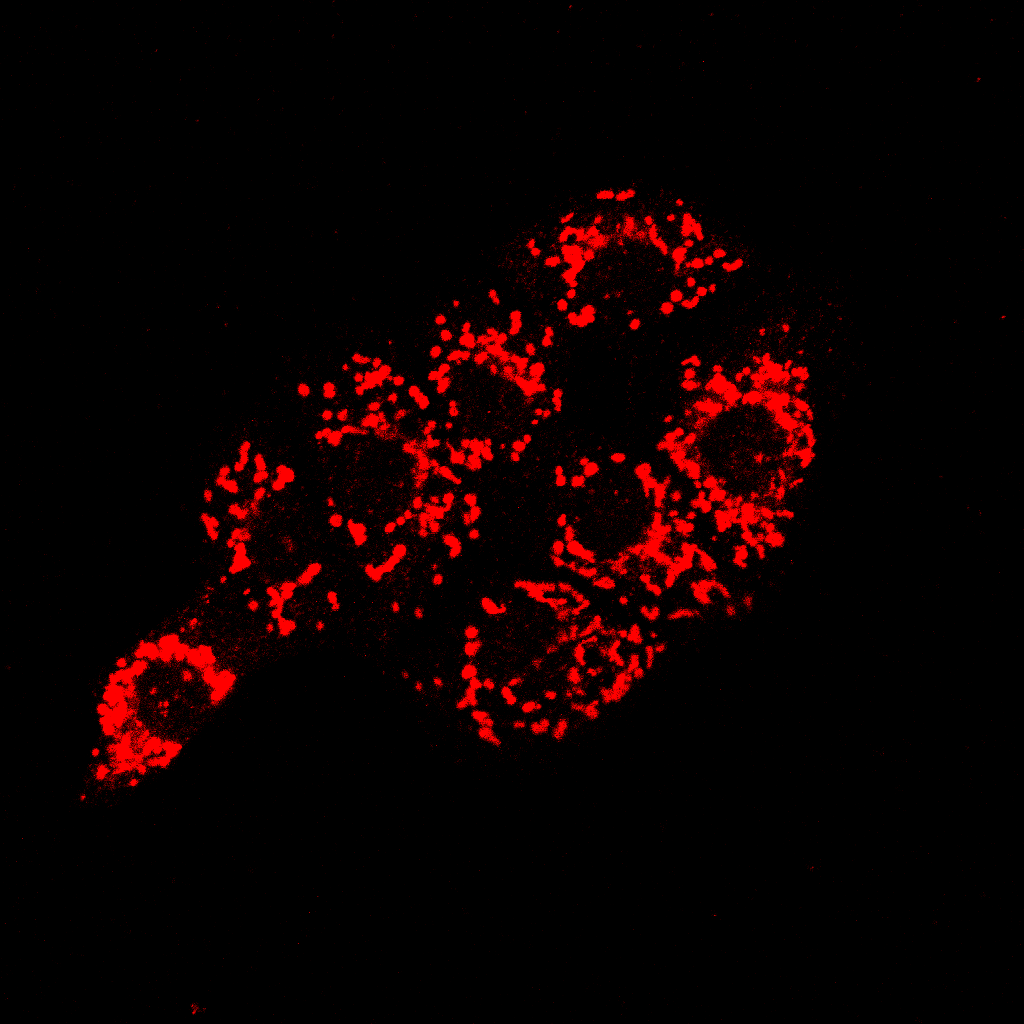

Supplement: Supplementary file 2 [file Data_Sheet_2.ZIP › 2-Mitophagy/Image36-R.tif]

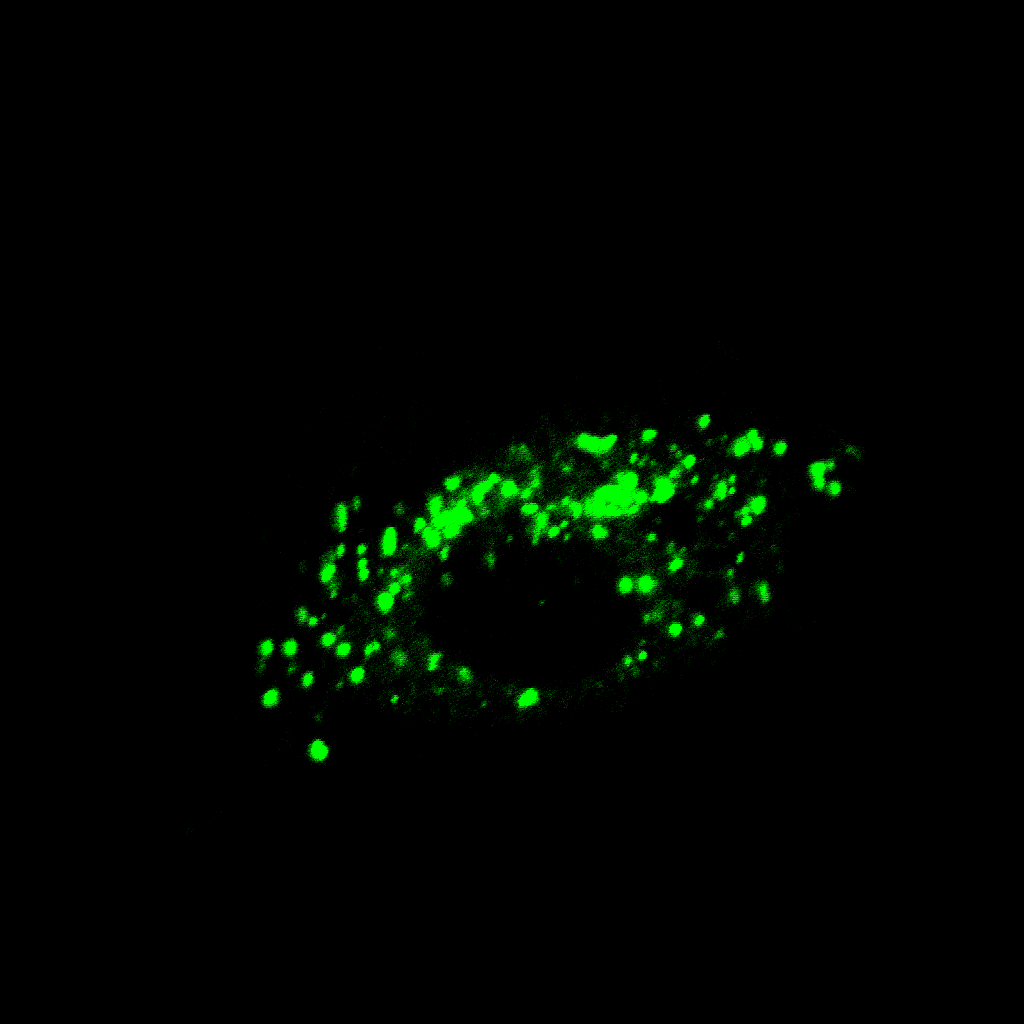

Supplement: Supplementary file 2 [file Data_Sheet_2.ZIP › 2-Mitophagy/Image37-G.tif]

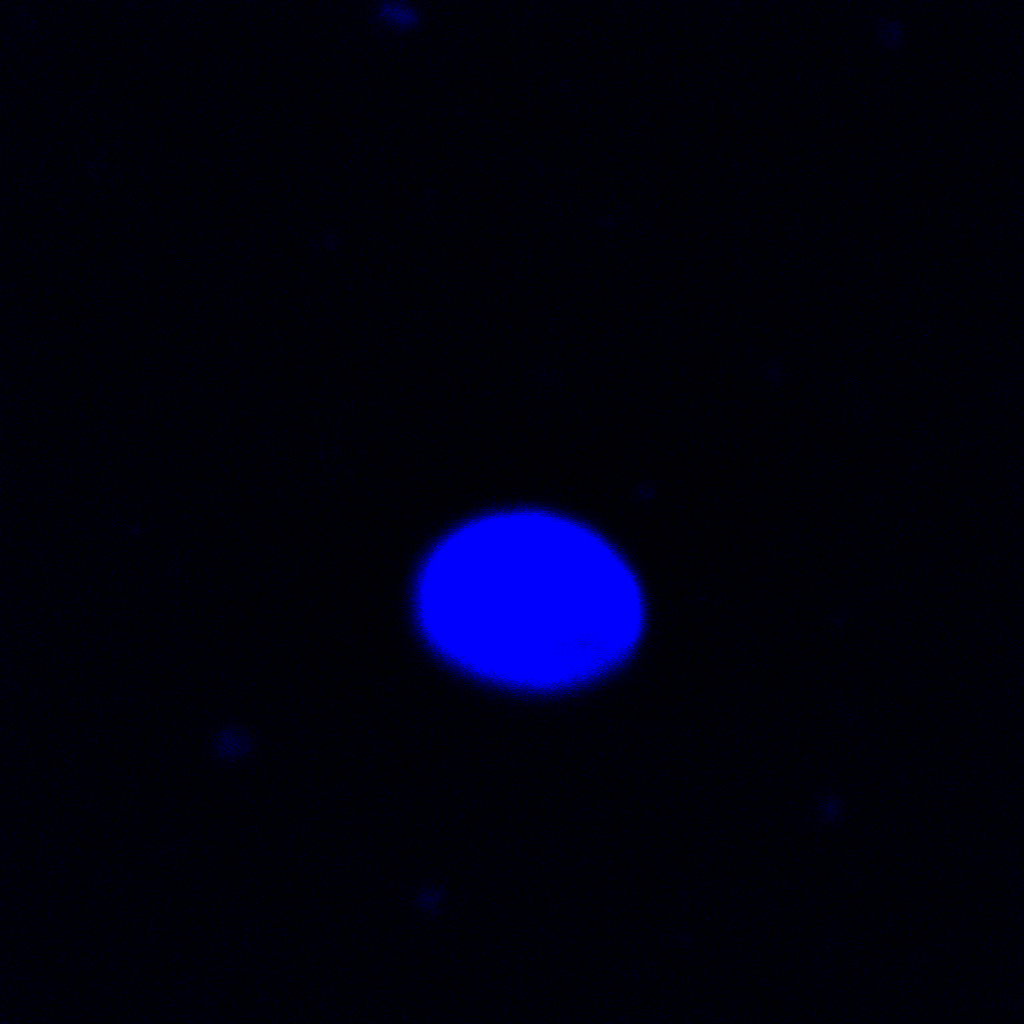

Supplement: Supplementary file 2 [file Data_Sheet_2.ZIP › 2-Mitophagy/Image37-H.tif]

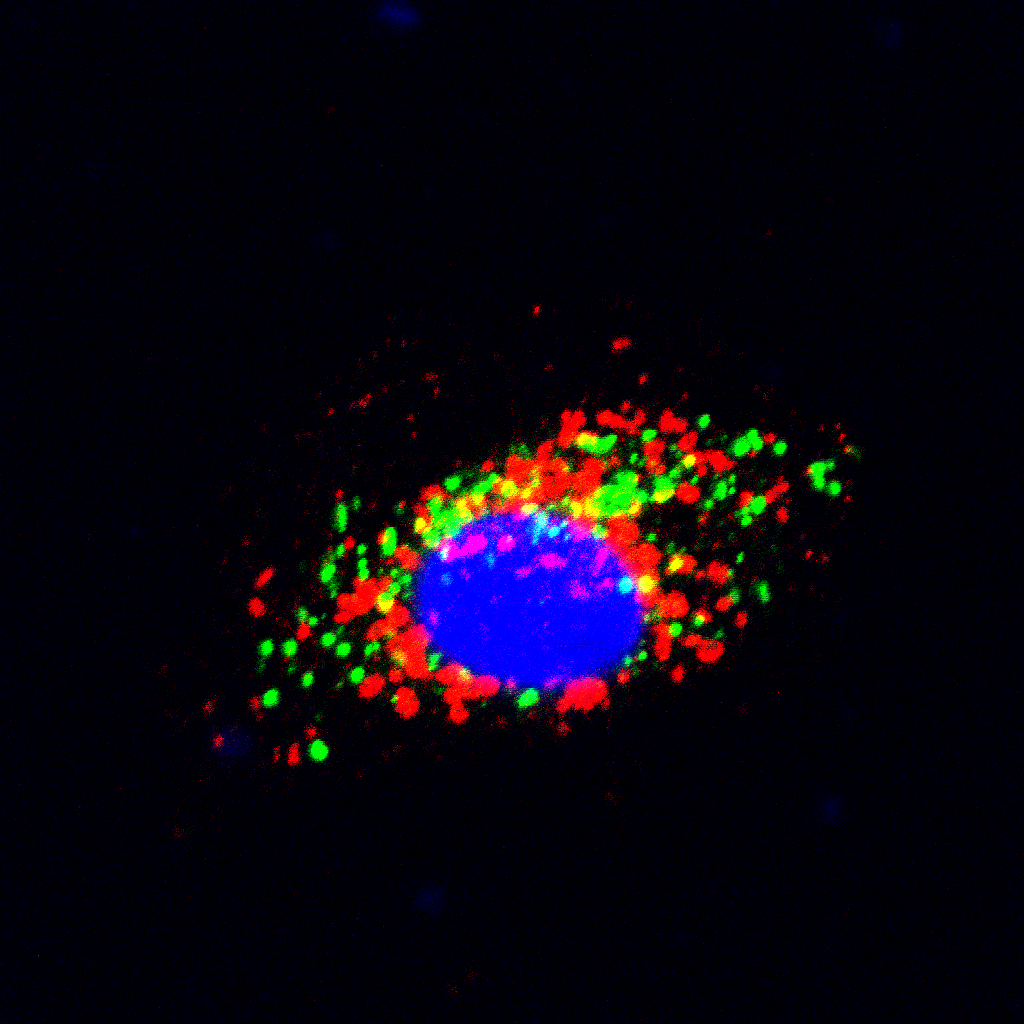

Supplement: Supplementary file 2 [file Data_Sheet_2.ZIP › 2-Mitophagy/Image37-NG.tif]

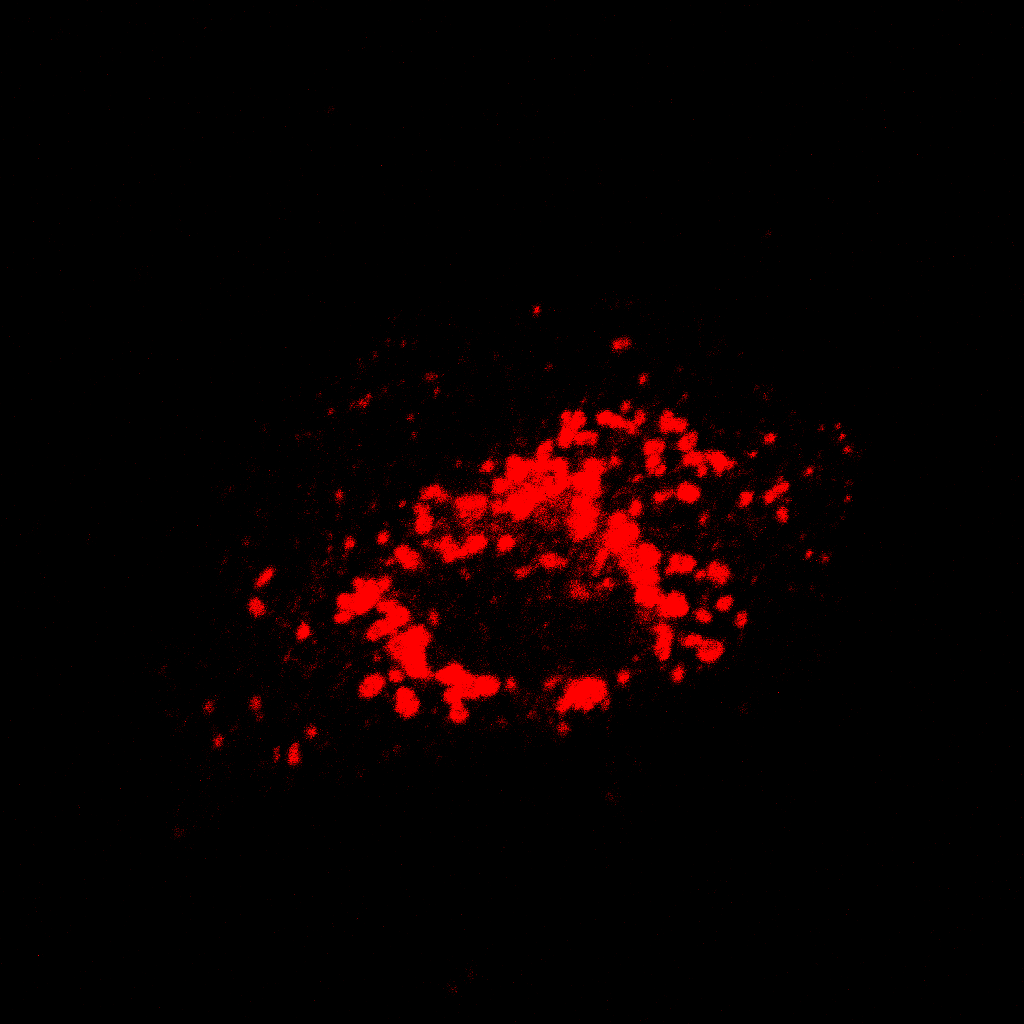

Supplement: Supplementary file 2 [file Data_Sheet_2.ZIP › 2-Mitophagy/Image37-R.tif]

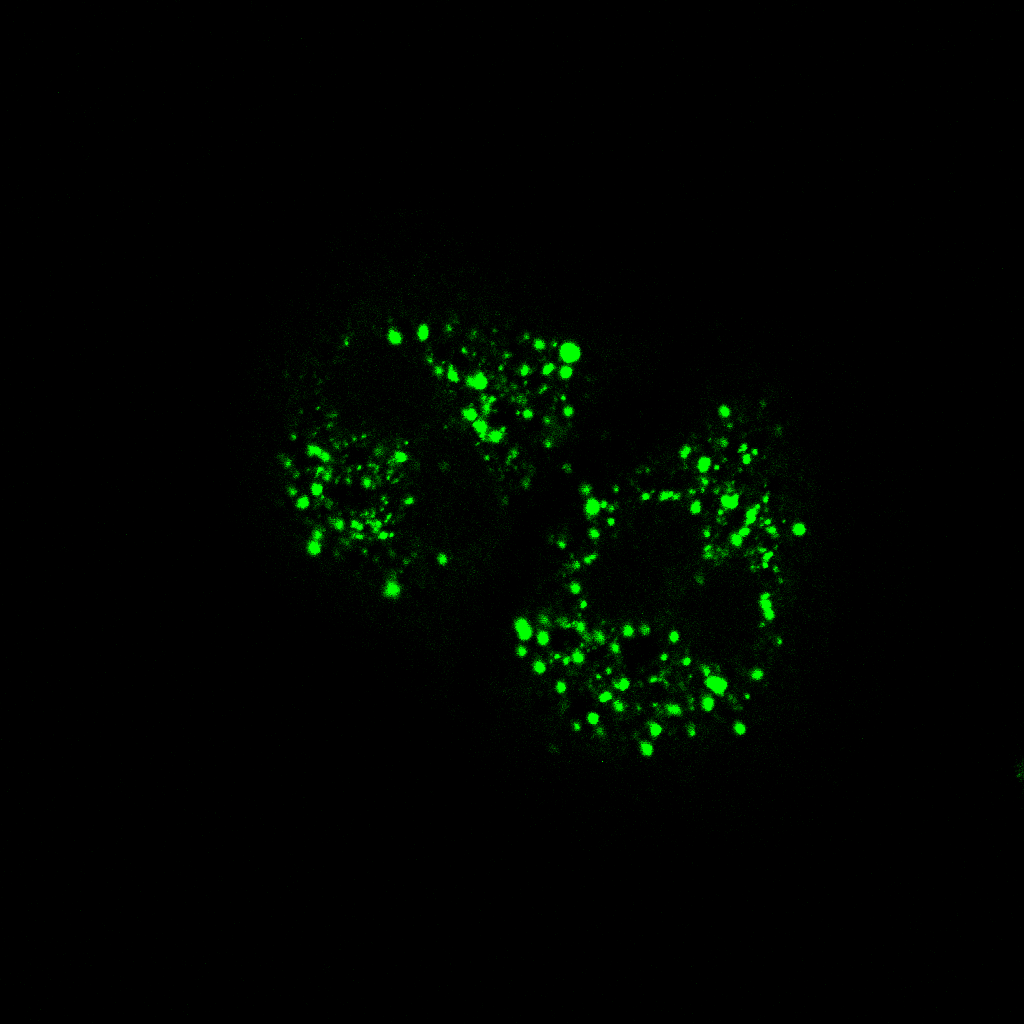

Supplement: Supplementary file 2 [file Data_Sheet_2.ZIP › 2-Mitophagy/Image41-G.tif]

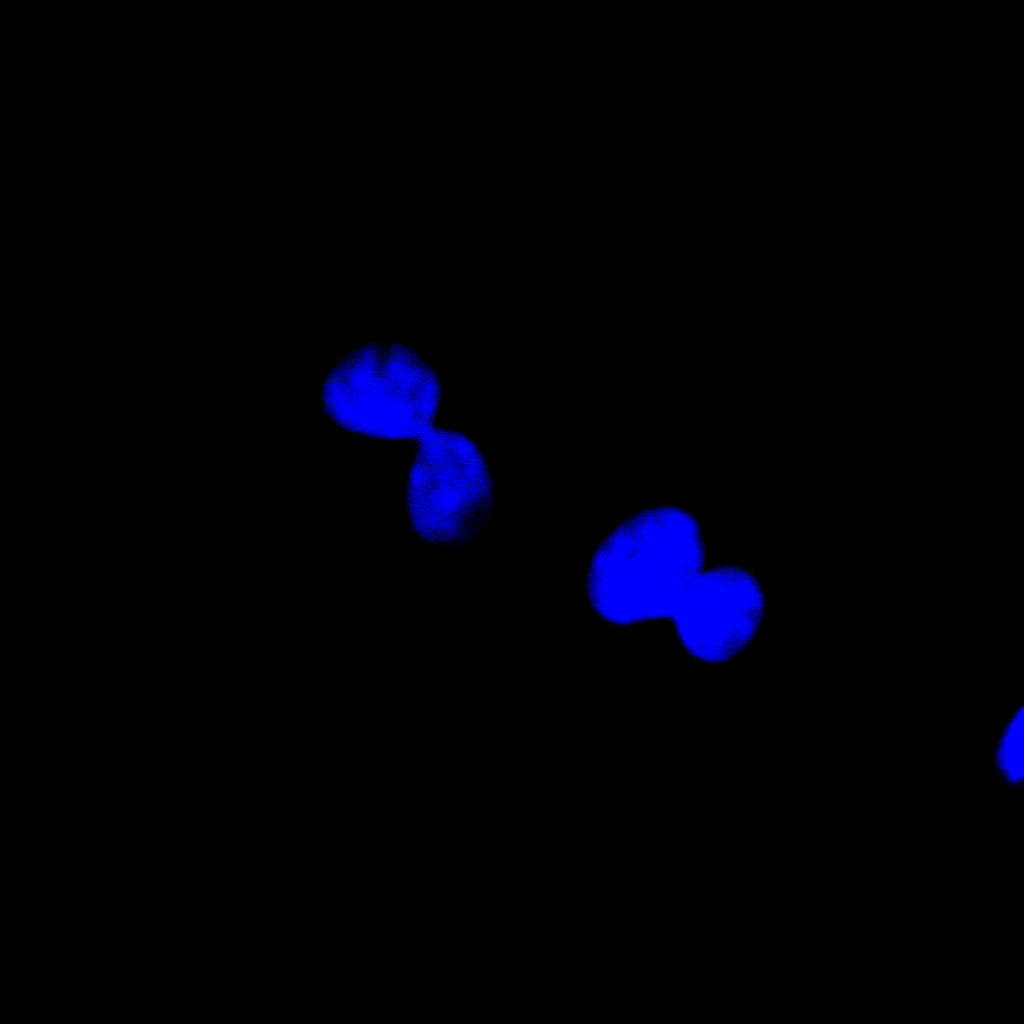

Supplement: Supplementary file 2 [file Data_Sheet_2.ZIP › 2-Mitophagy/Image41-H.tif]

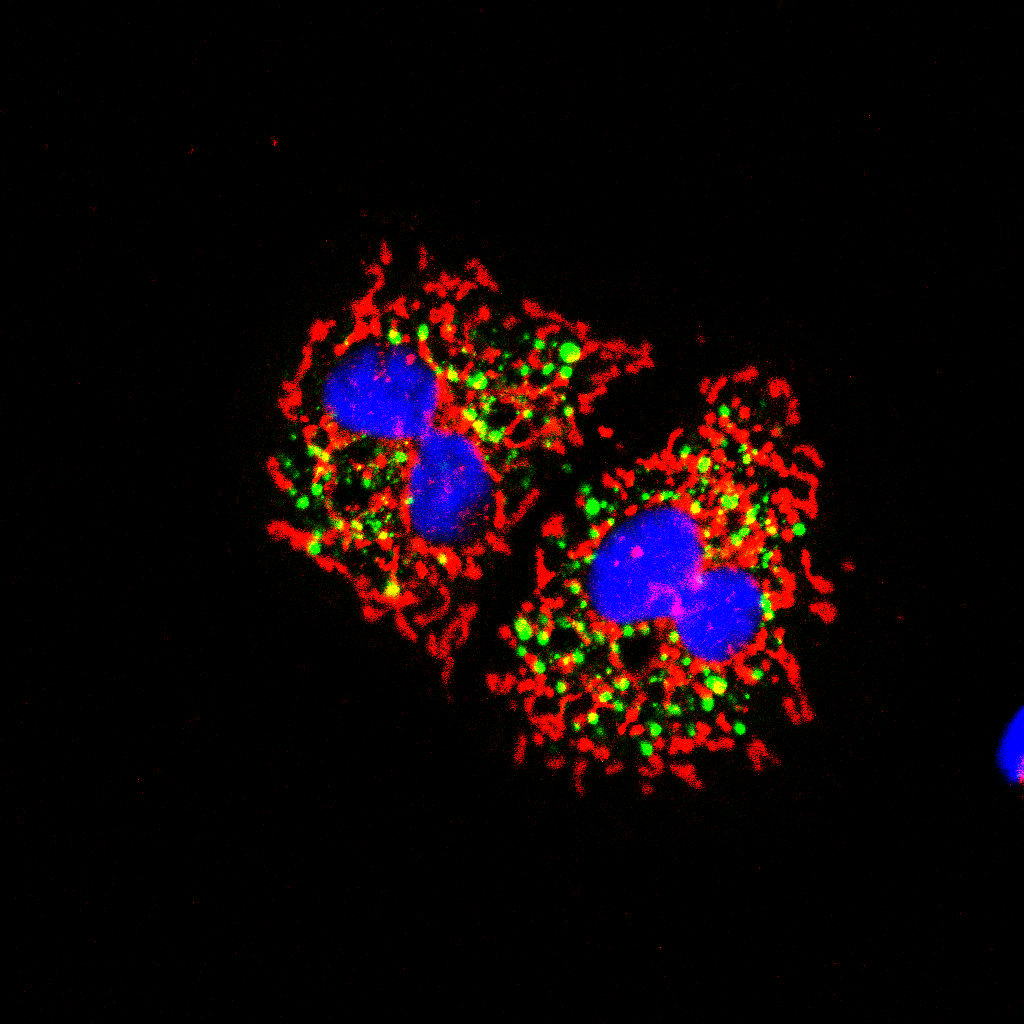

Supplement: Supplementary file 2 [file Data_Sheet_2.ZIP › 2-Mitophagy/Image41-HO.tif]

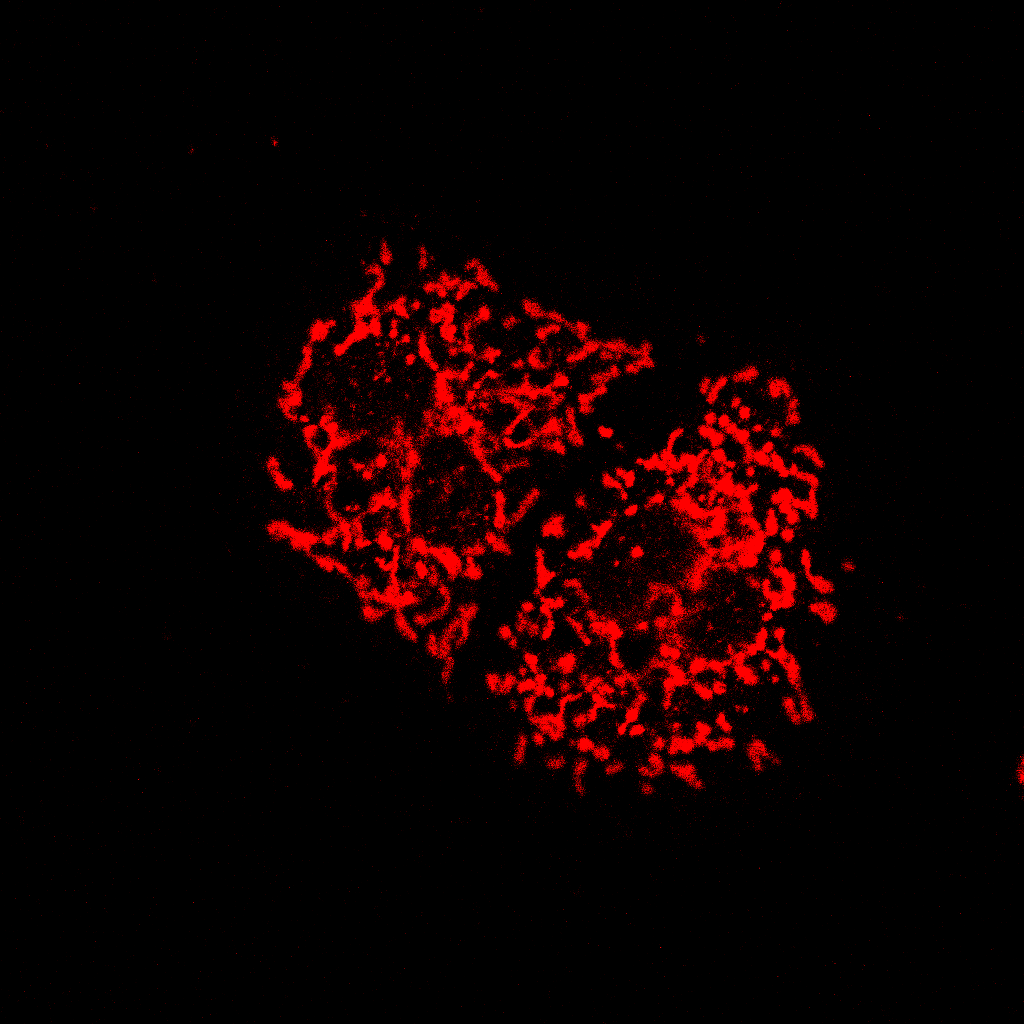

Supplement: Supplementary file 2 [file Data_Sheet_2.ZIP › 2-Mitophagy/Image41-R.tif]

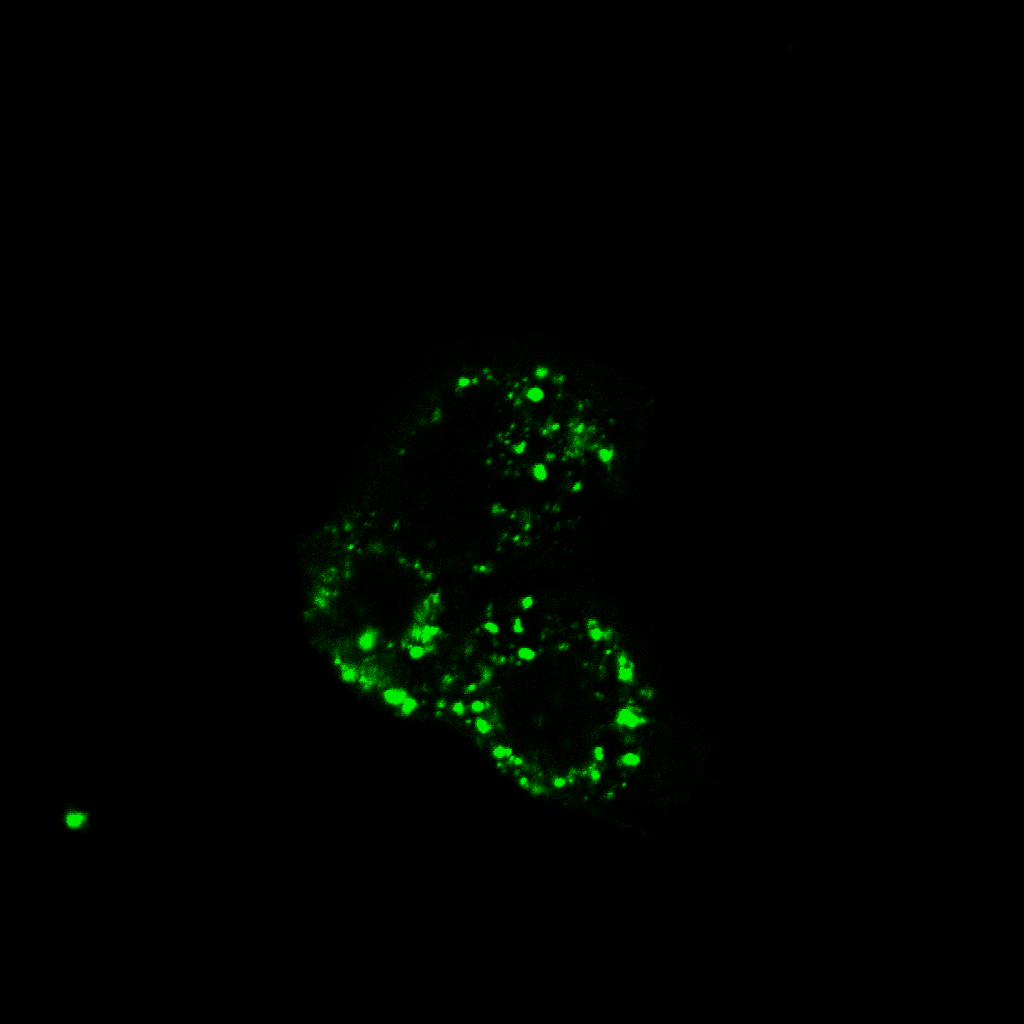

Supplement: Supplementary file 2 [file Data_Sheet_2.ZIP › 2-Mitophagy/Image50-G.tif]

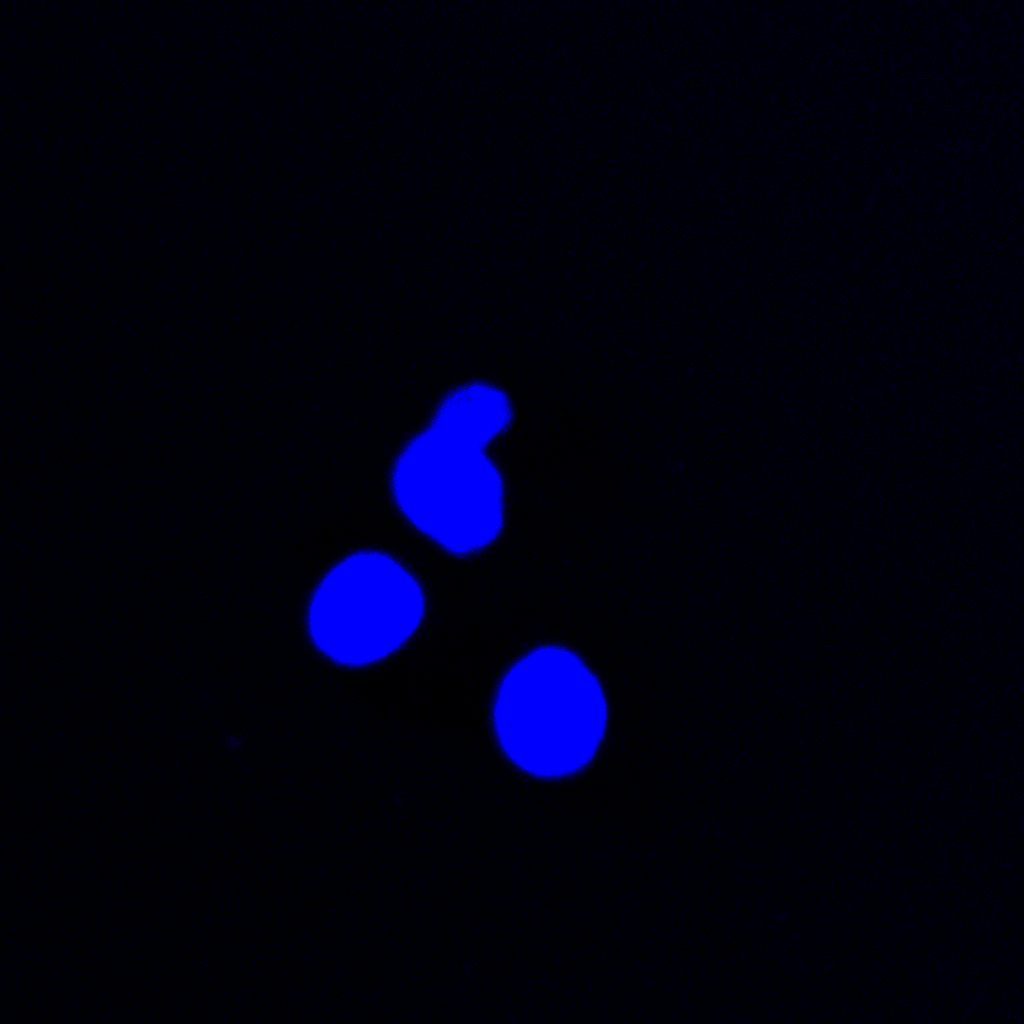

Supplement: Supplementary file 2 [file Data_Sheet_2.ZIP › 2-Mitophagy/Image50-H.tif]

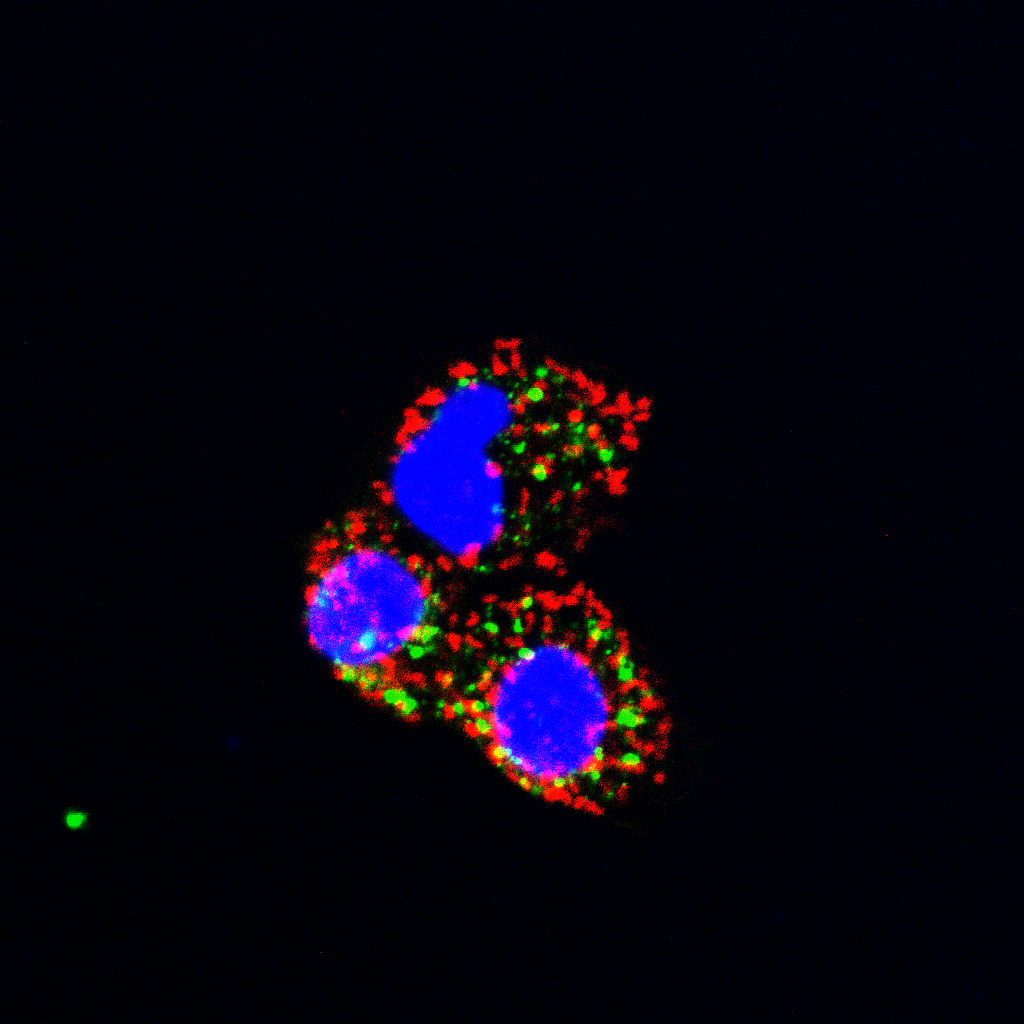

Supplement: Supplementary file 2 [file Data_Sheet_2.ZIP › 2-Mitophagy/Image50-HG.tif]

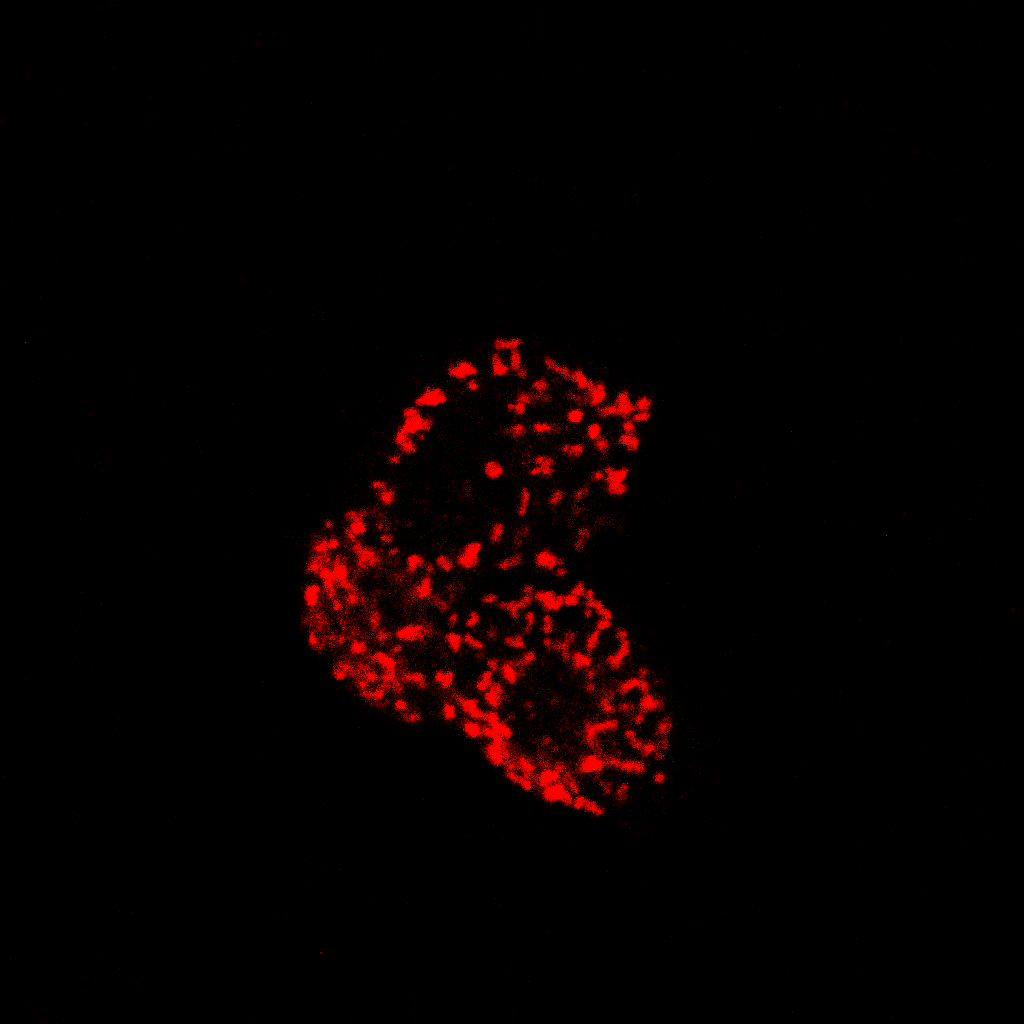

Supplement: Supplementary file 2 [file Data_Sheet_2.ZIP › 2-Mitophagy/Image50-R.tif]

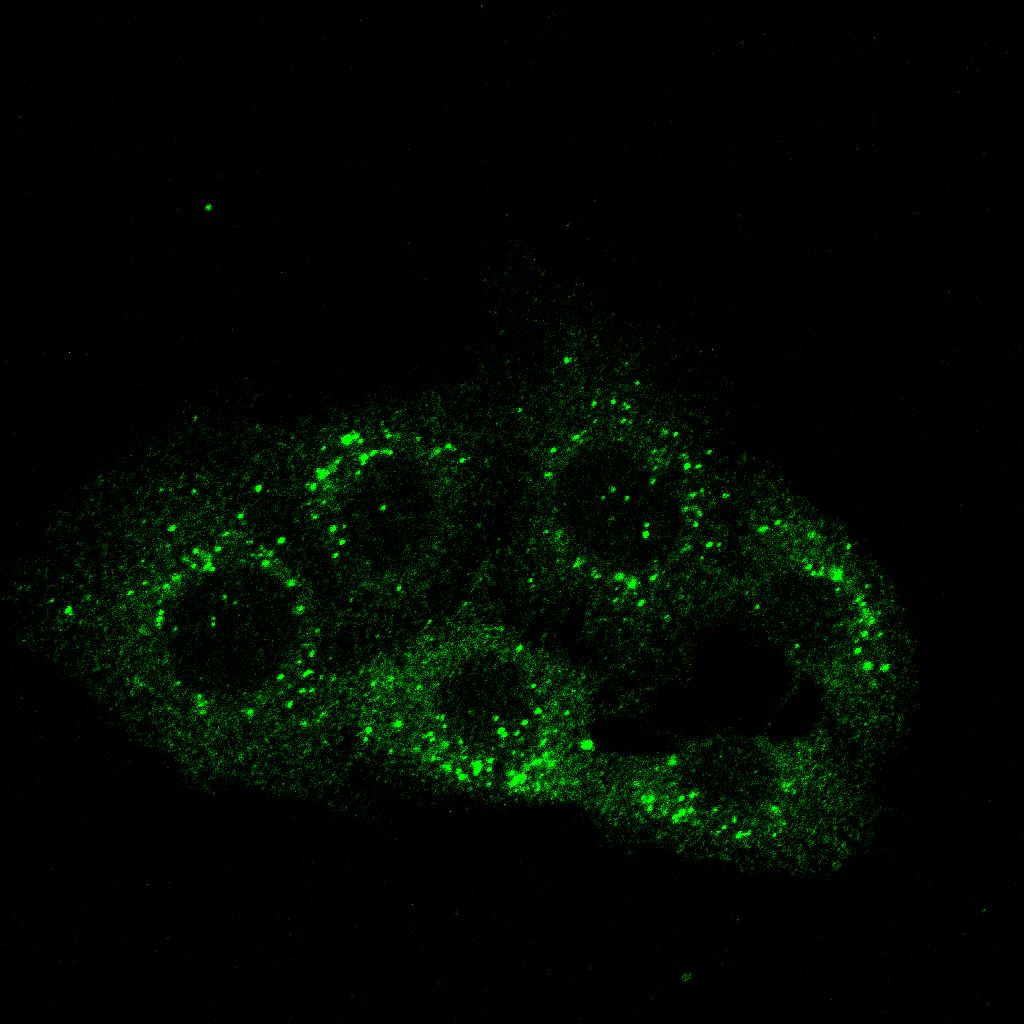

Supplement: Supplementary file 2 [file Data_Sheet_2.ZIP › 2-Mitophagy/Image56-G.tif]

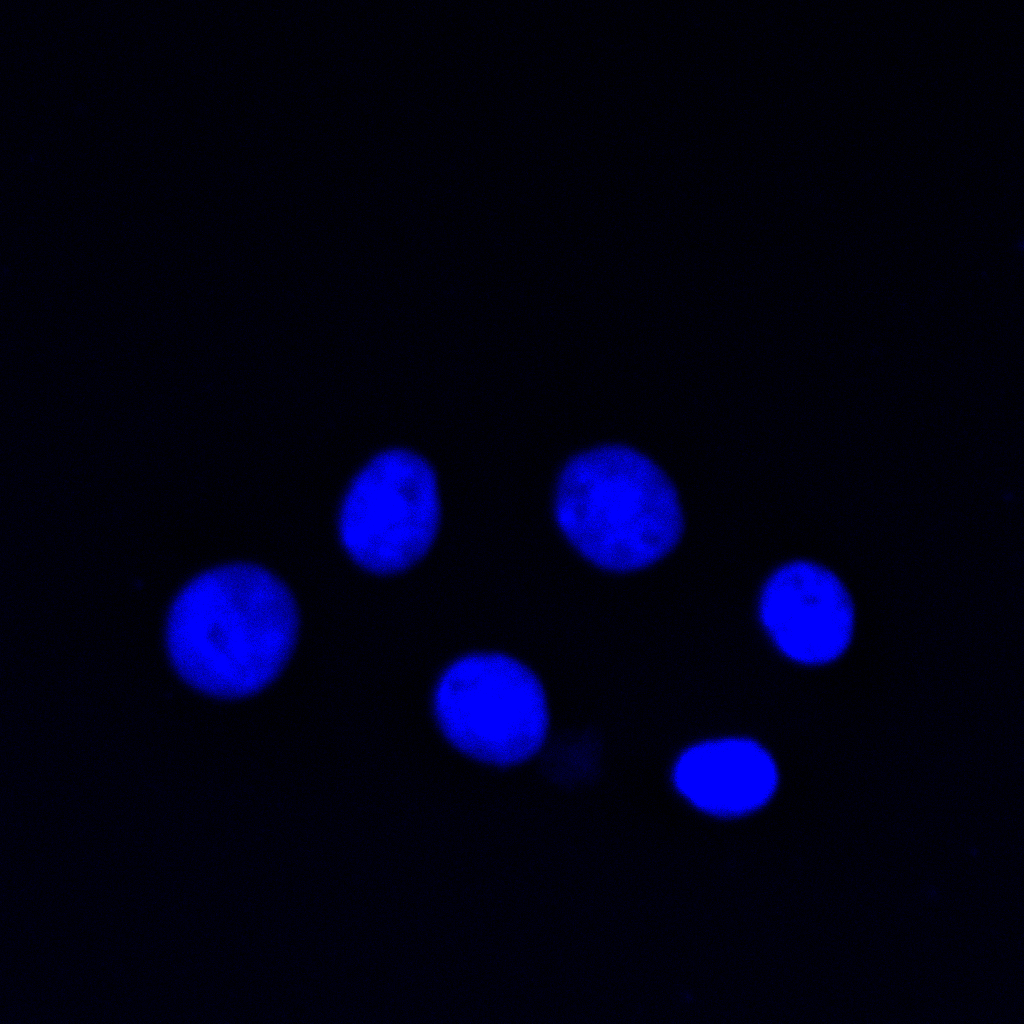

Supplement: Supplementary file 2 [file Data_Sheet_2.ZIP › 2-Mitophagy/Image56-H.tif]

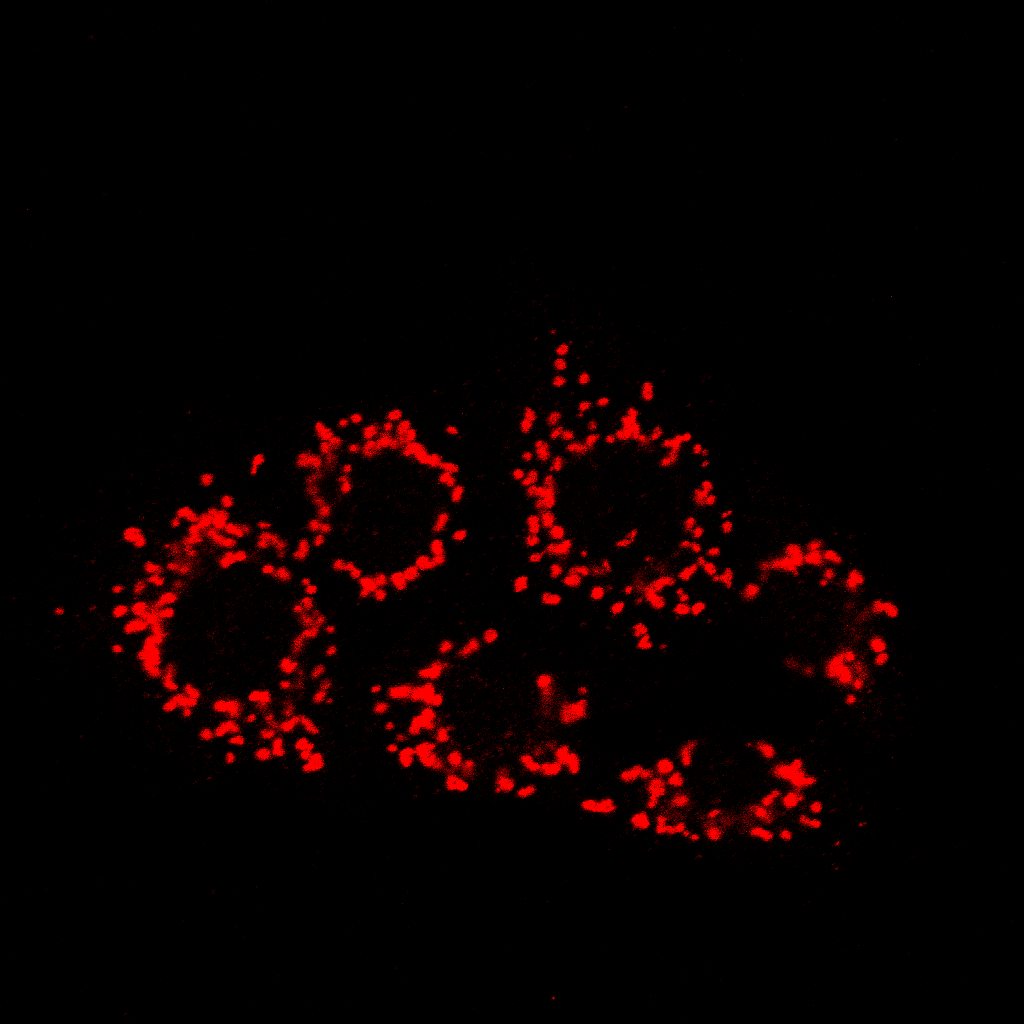

Supplement: Supplementary file 2 [file Data_Sheet_2.ZIP › 2-Mitophagy/Image56-R.tif]

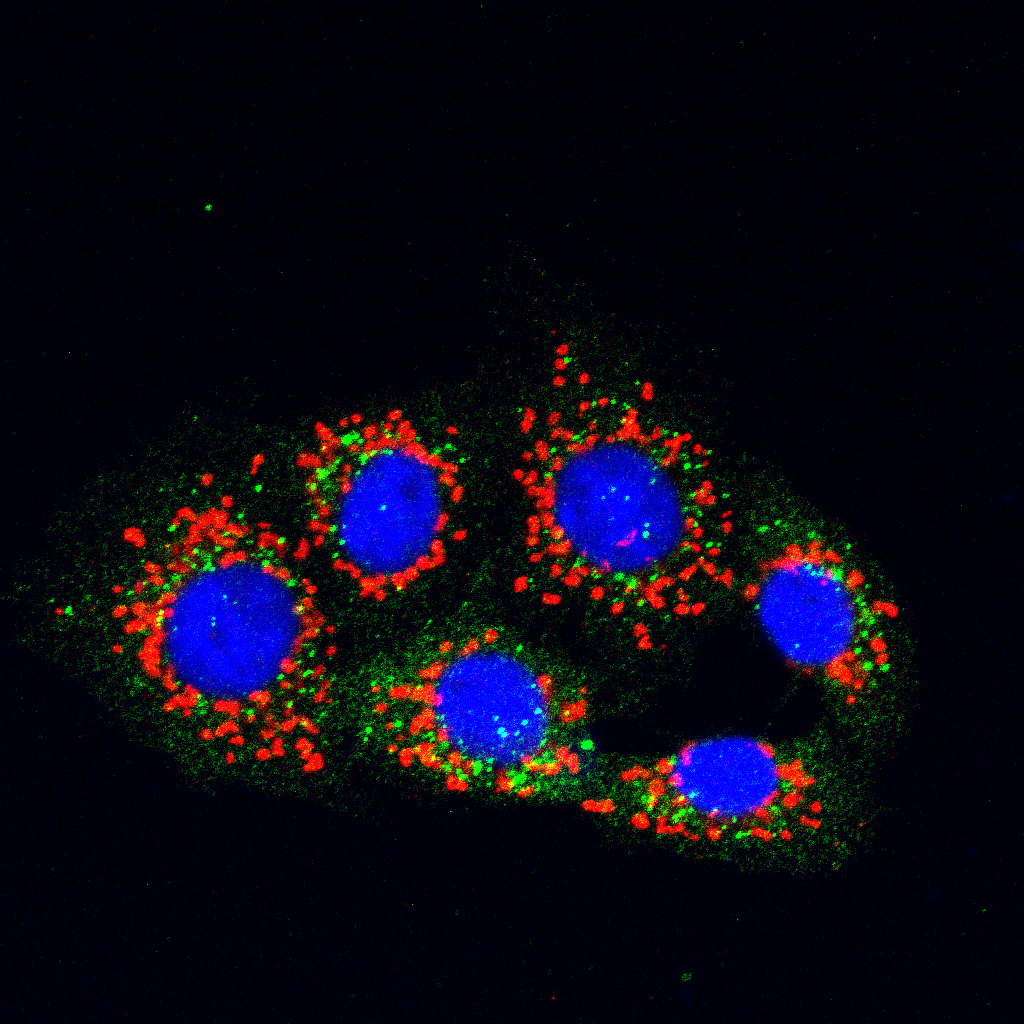

Supplement: Supplementary file 2 [file Data_Sheet_2.ZIP › 2-Mitophagy/Image56.tif]

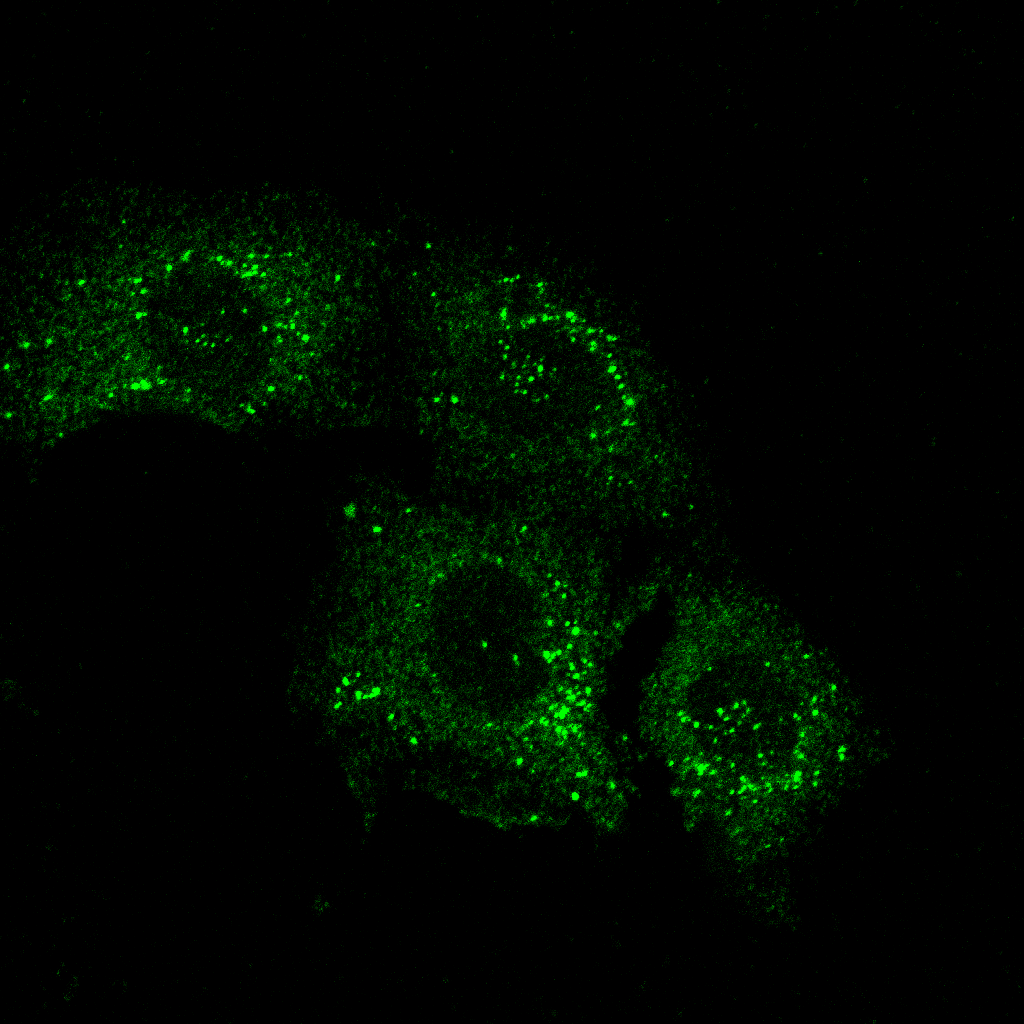

Supplement: Supplementary file 2 [file Data_Sheet_2.ZIP › 2-Mitophagy/Image59-G.tif]

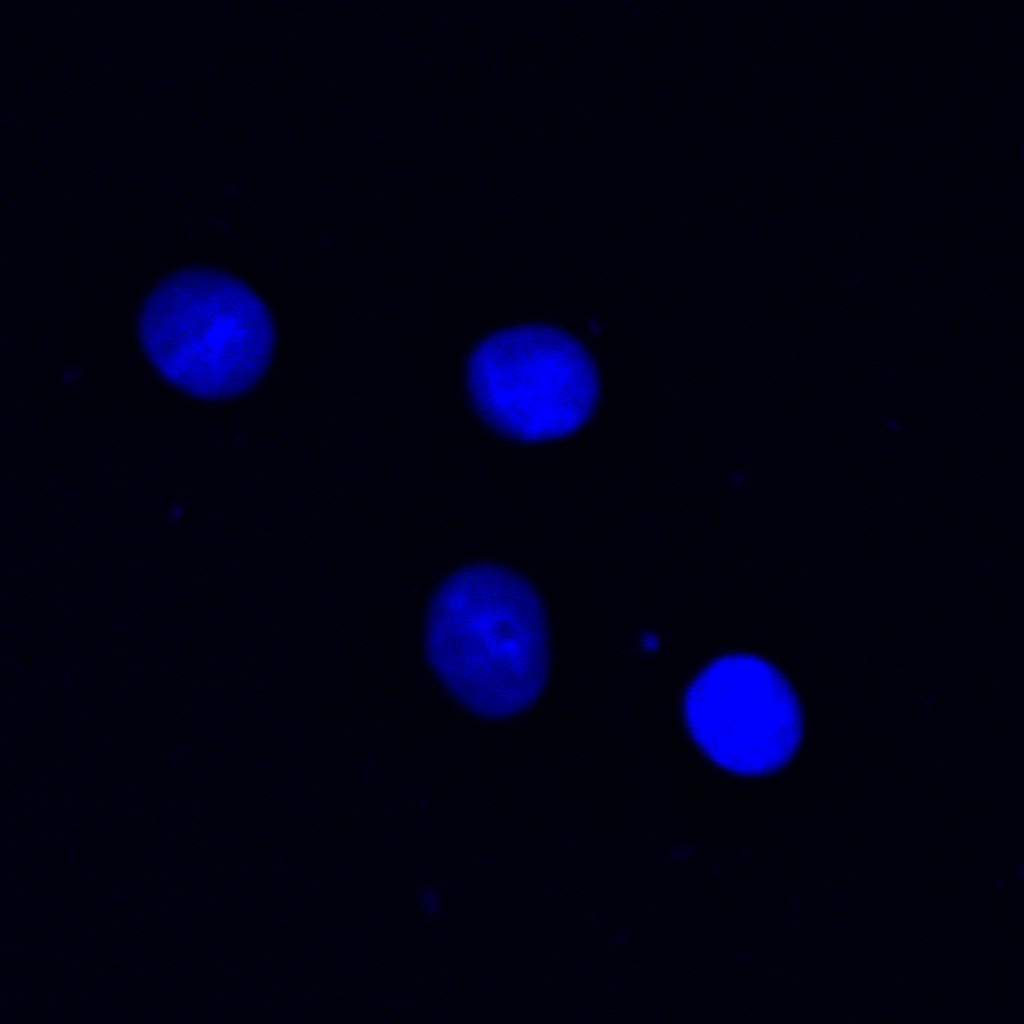

Supplement: Supplementary file 2 [file Data_Sheet_2.ZIP › 2-Mitophagy/Image59-H.tif]

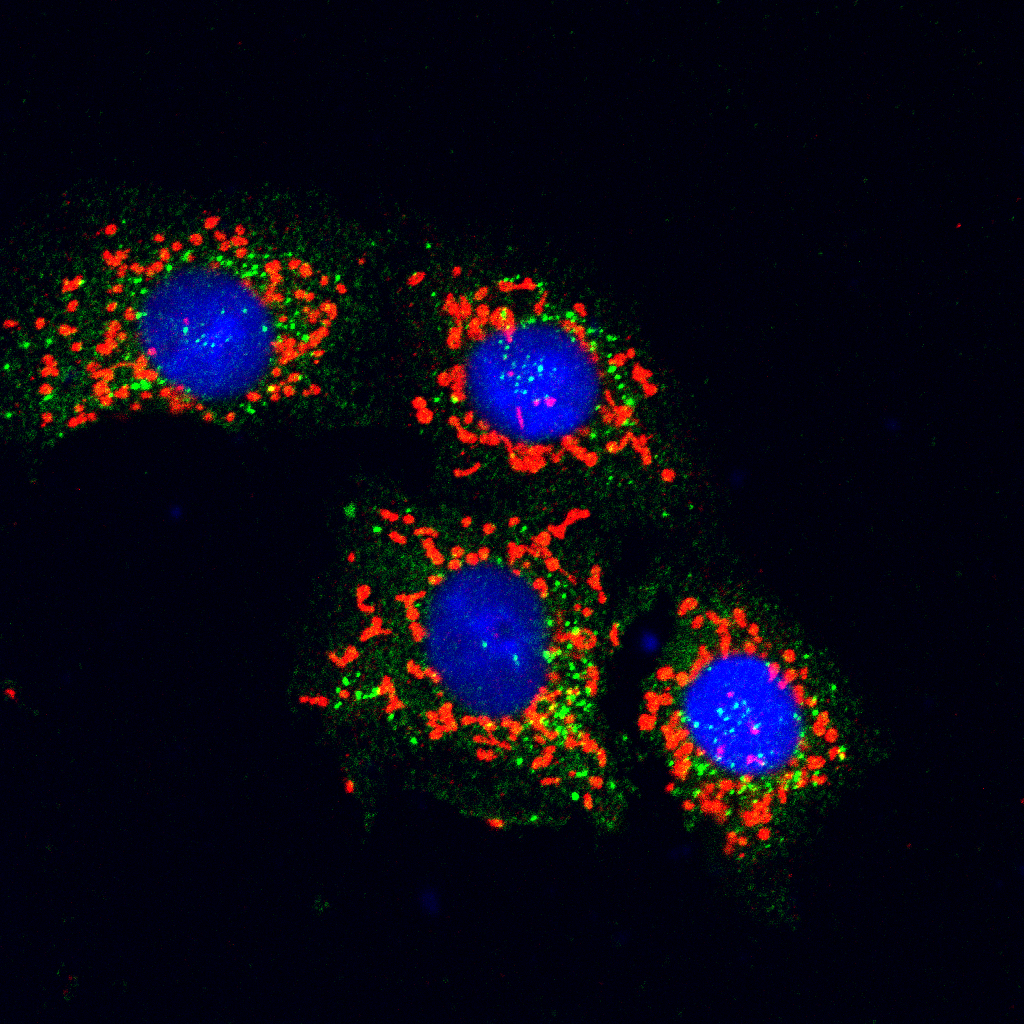

Supplement: Supplementary file 2 [file Data_Sheet_2.ZIP › 2-Mitophagy/Image59-HG+YC-1.tif]

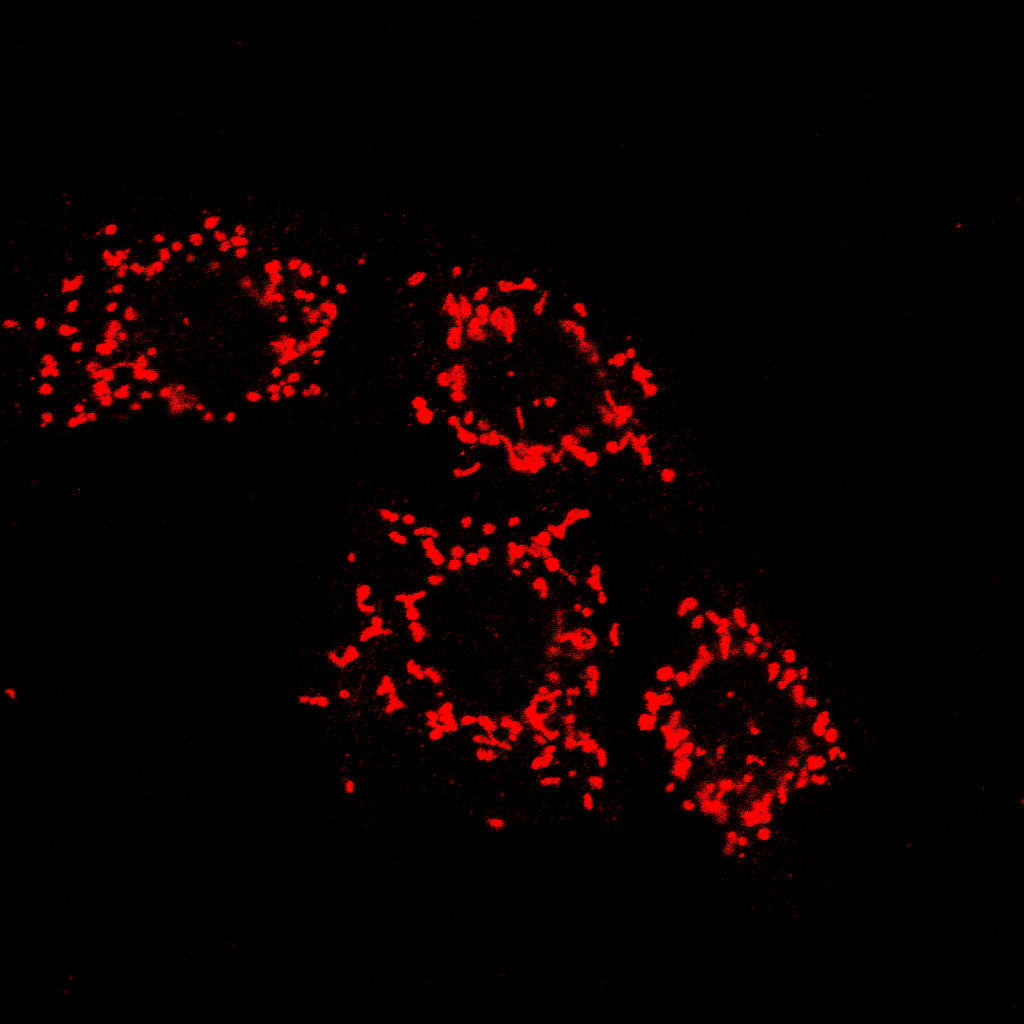

Supplement: Supplementary file 2 [file Data_Sheet_2.ZIP › 2-Mitophagy/Image59-R.tif]

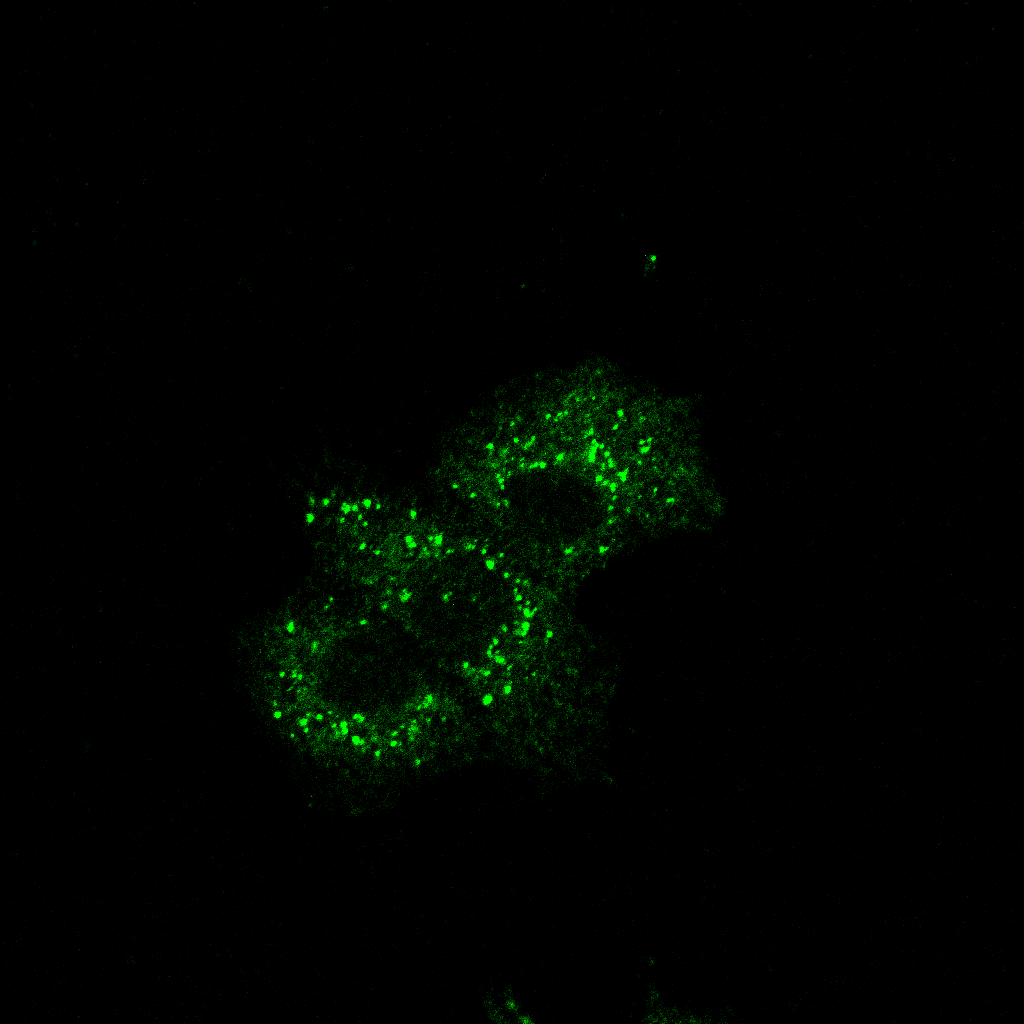

Supplement: Supplementary file 2 [file Data_Sheet_2.ZIP › 2-Mitophagy/Image64-G.tif]

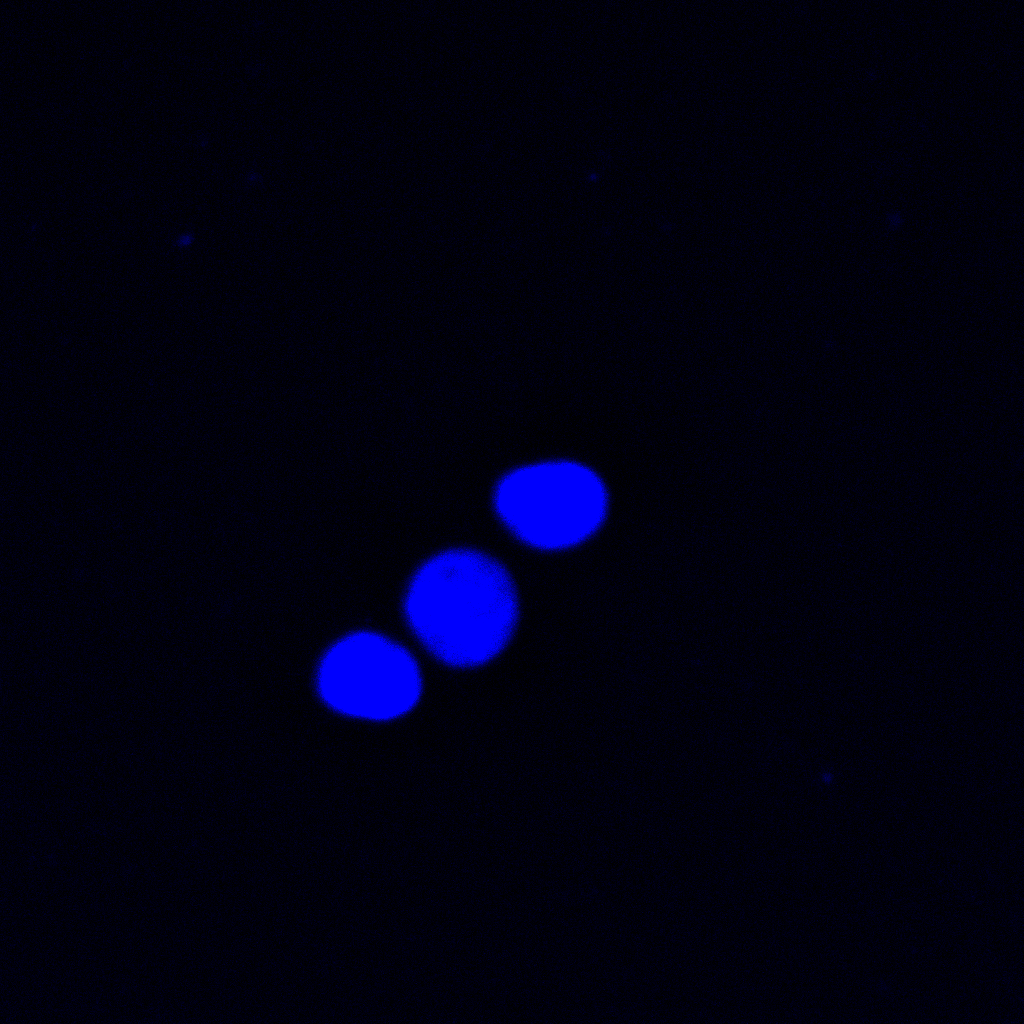

Supplement: Supplementary file 2 [file Data_Sheet_2.ZIP › 2-Mitophagy/Image64-H.tif]

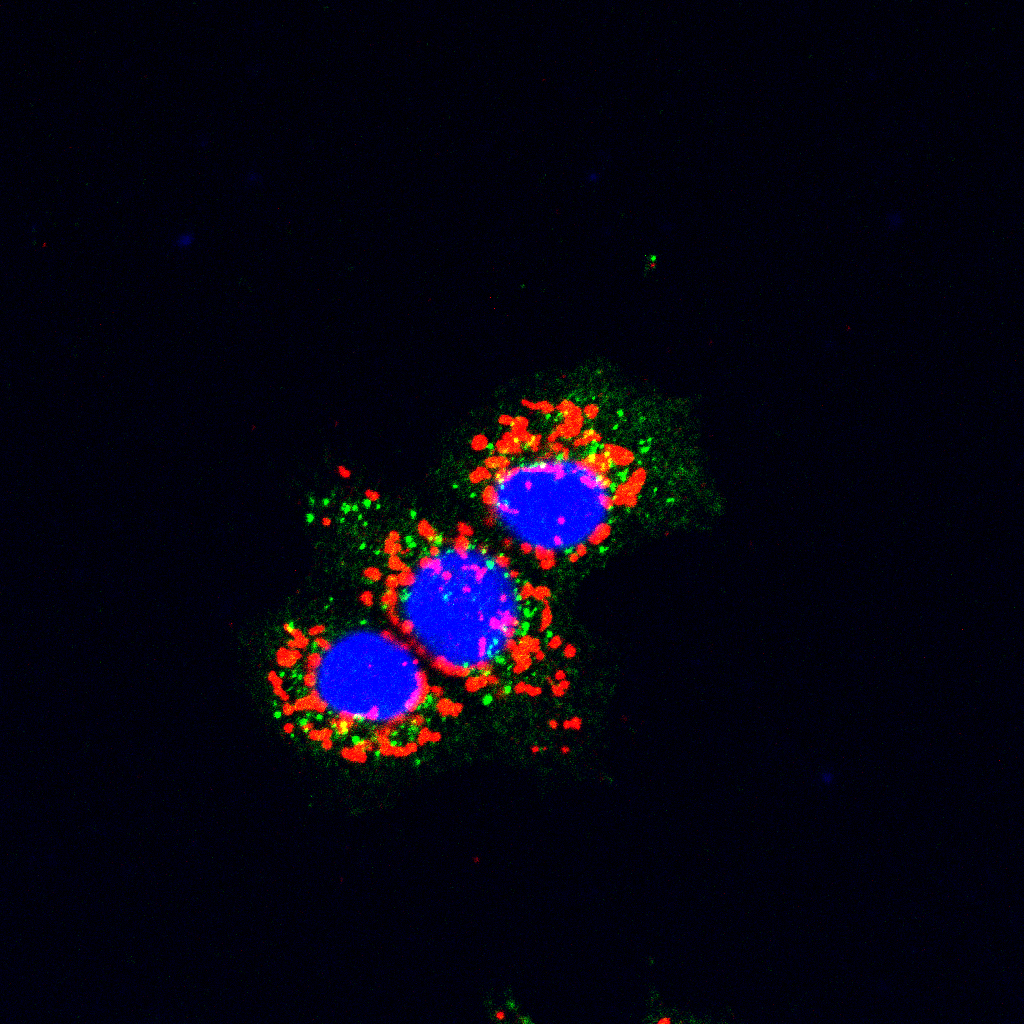

Supplement: Supplementary file 2 [file Data_Sheet_2.ZIP › 2-Mitophagy/Image64-HG+NAC+YC-1.tif]

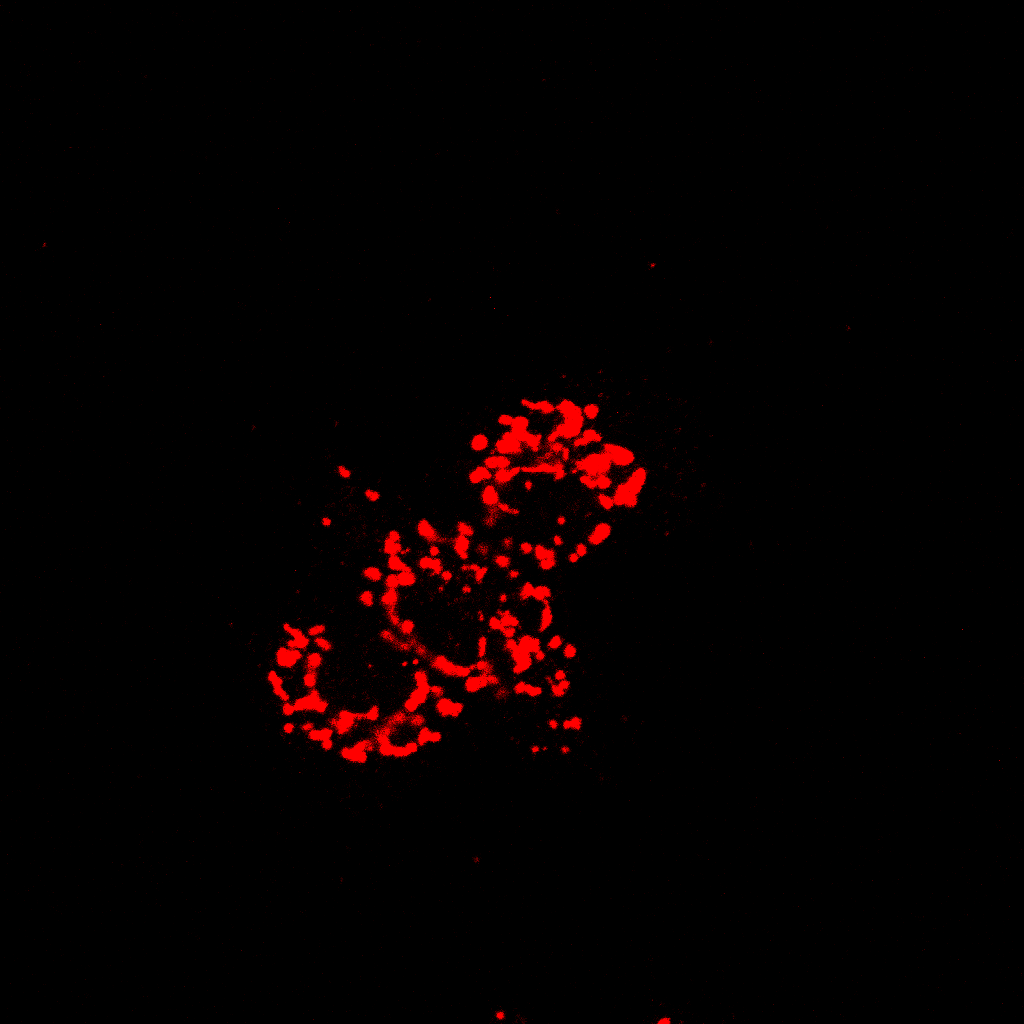

Supplement: Supplementary file 2 [file Data_Sheet_2.ZIP › 2-Mitophagy/Image64-R.tif]

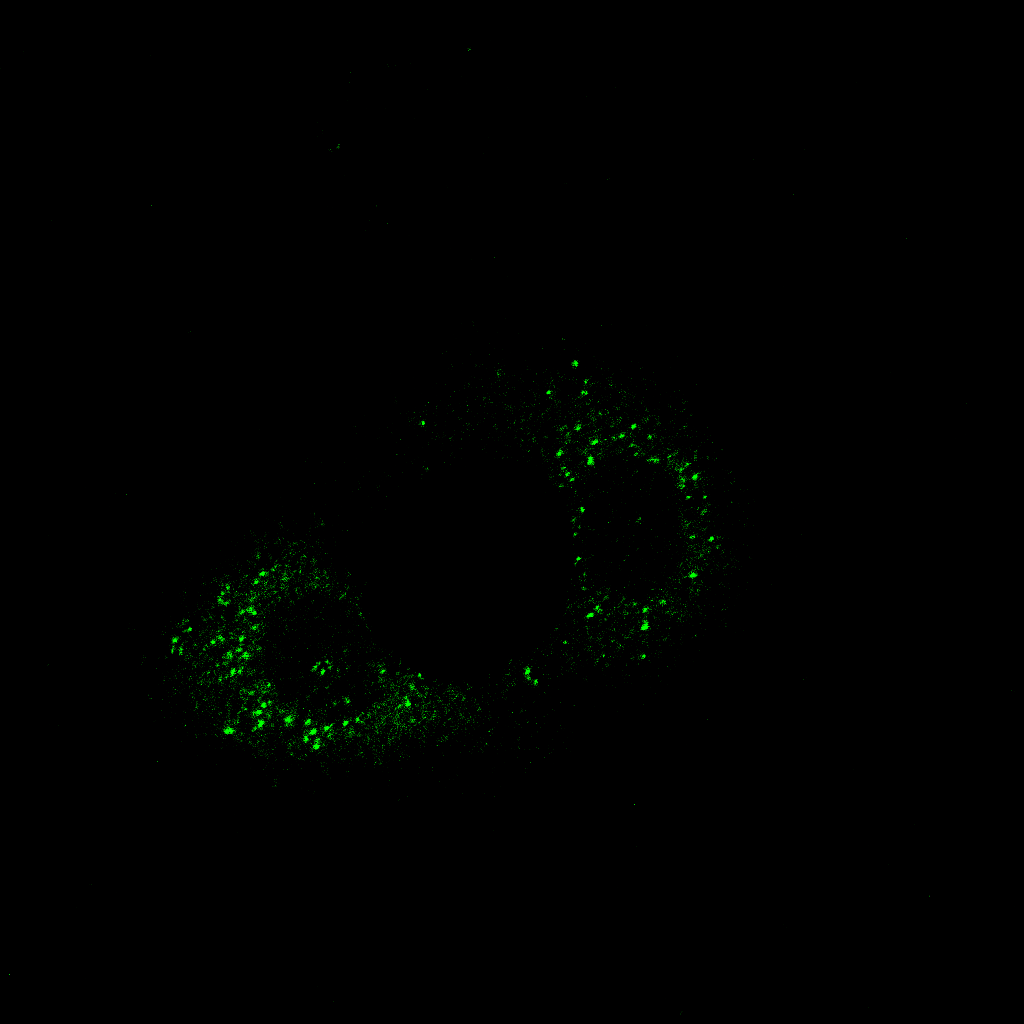

Supplement: Supplementary file 2 [file Data_Sheet_2.ZIP › 2-Mitophagy/Image67-G.tif]

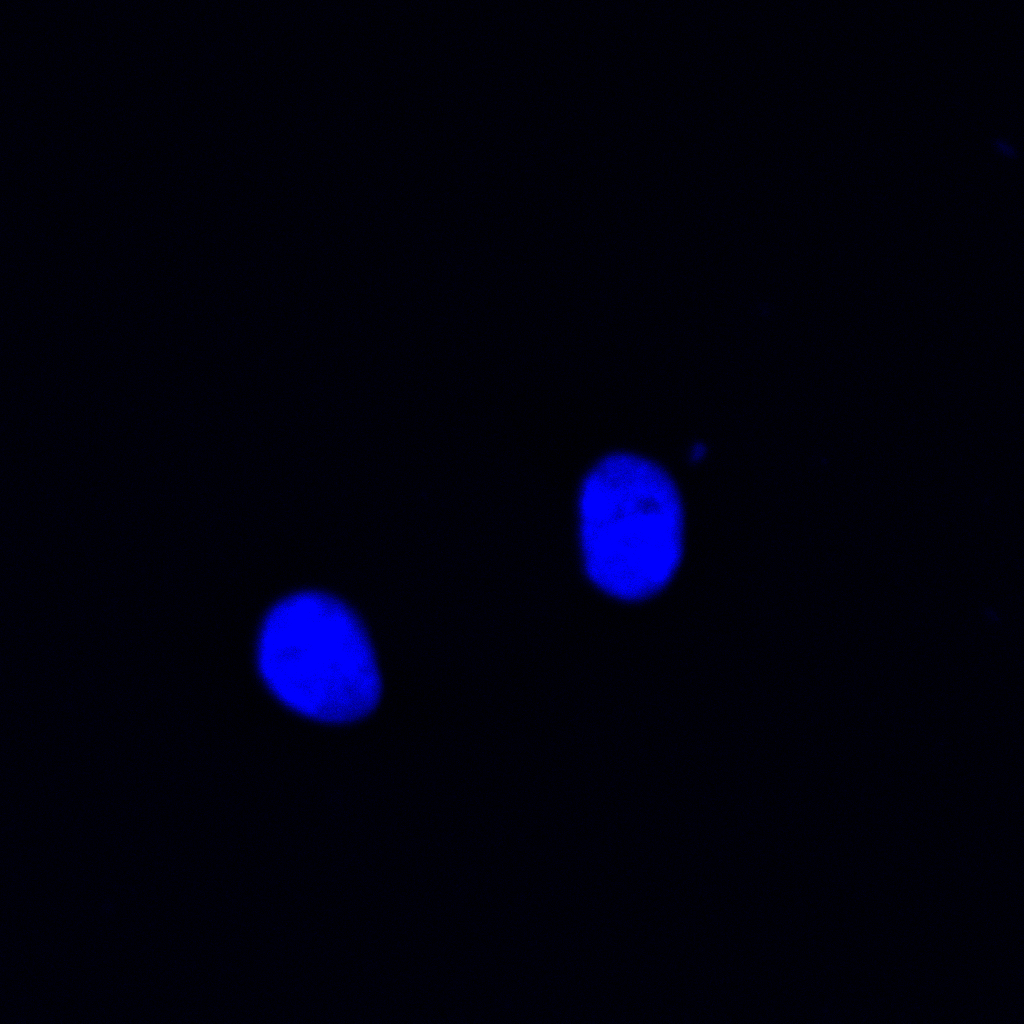

Supplement: Supplementary file 2 [file Data_Sheet_2.ZIP › 2-Mitophagy/Image67-H.tif]

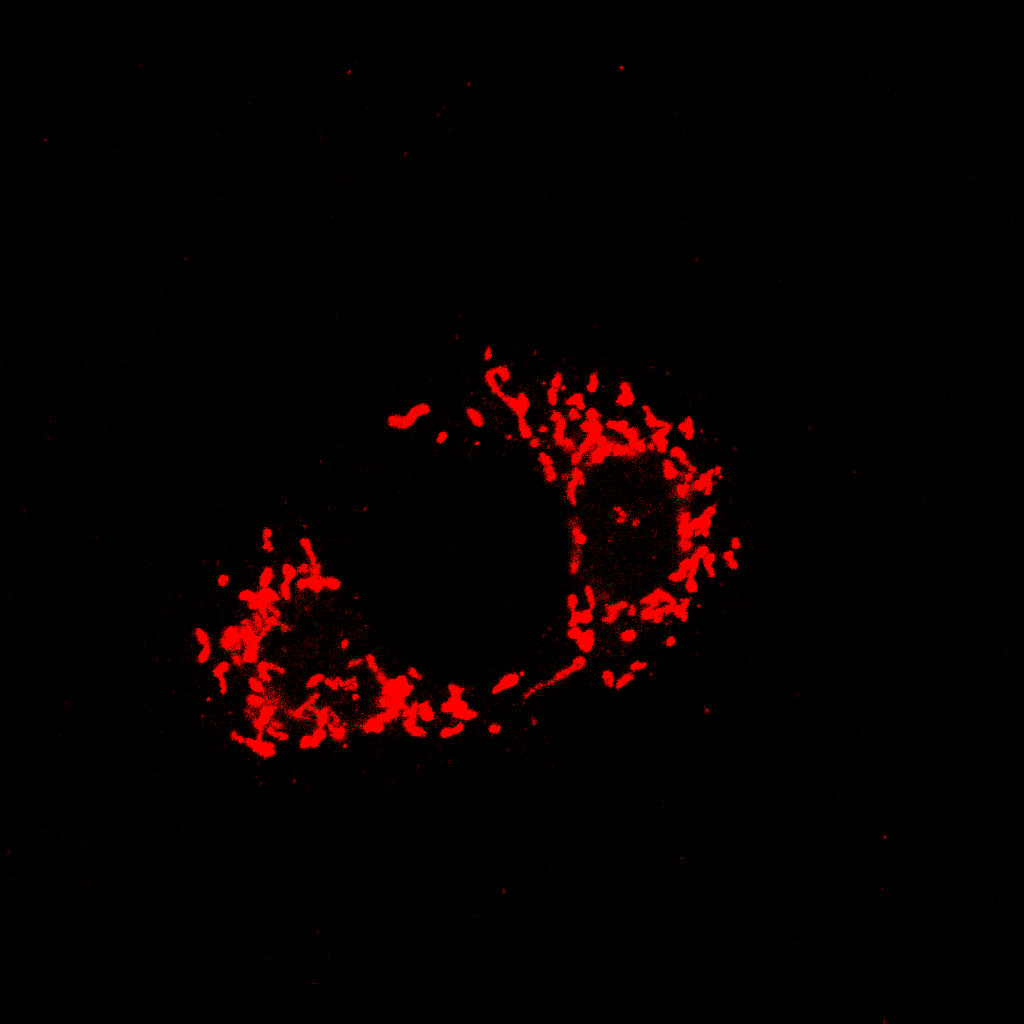

Supplement: Supplementary file 2 [file Data_Sheet_2.ZIP › 2-Mitophagy/Image67-R.tif]

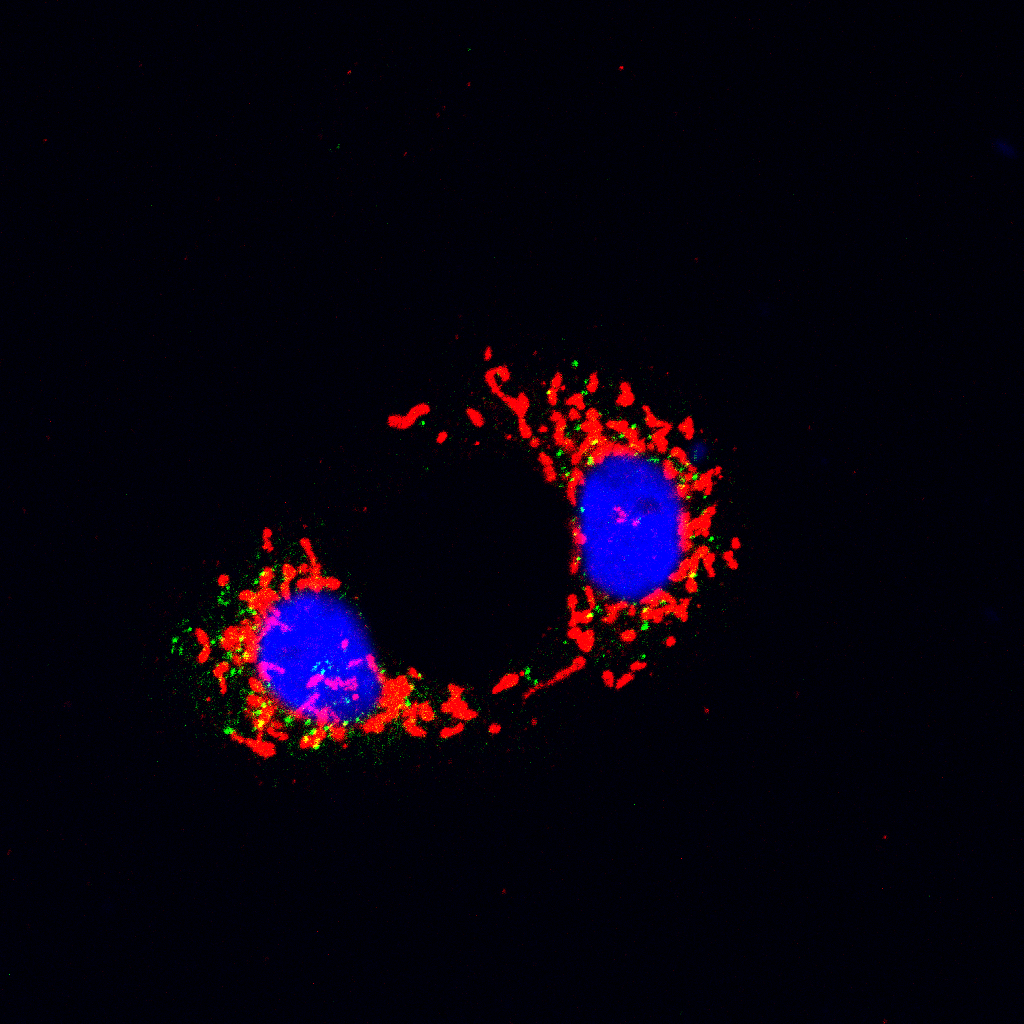

Supplement: Supplementary file 2 [file Data_Sheet_2.ZIP › 2-Mitophagy/Image67.tif]

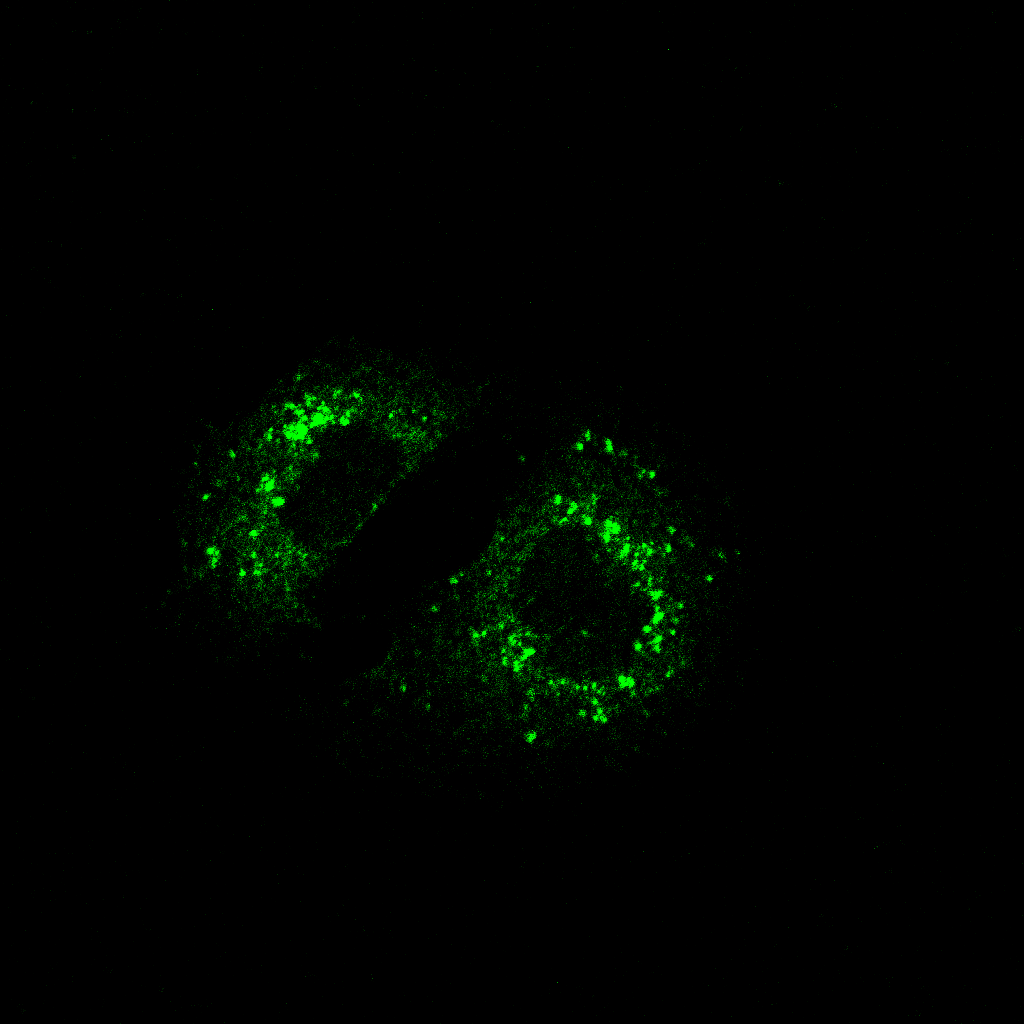

Supplement: Supplementary file 2 [file Data_Sheet_2.ZIP › 2-Mitophagy/Image73-G.tif]

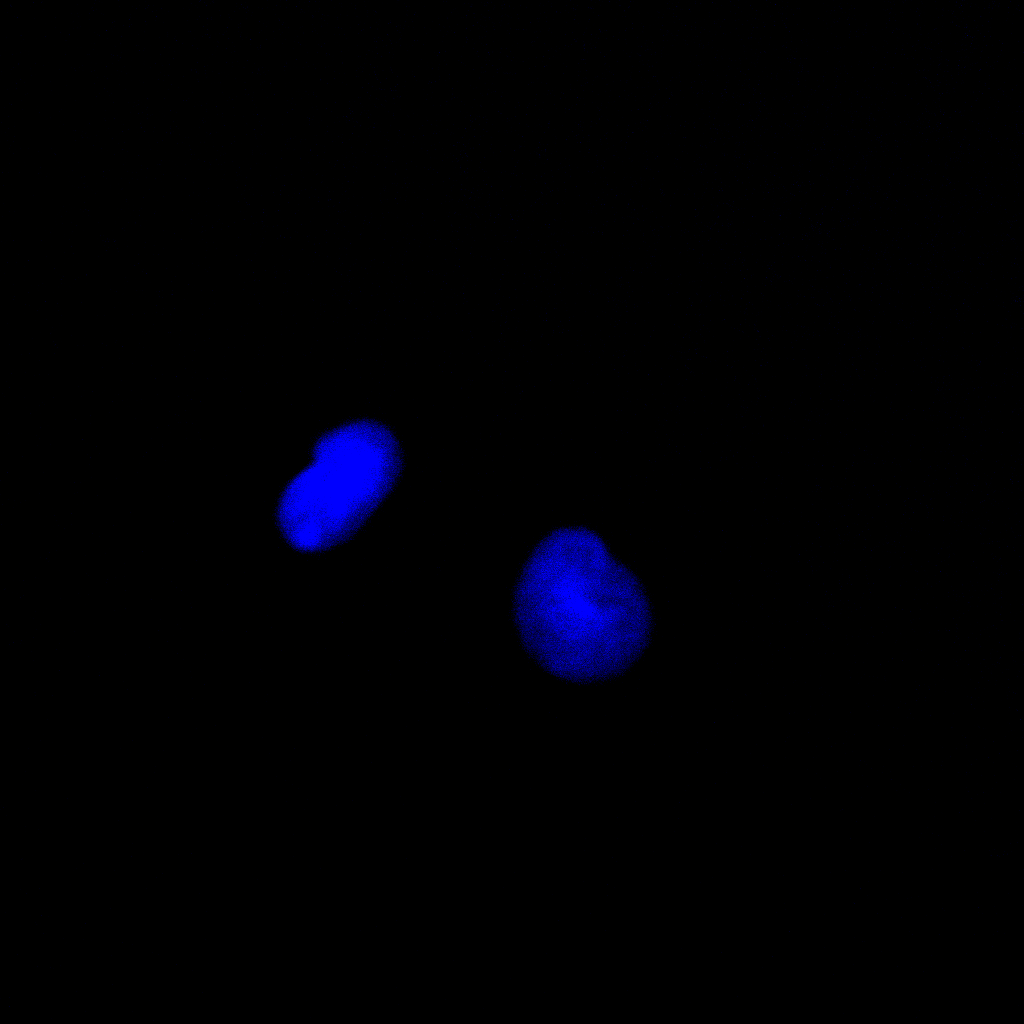

Supplement: Supplementary file 2 [file Data_Sheet_2.ZIP › 2-Mitophagy/Image73-H.tif]

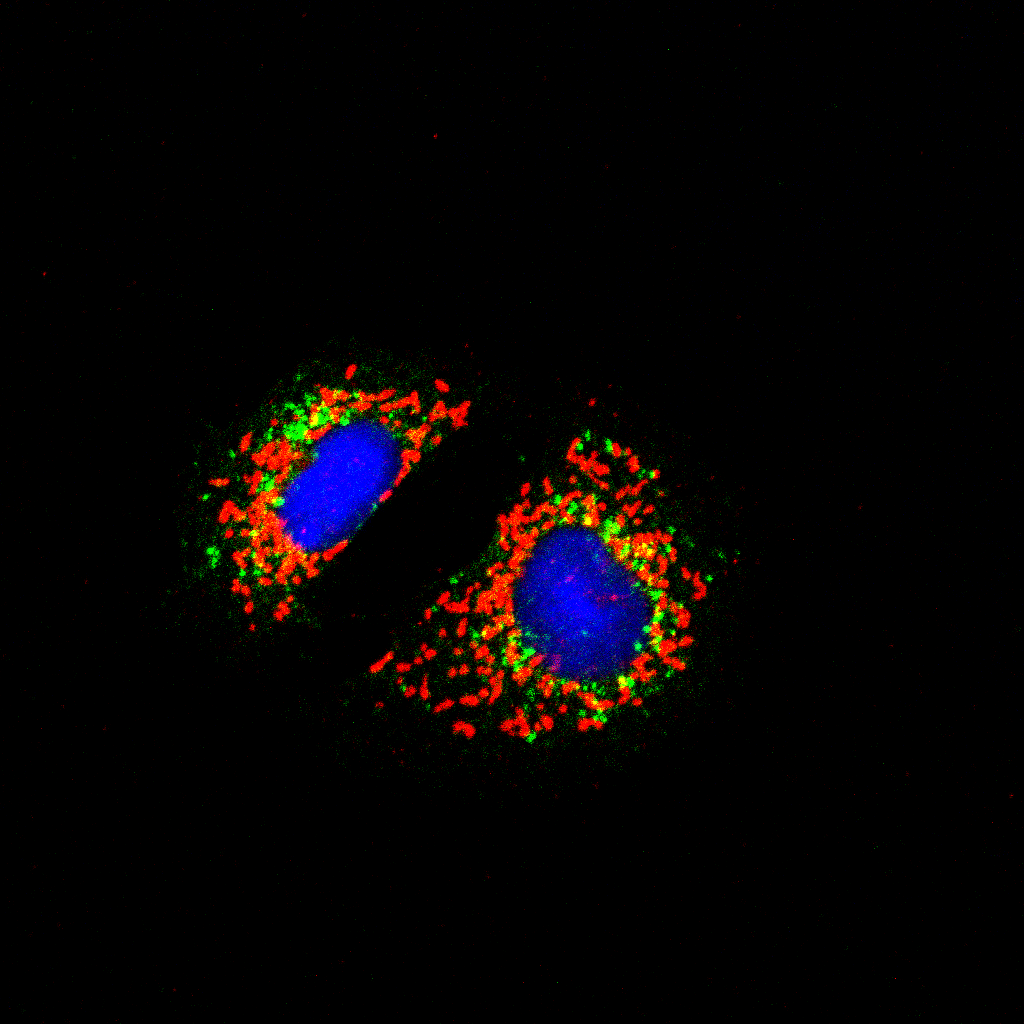

Supplement: Supplementary file 2 [file Data_Sheet_2.ZIP › 2-Mitophagy/Image73-NG.tif]

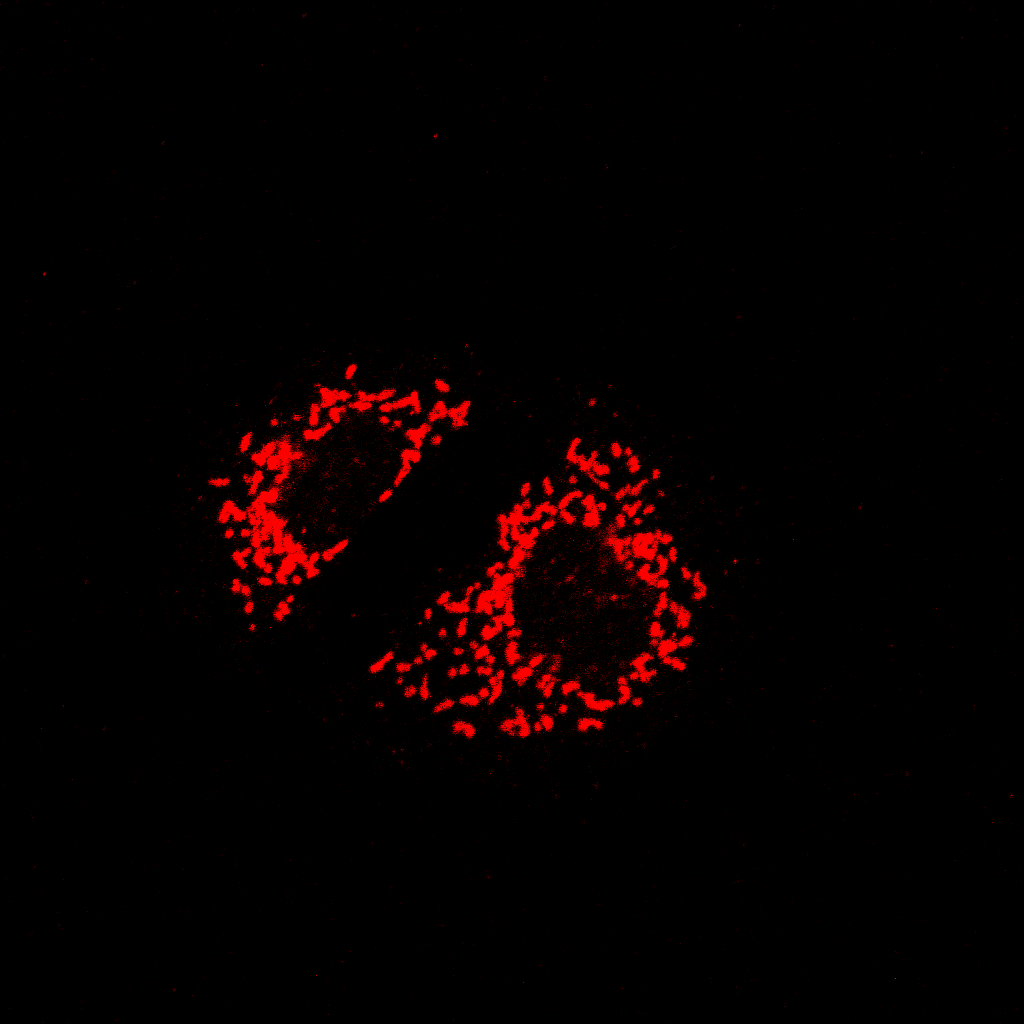

Supplement: Supplementary file 2 [file Data_Sheet_2.ZIP › 2-Mitophagy/Image73-R.tif]

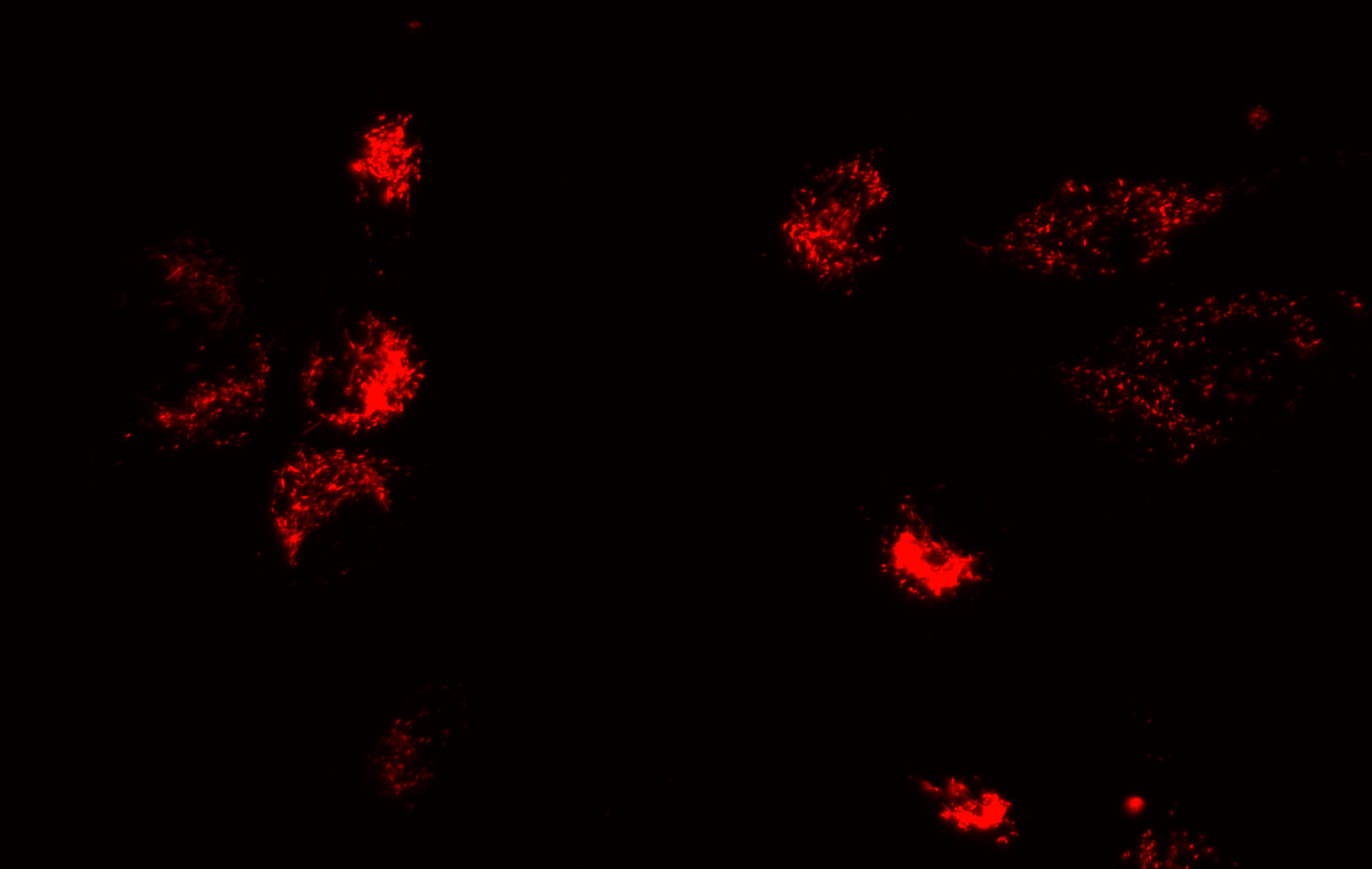

Supplement: Supplementary file 4 [file Data_Sheet_4.ZIP › 4-mitoSOX/2-HO.tif]

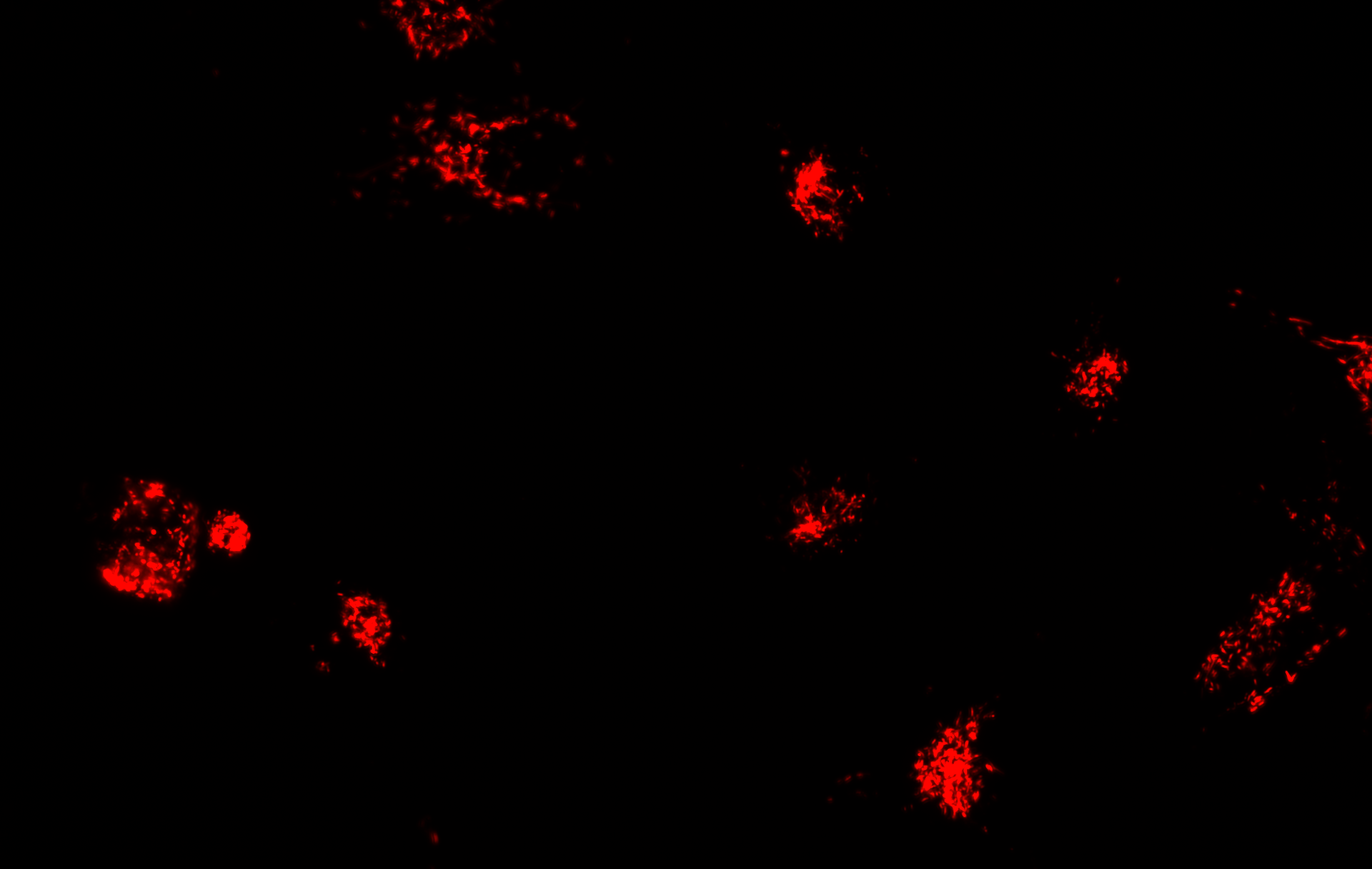

Supplement: Supplementary file 4 [file Data_Sheet_4.ZIP › 4-mitoSOX/2-NG.tif]

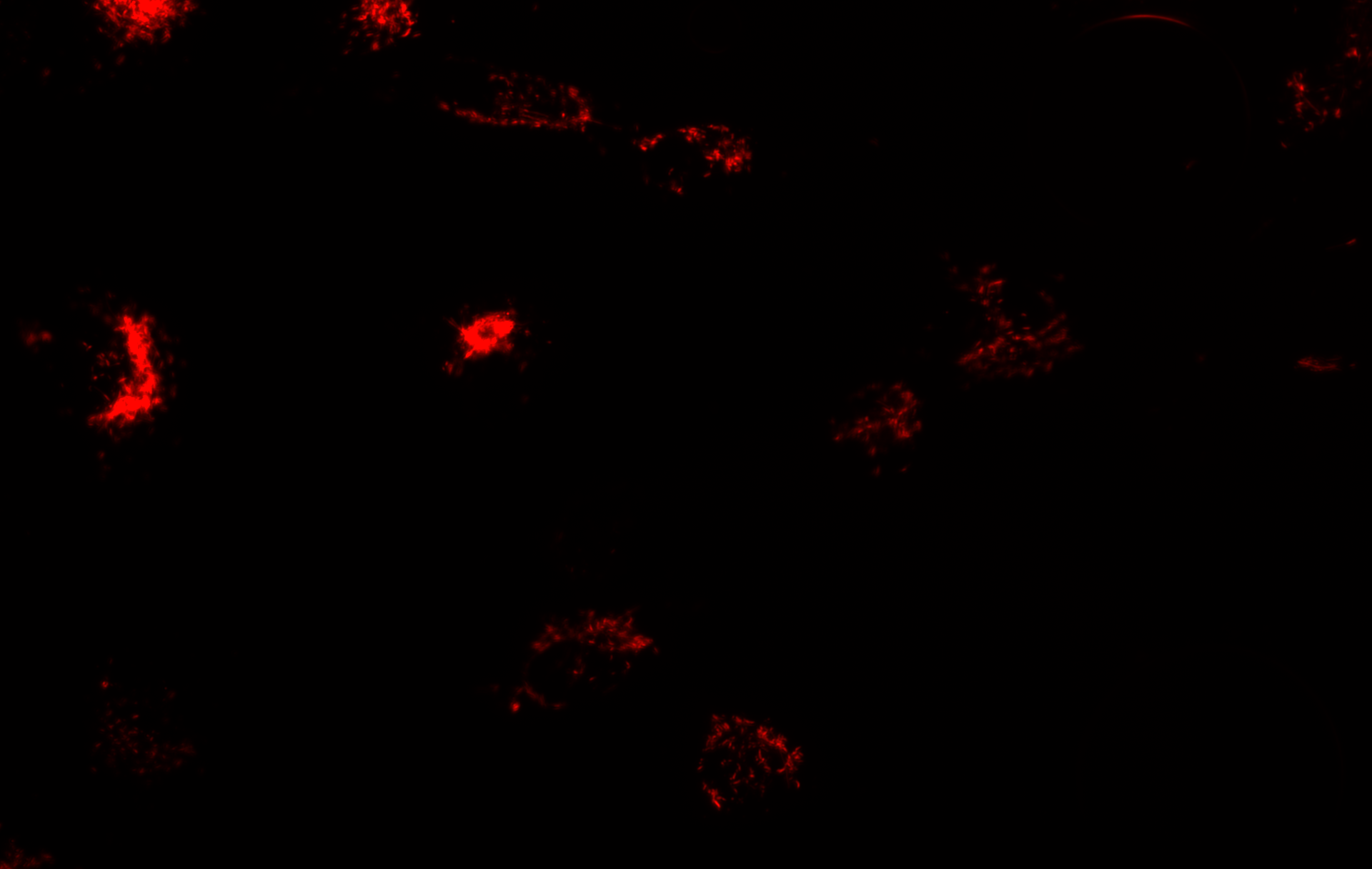

Supplement: Supplementary file 4 [file Data_Sheet_4.ZIP › 4-mitoSOX/HG+NAC+YC-1.tif]

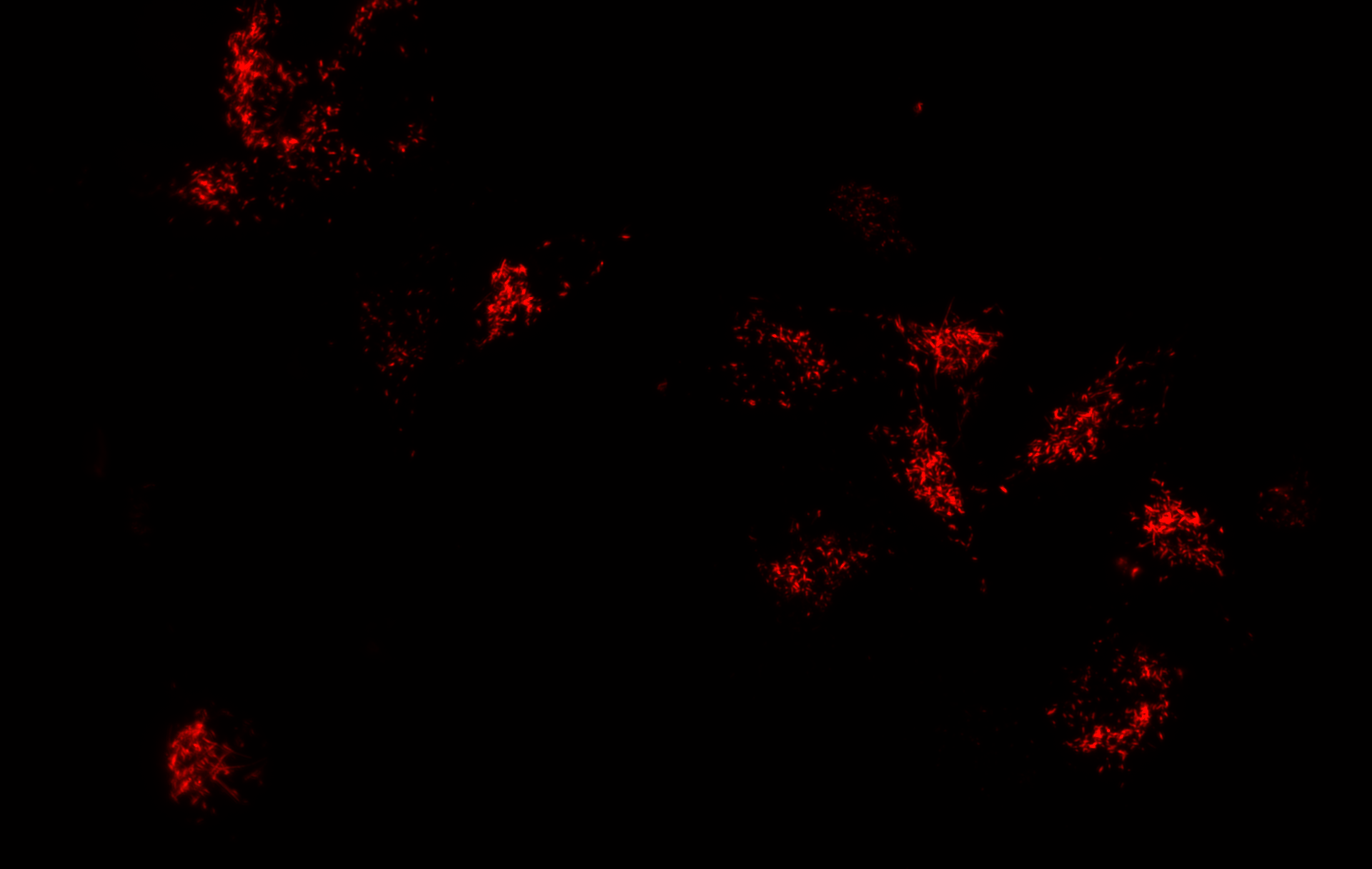

Supplement: Supplementary file 4 [file Data_Sheet_4.ZIP › 4-mitoSOX/HG+NAC.tif]

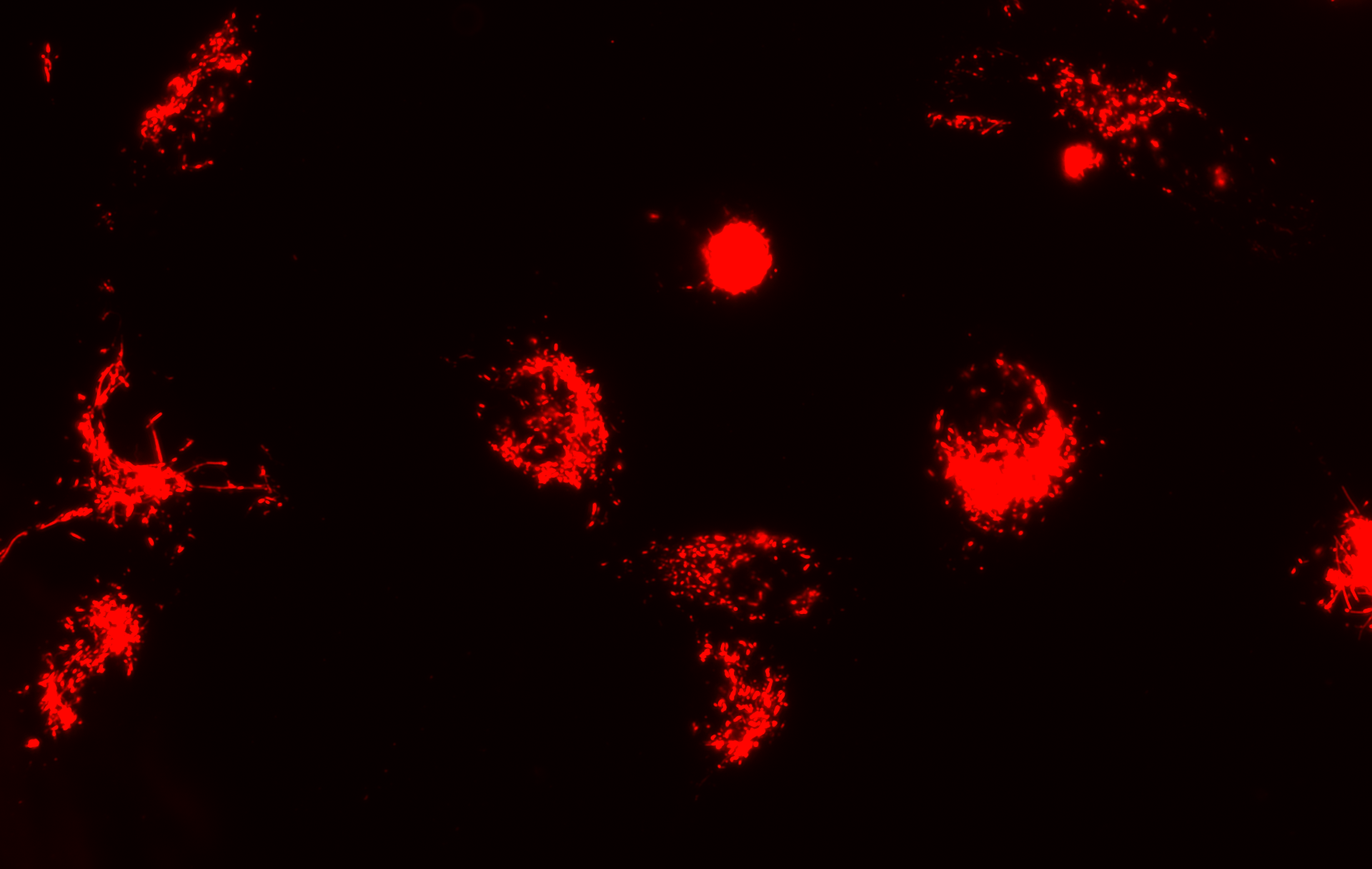

Supplement: Supplementary file 4 [file Data_Sheet_4.ZIP › 4-mitoSOX/HG+YC-1.tif]

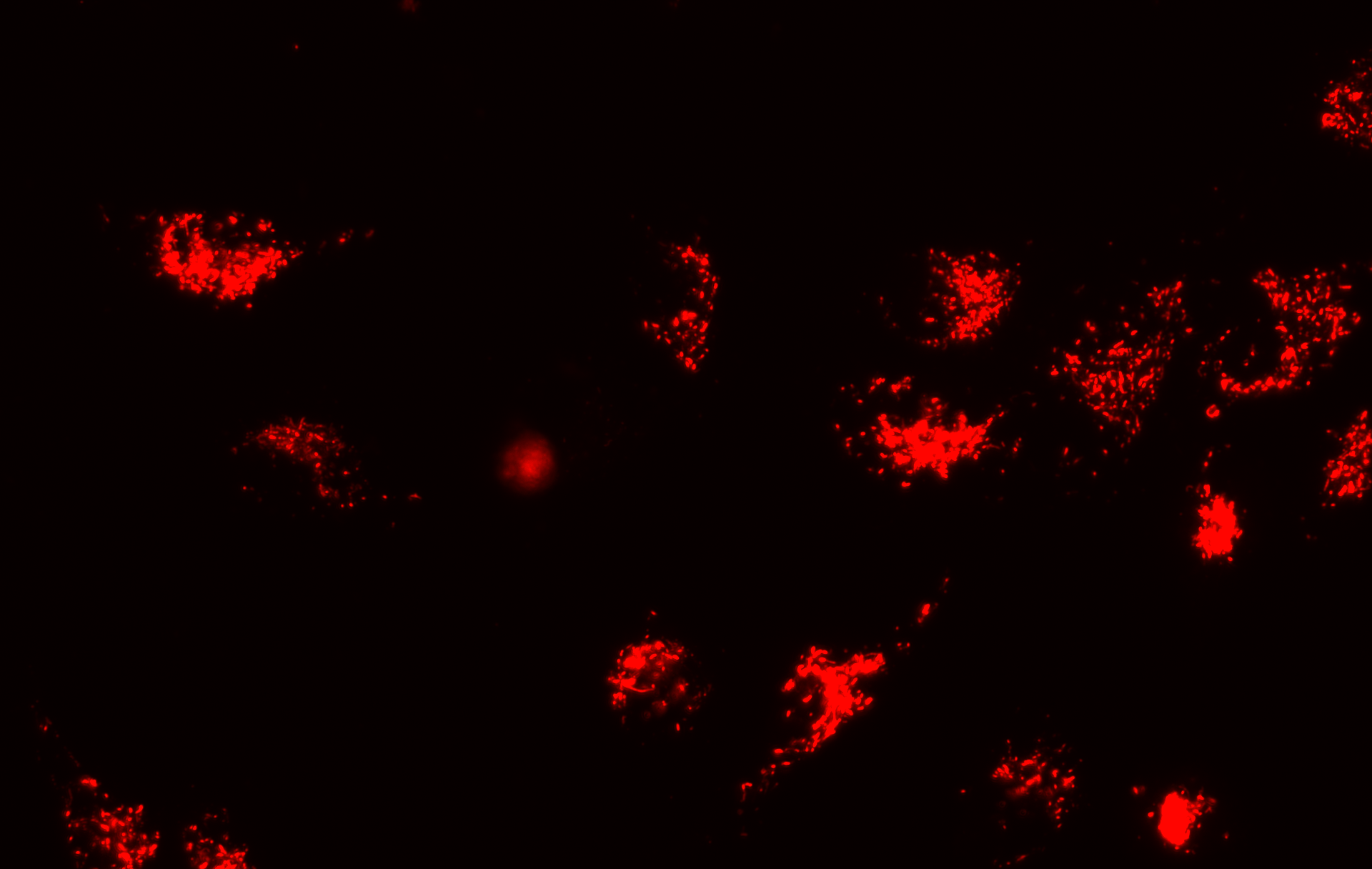

Supplement: Supplementary file 4 [file Data_Sheet_4.ZIP › 4-mitoSOX/HG.tif]

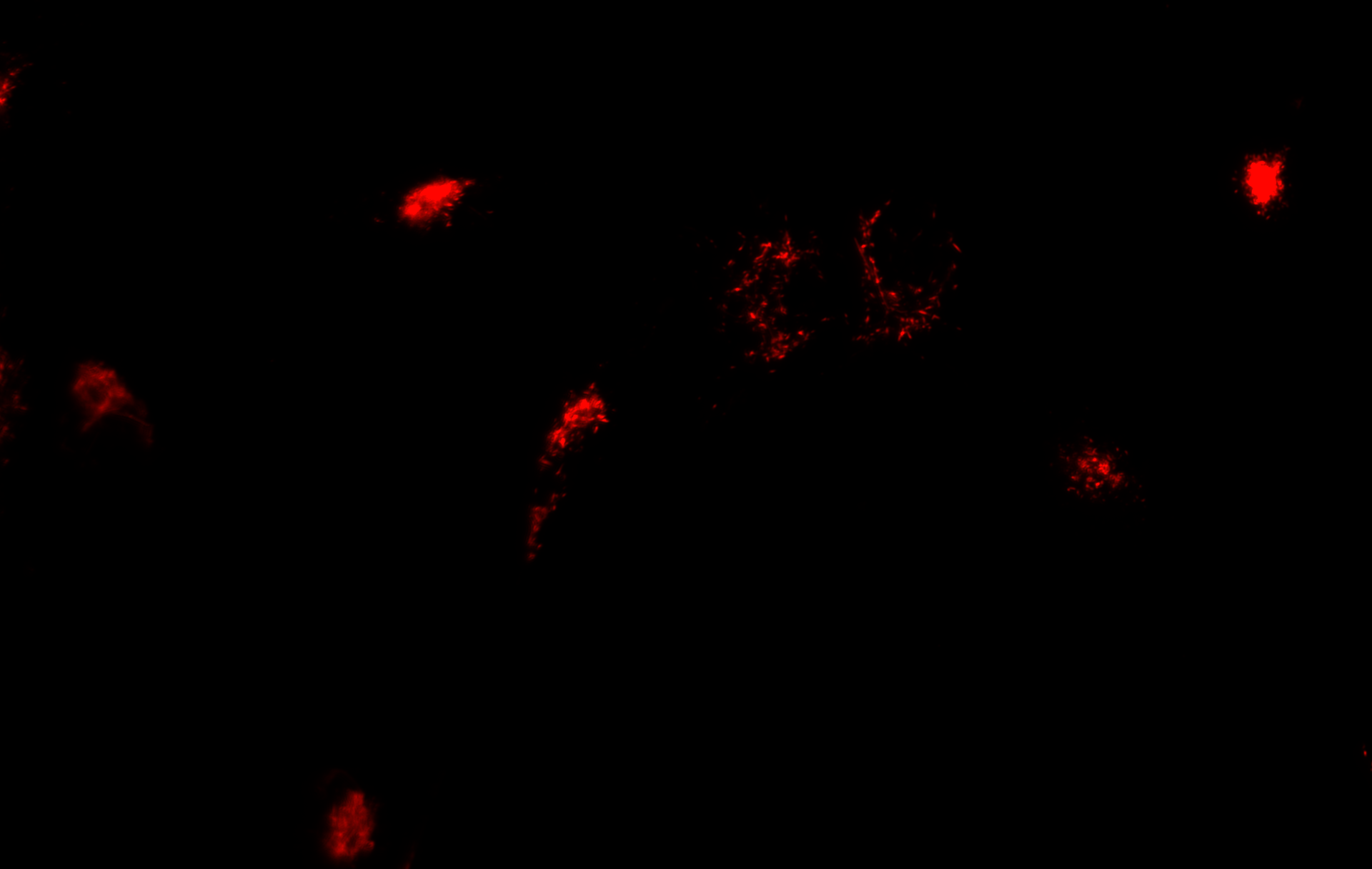

Supplement: Supplementary file 4 [file Data_Sheet_4.ZIP › 4-mitoSOX/NG+NAC.tif]

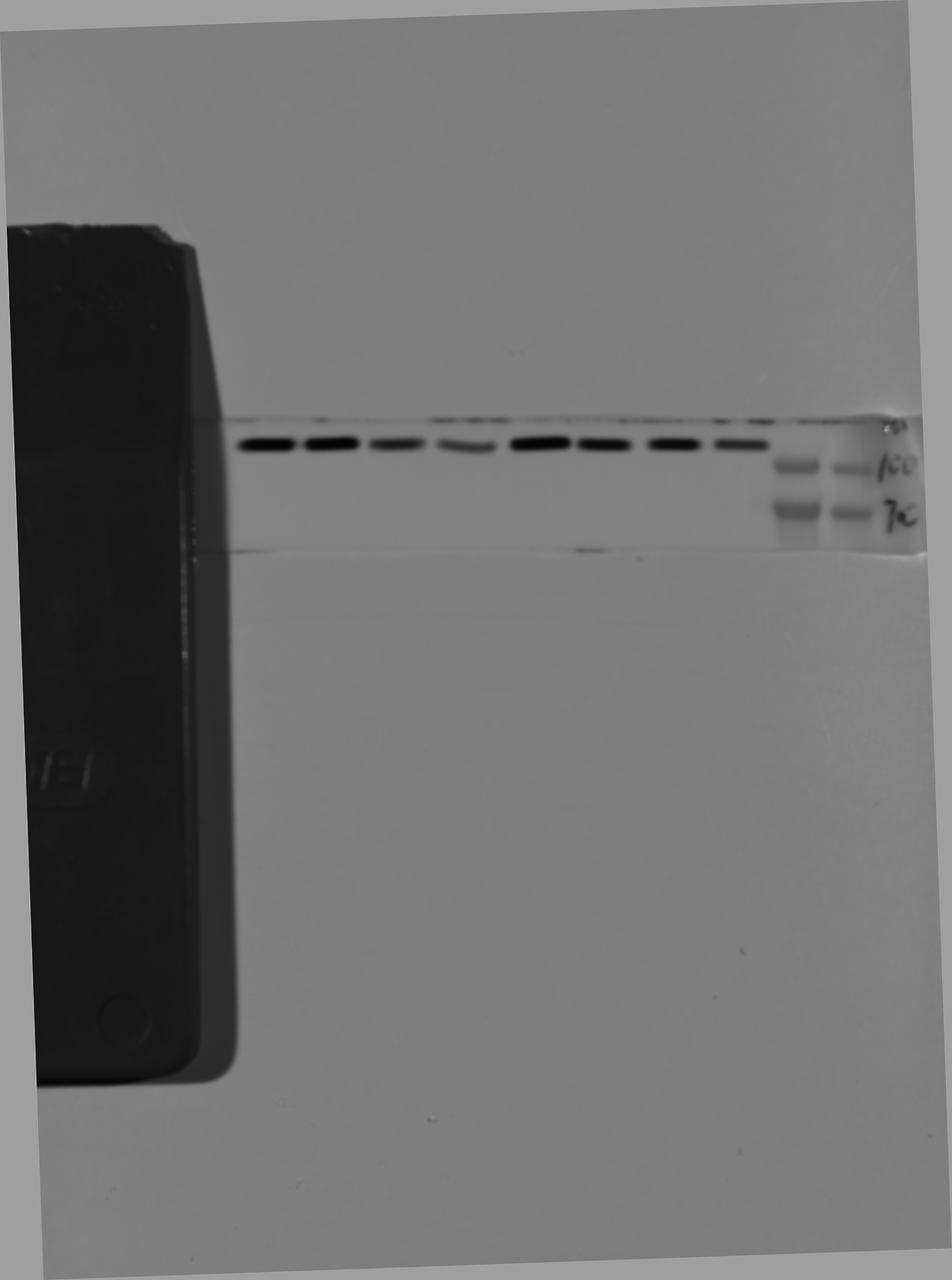

Supplement: Supplementary file 6 [file Data_Sheet_6.ZIP › 6-EMT marker wb/Ch-E-cadherin/Ch-Marker-E-cadherin.tif]

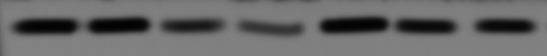

Supplement: Supplementary file 6 [file Data_Sheet_6.ZIP › 6-EMT marker wb/Ch-E-cadherin/E-cadherin.tif]

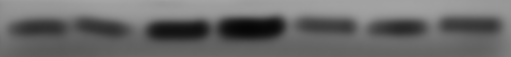

Supplement: Supplementary file 6 [file Data_Sheet_6.ZIP › 6-EMT marker wb/Ch-FN/FN.tif]

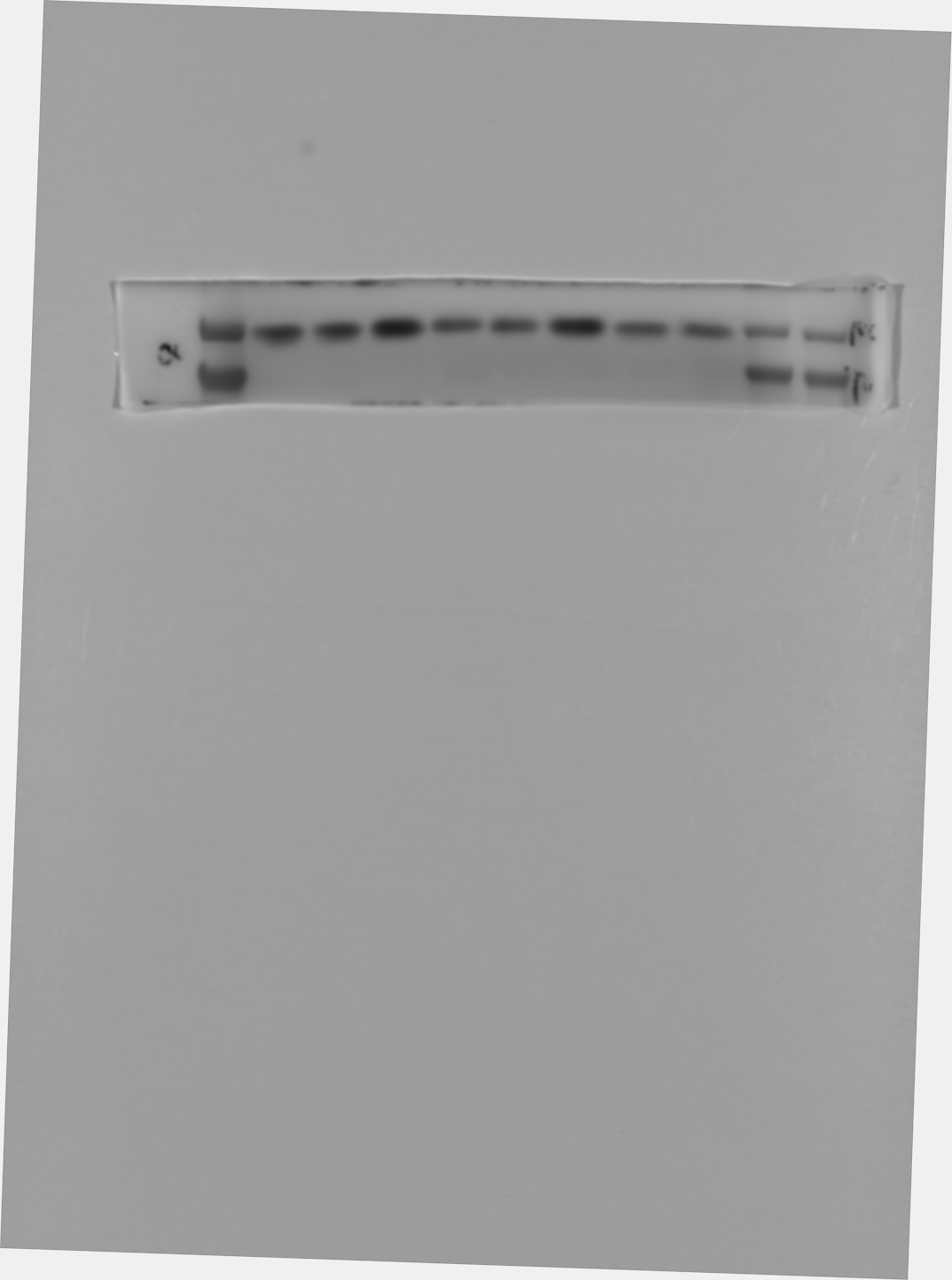

Supplement: Supplementary file 6 [file Data_Sheet_6.ZIP › 6-EMT marker wb/Ch-HIF1a/Ch-Marker-HIF1a.tif]

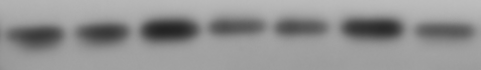

Supplement: Supplementary file 6 [file Data_Sheet_6.ZIP › 6-EMT marker wb/Ch-HIF1a/HIF1a.tif]

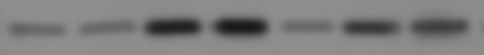

Supplement: Supplementary file 6 [file Data_Sheet_6.ZIP › 6-EMT marker wb/Ch-HIF2a/HIF2a.tif]

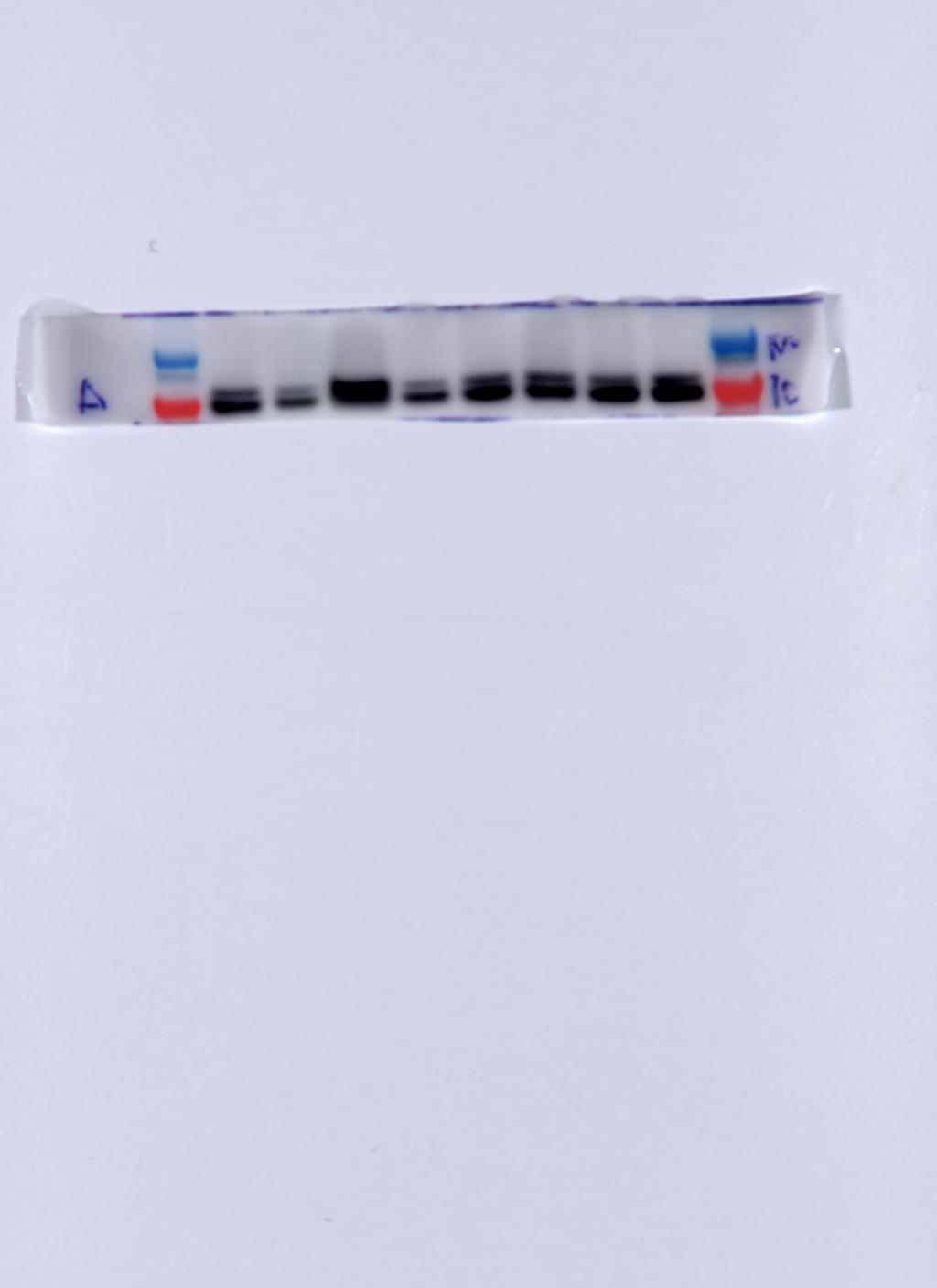

Supplement: Supplementary file 6 [file Data_Sheet_6.ZIP › 6-EMT marker wb/Ch-HIF2a/aa-pgc1a 2021.03.22_12.56.43_Ch+Marker.jpg]

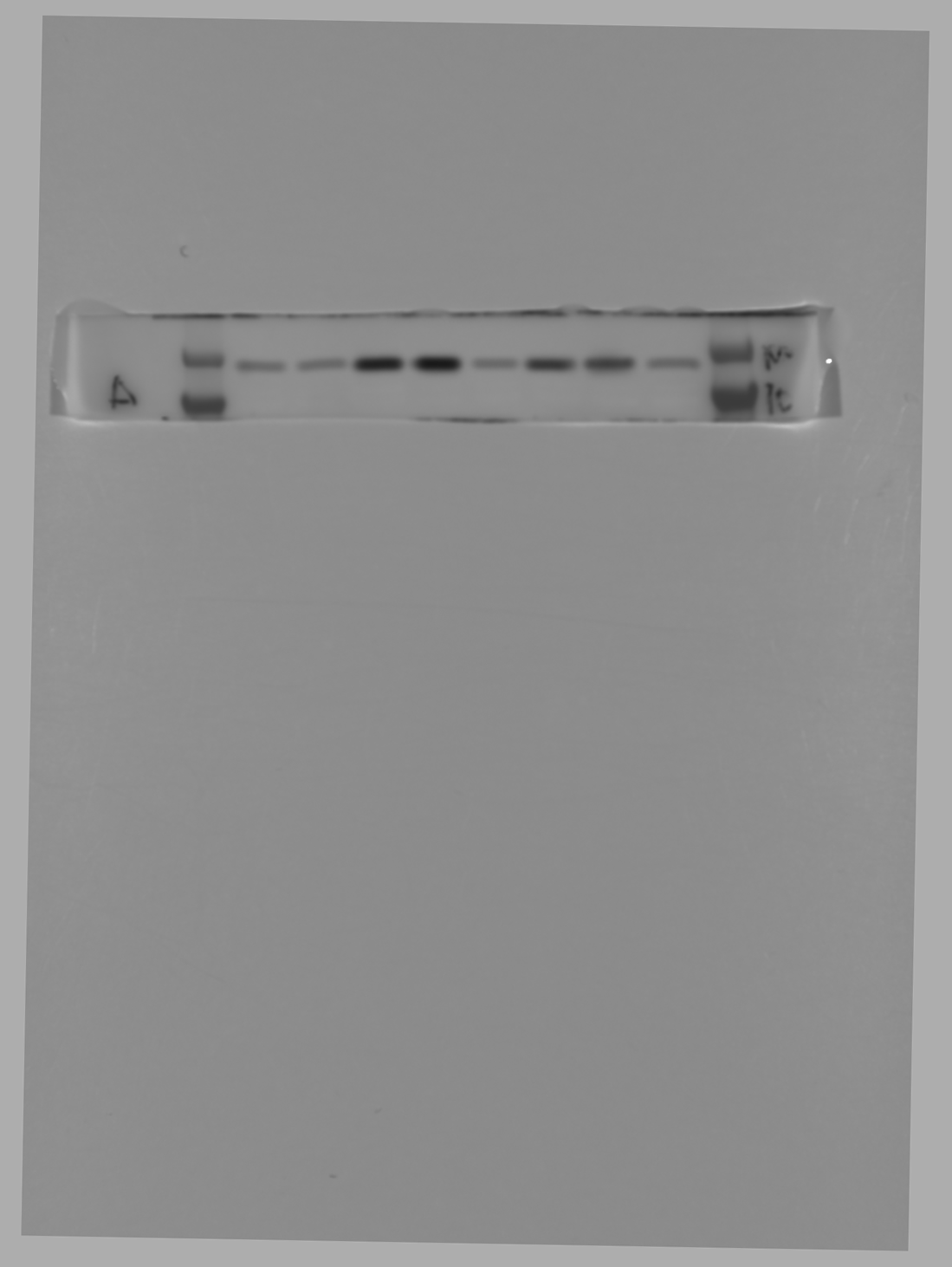

Supplement: Supplementary file 6 [file Data_Sheet_6.ZIP › 6-EMT marker wb/Ch-HIF2a/aa-pgc1a 2021.03.22_12.56.43_Ch-Marker-HIF2a.tif]

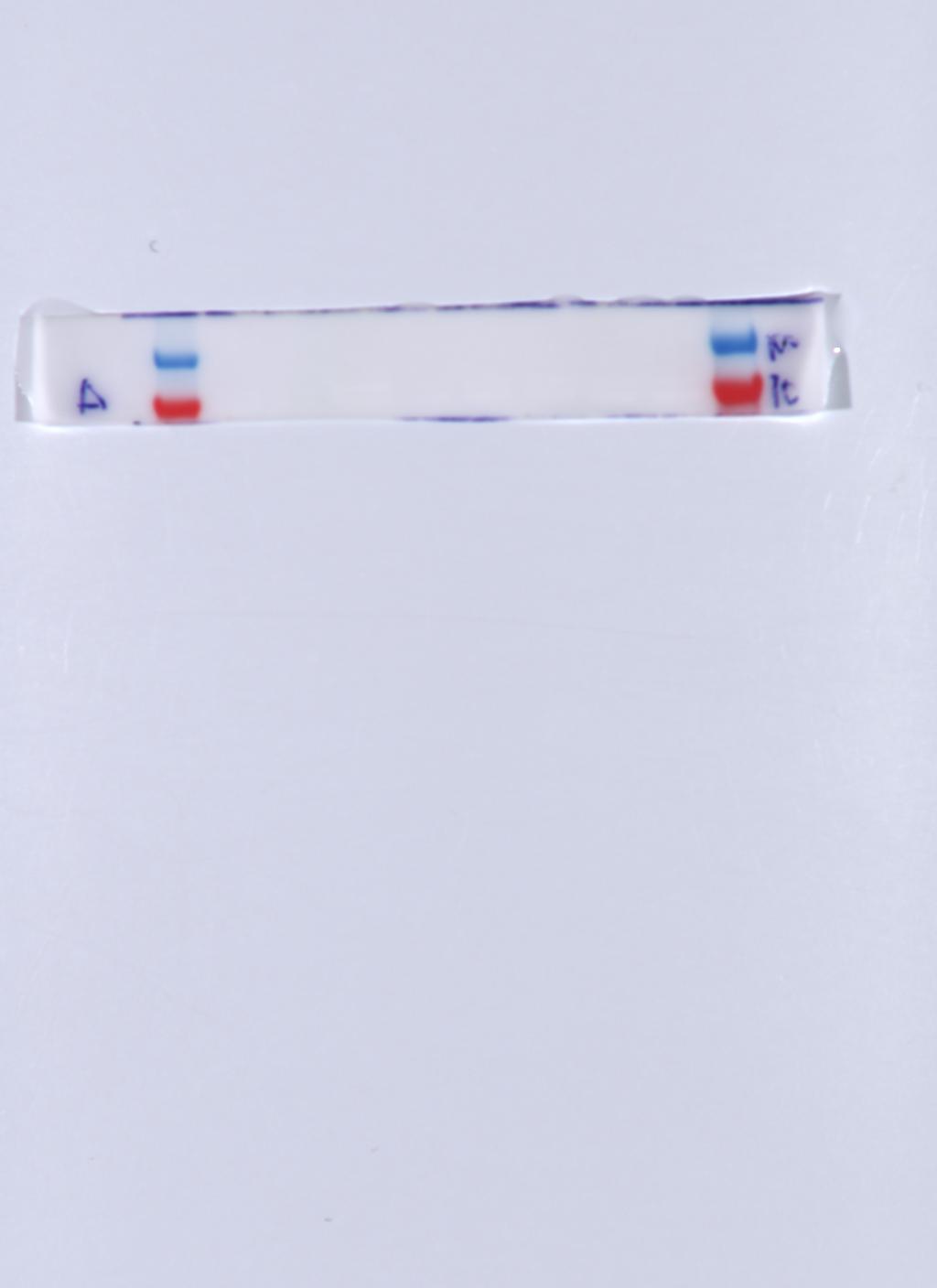

Supplement: Supplementary file 6 [file Data_Sheet_6.ZIP › 6-EMT marker wb/Ch-HIF2a/aa-pgc1a 2021.03.22_12.56.43_Ch-Marker.jpg]

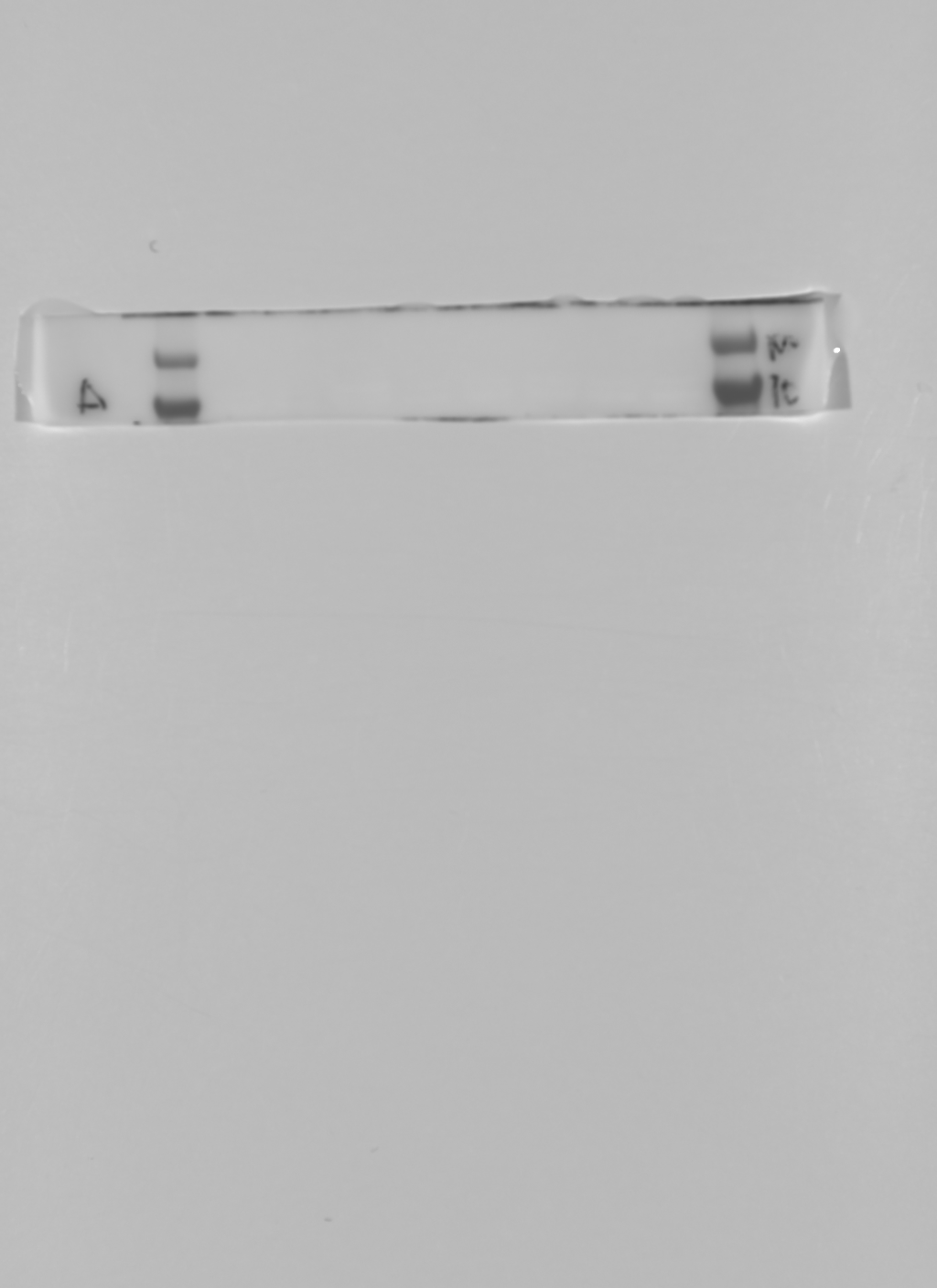

Supplement: Supplementary file 6 [file Data_Sheet_6.ZIP › 6-EMT marker wb/Ch-HIF2a/aa-pgc1a 2021.03.22_12.56.43_Ch-Marker.tif]

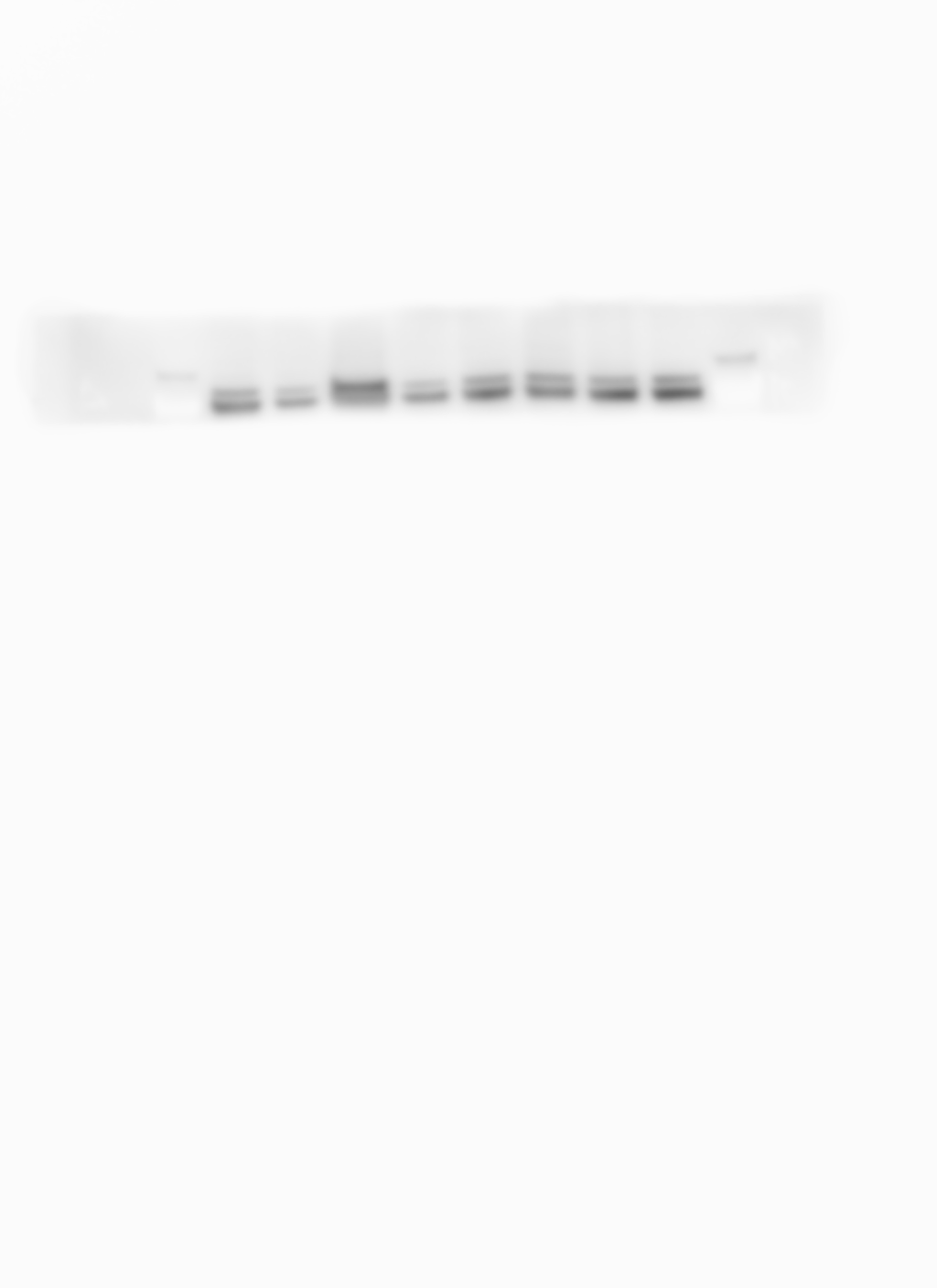

Supplement: Supplementary file 6 [file Data_Sheet_6.ZIP › 6-EMT marker wb/Ch-HIF2a/aa-pgc1a 2021.03.22_12.56.43_Ch.tif]

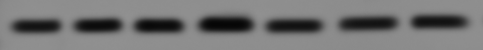

Supplement: Supplementary file 6 [file Data_Sheet_6.ZIP › 6-EMT marker wb/Ch-Marker-b-Actin/Actin-2.tif]

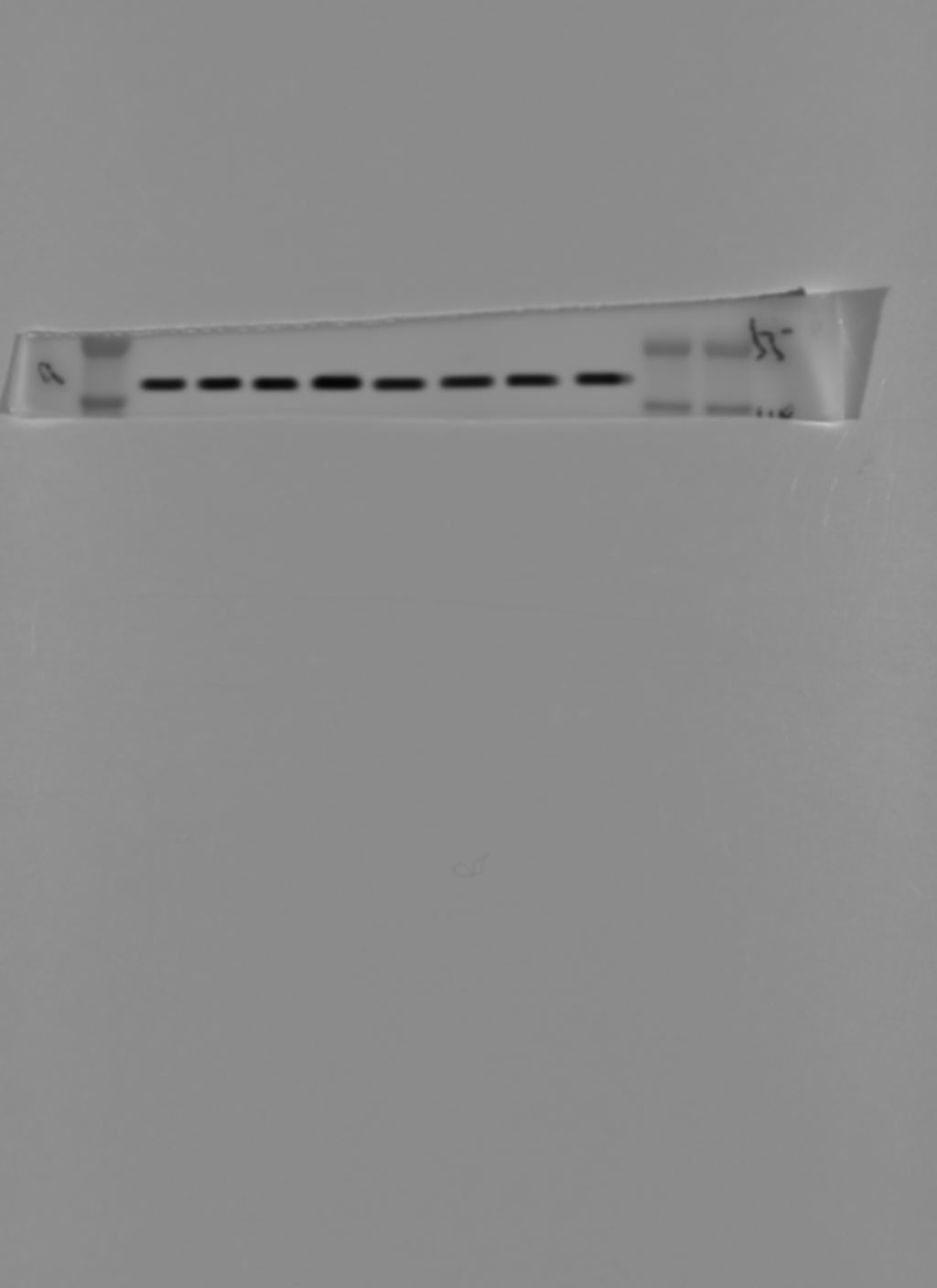

Supplement: Supplementary file 6 [file Data_Sheet_6.ZIP › 6-EMT marker wb/Ch-Marker-b-Actin/Ch-Marker-b-Actin-2.tif]

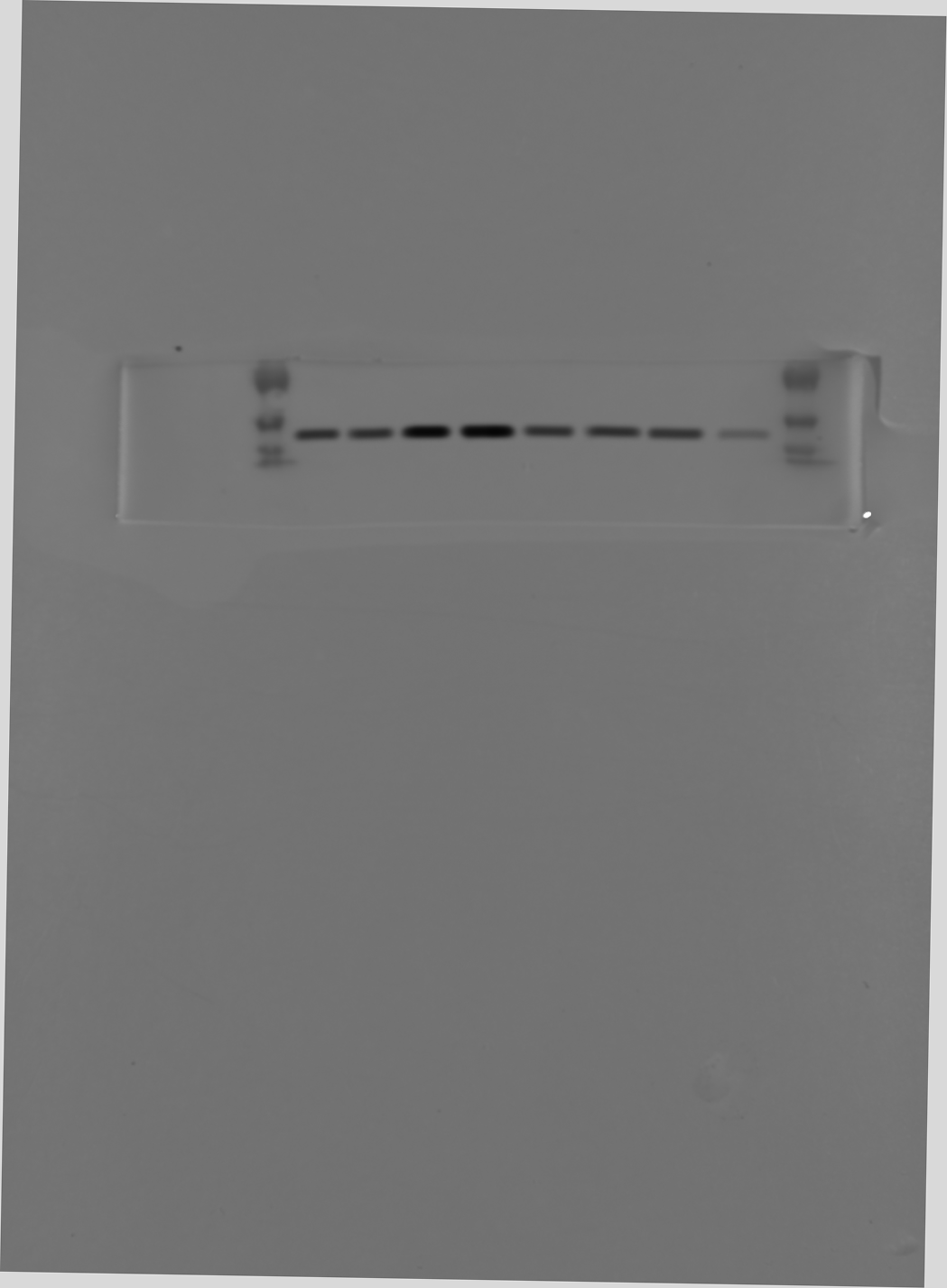

Supplement: Supplementary file 6 [file Data_Sheet_6.ZIP › 6-EMT marker wb/Ch-SGLT2/Ch-Marker-SGLT2.tif]

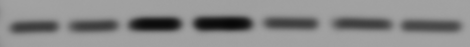

Supplement: Supplementary file 6 [file Data_Sheet_6.ZIP › 6-EMT marker wb/Ch-SGLT2/SGLT2.tif]

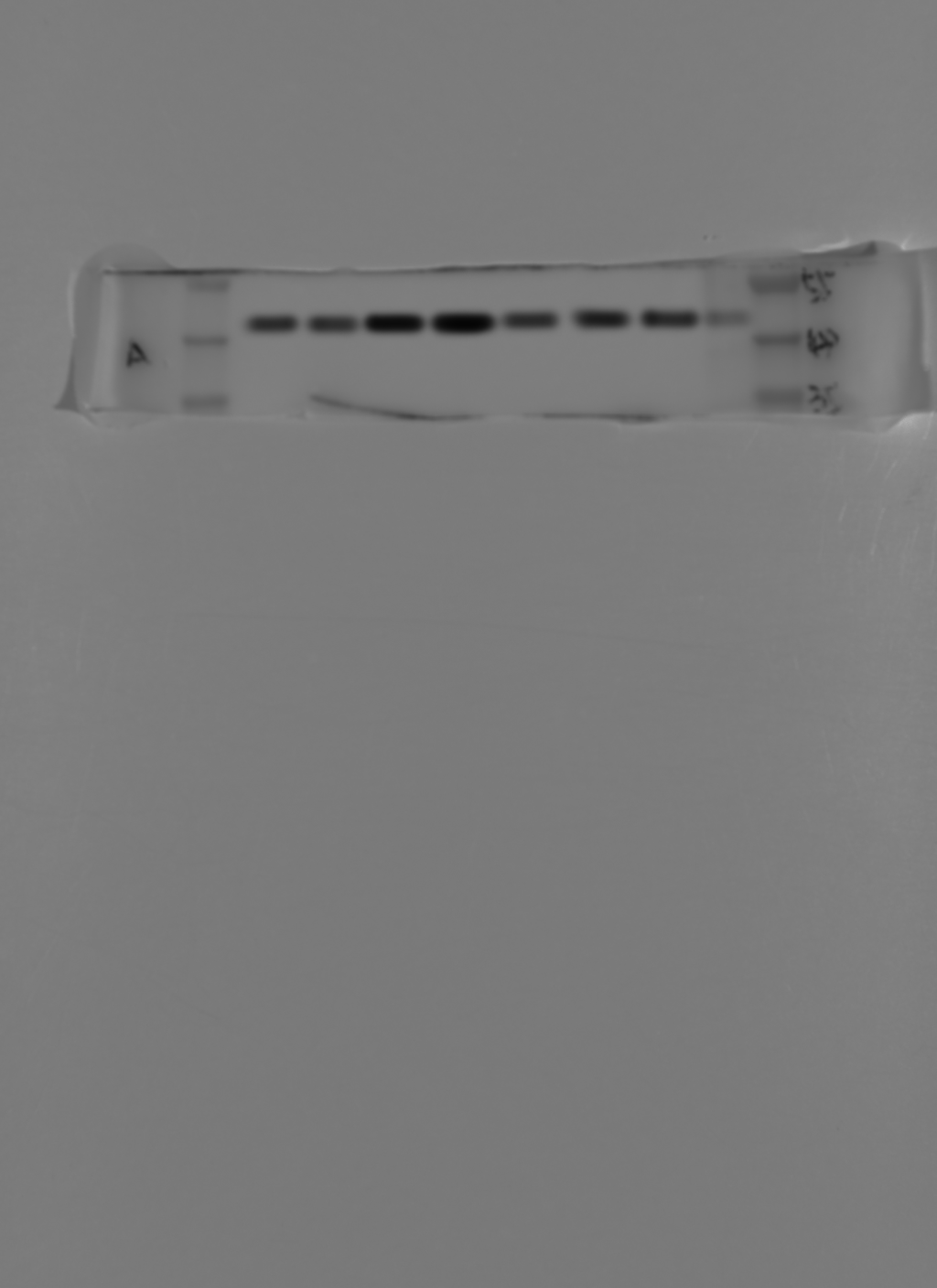

Supplement: Supplementary file 6 [file Data_Sheet_6.ZIP › 6-EMT marker wb/Ch-a-SMA/Ch-Marker-a-SMA.tif]

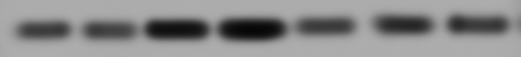

Supplement: Supplementary file 6 [file Data_Sheet_6.ZIP › 6-EMT marker wb/Ch-a-SMA/a-SMA.tif]
