# Supplementary material for: HIF-1α Alleviates High-Glucose-Induced Renal Tubular Cell Injury by Promoting Parkin/PINK1-Mediated Mitophagy
Source: Front Med (Lausanne). 2022 Feb 3;8:803874. doi: 10.3389/fmed.2021.803874 (PMC8850720; doi:10.3389/fmed.2021.803874)
Supplement: Supplementary file 3 [file Data_Sheet_3.ZIP › 3-apoptosis/3-apop original data.pptx]

## Slide 1
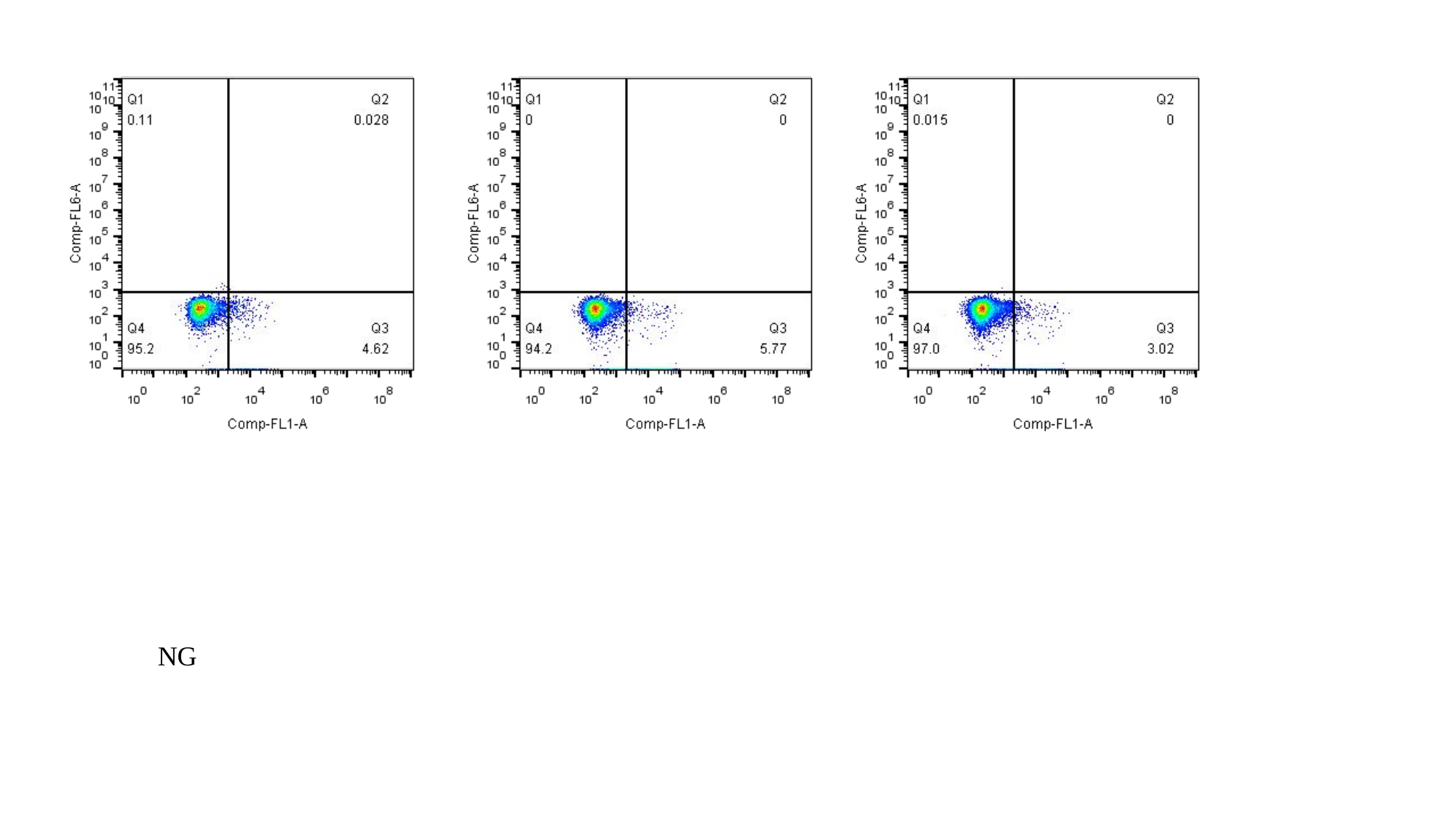

NG

## Slide 2
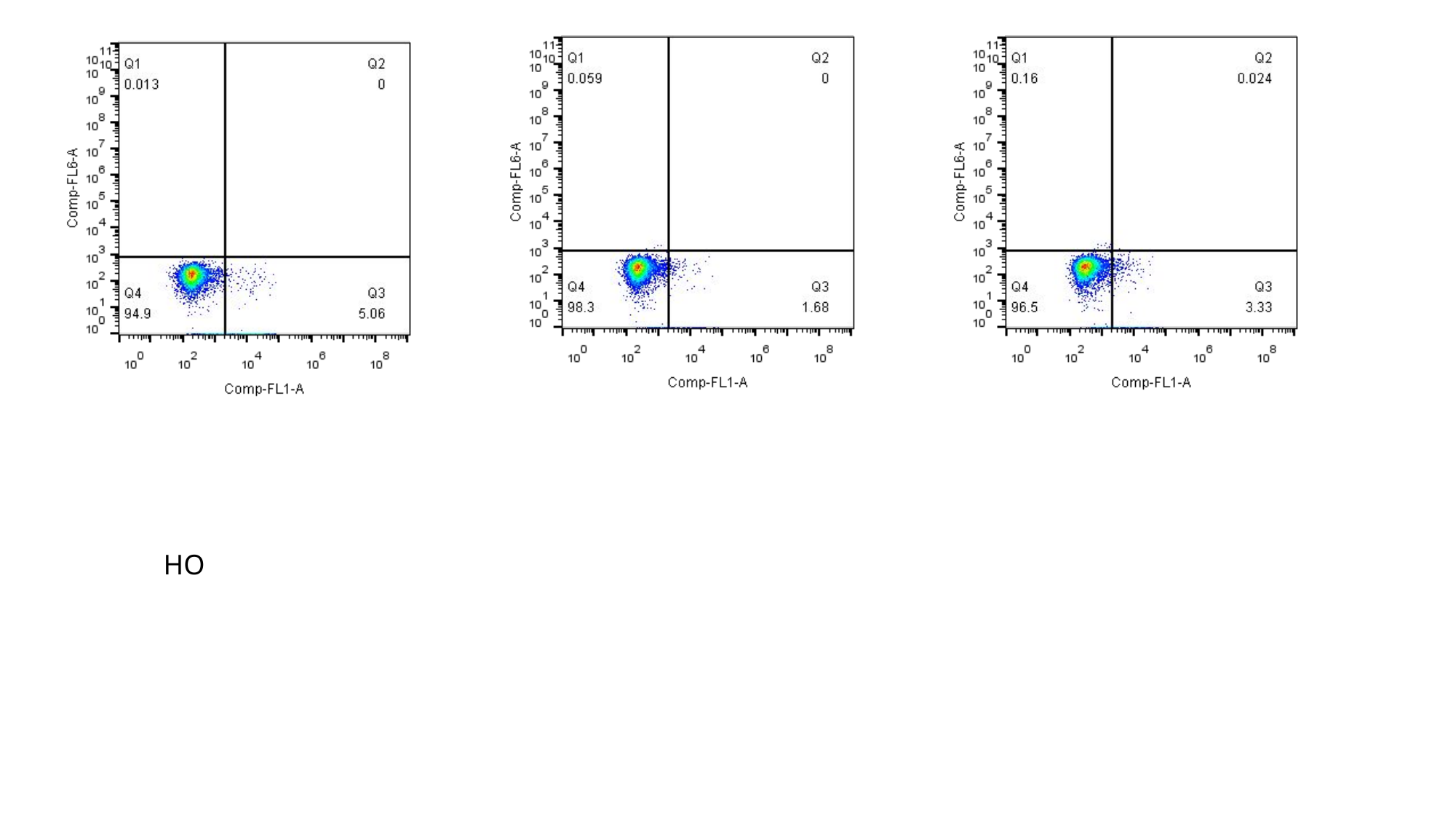

HO

## Slide 3
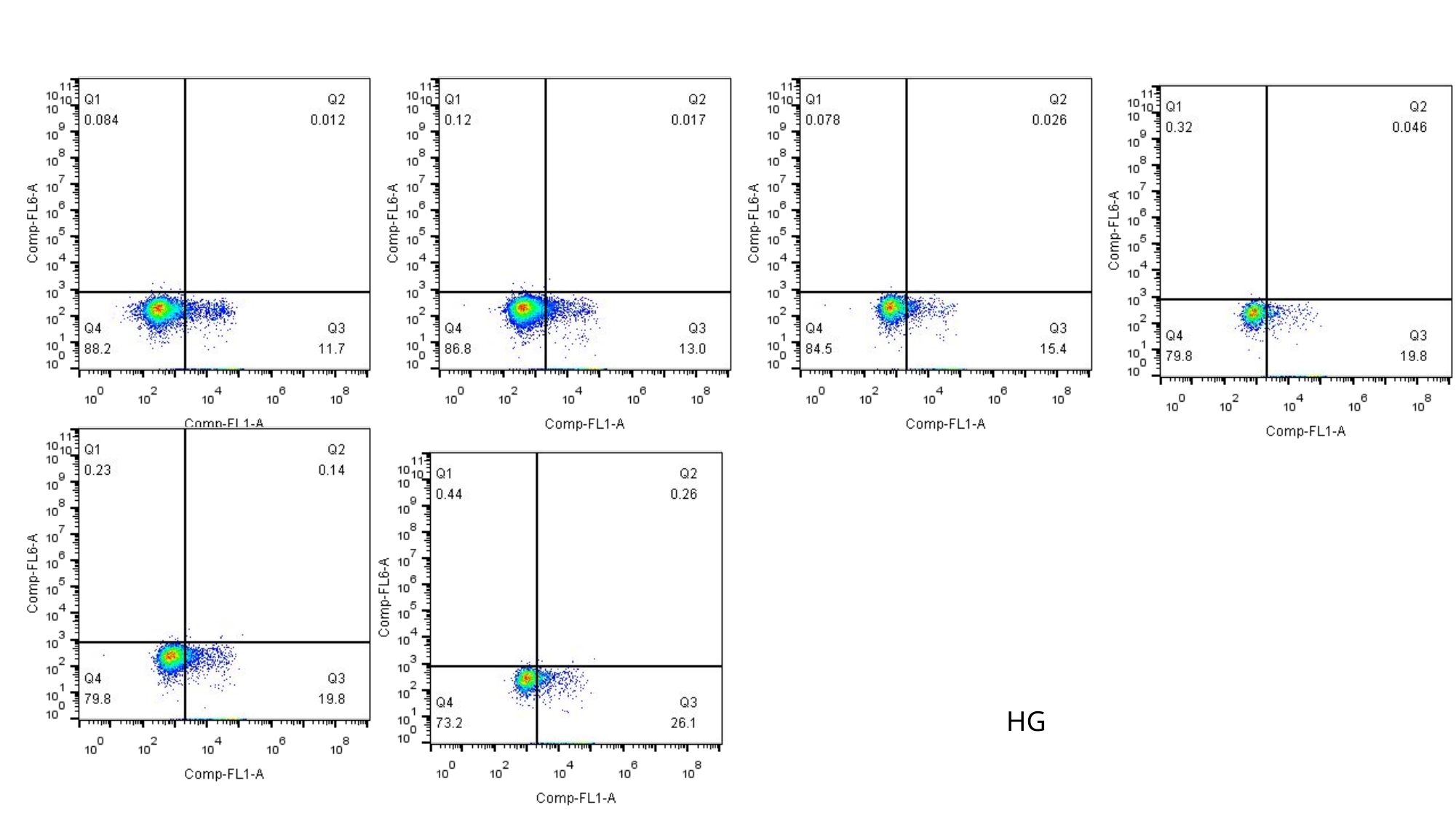

HG

## Slide 4
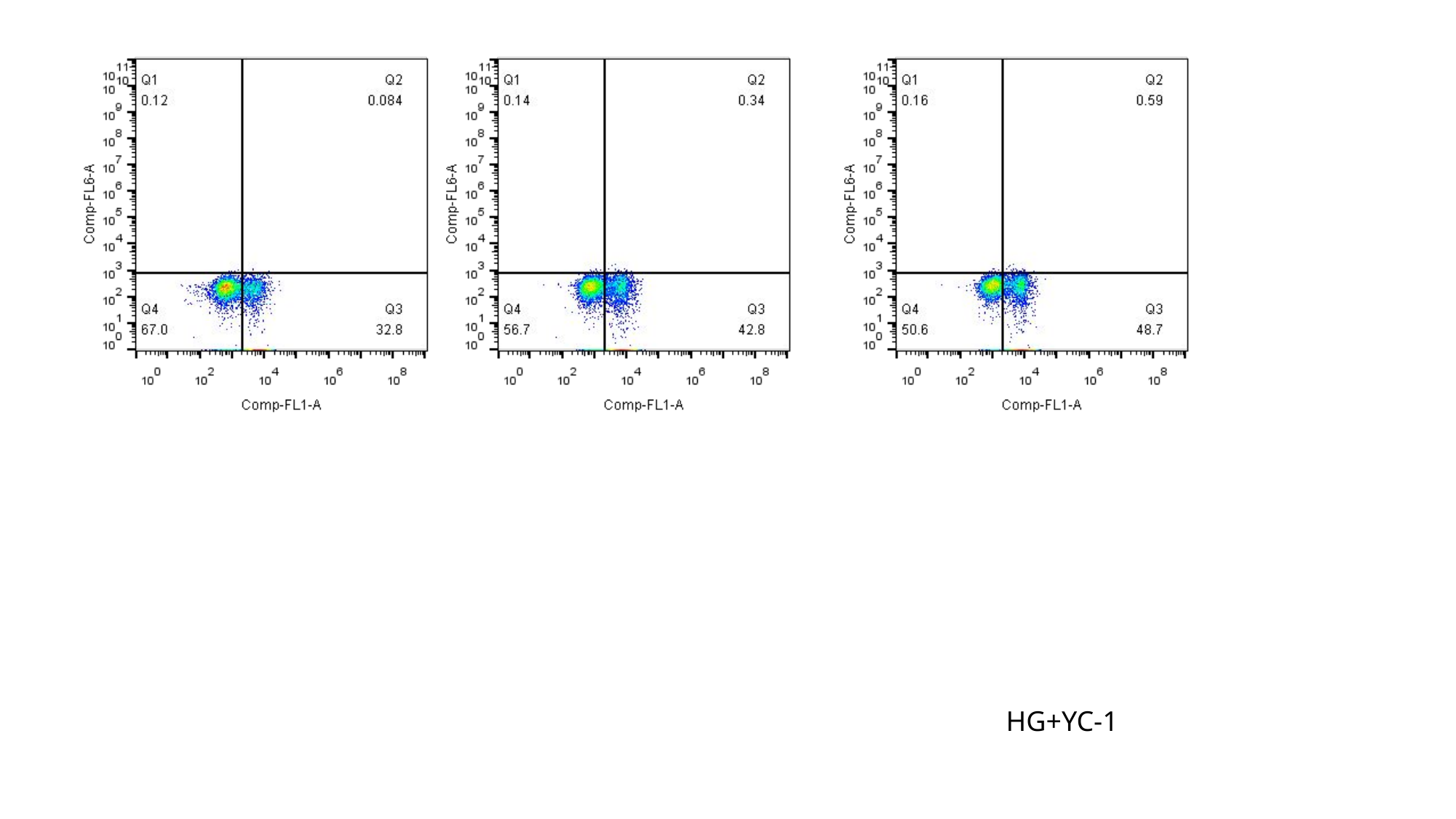

HG+YC-1

## Slide 5
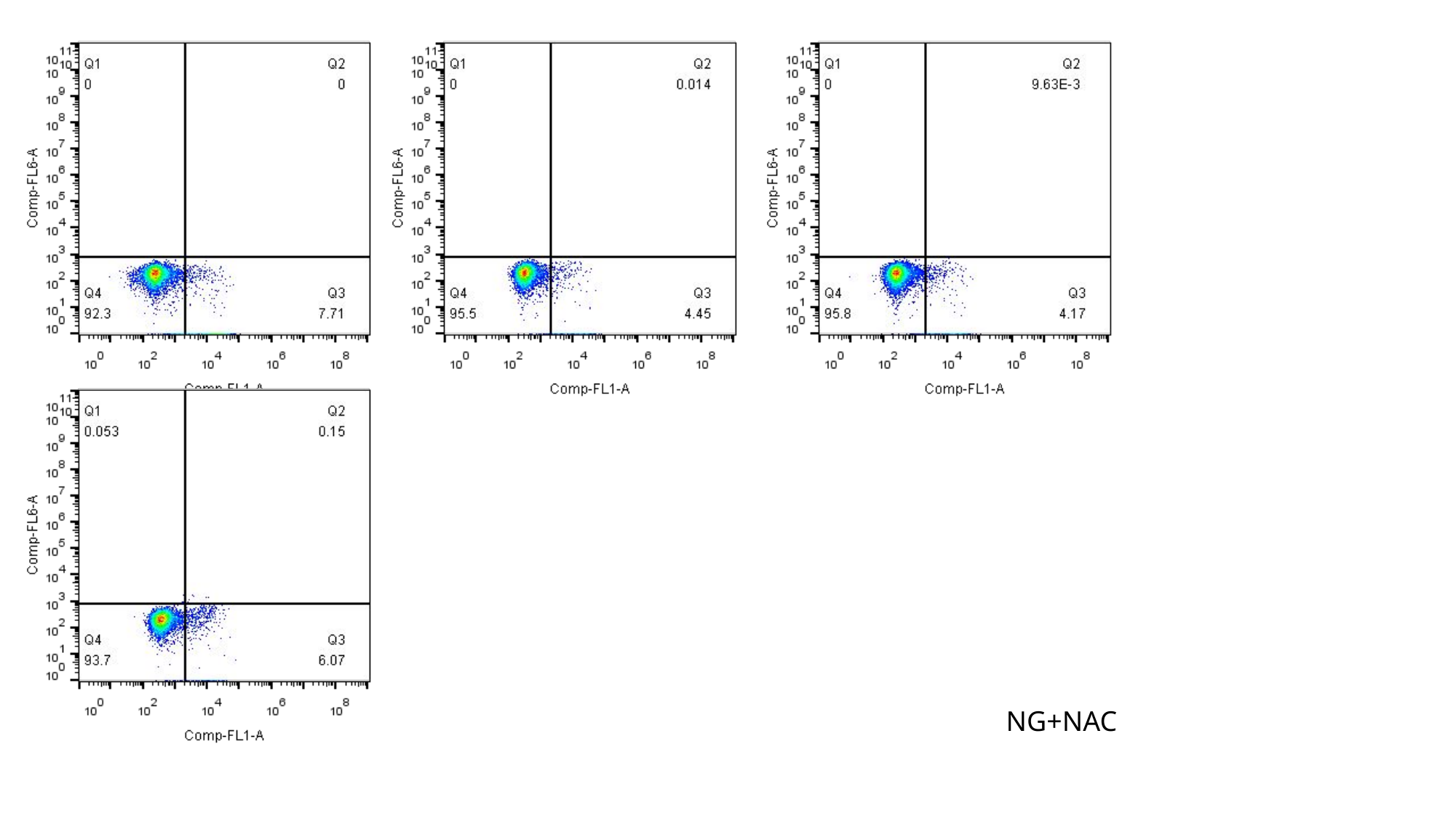

NG+NAC

## Slide 6
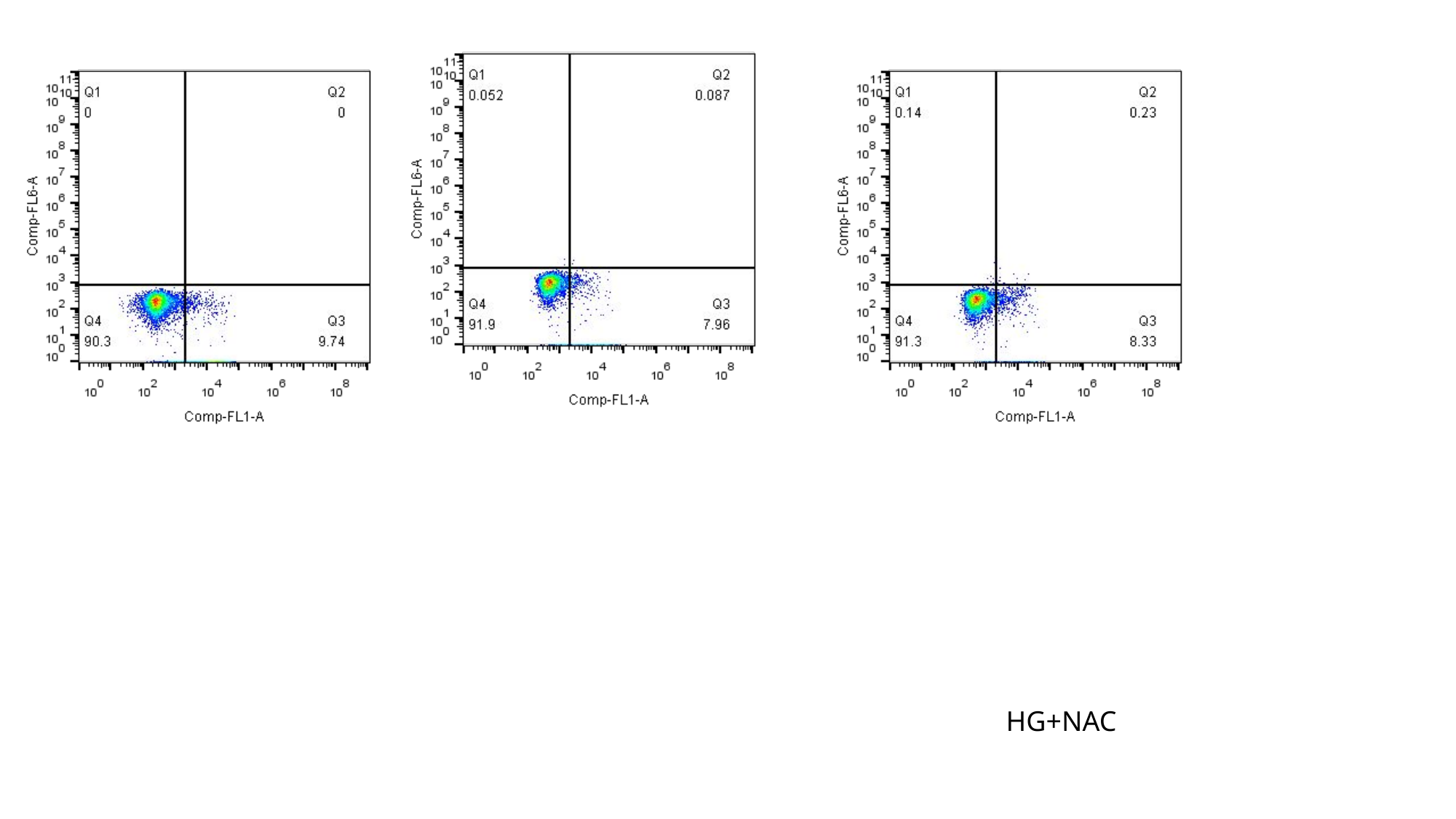

HG+NAC

## Slide 7
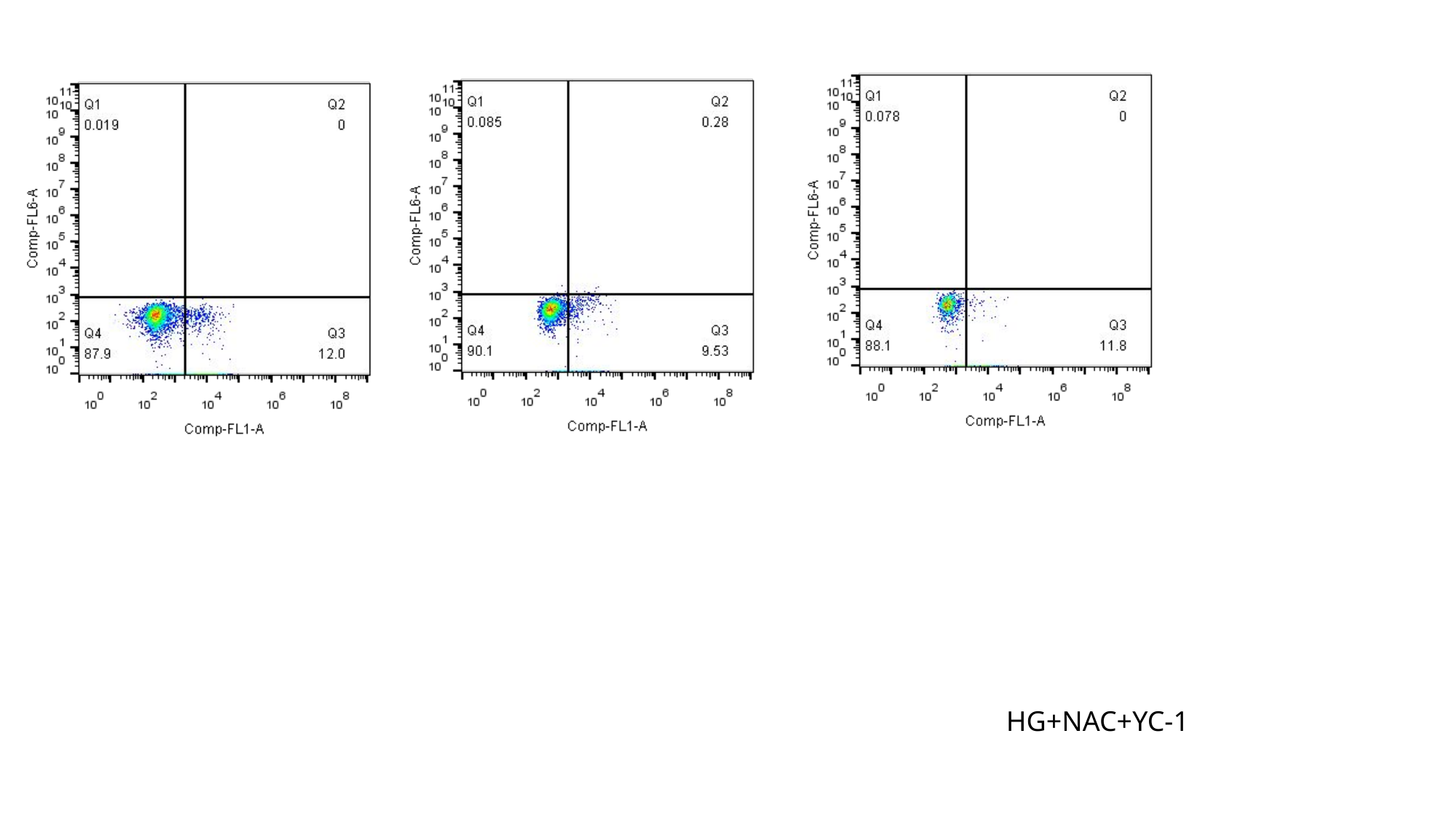

HG+NAC+YC-1

## Slide 8
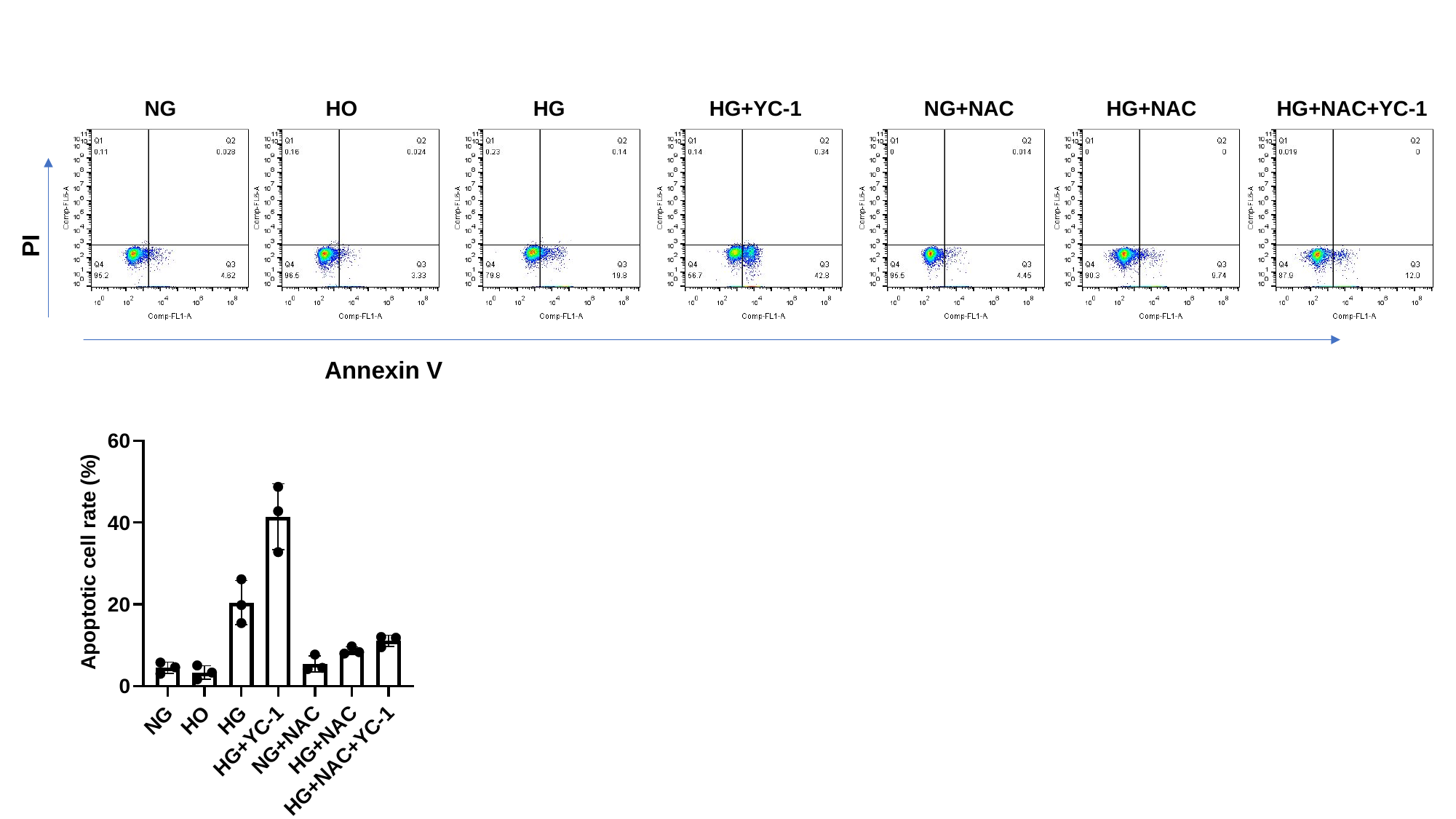

NG
HO
HG
HG+YC-1
NG+NAC
HG+NAC
HG+NAC+YC-1
PI
Annexin V
